# Contemporary epidemiological data of Rift Valley fever virus in humans, mosquitoes and other animal species in Africa: A systematic review and meta‐analysis

**DOI:** 10.1002/vms3.1238

**Published:** 2023-08-07

**Authors:** Jean Thierry Ebogo‐Belobo, Sebastien Kenmoe, Ngu Njei Abanda, Arnol Bowo‐Ngandji, Donatien Serge Mbaga, Jeannette Nina Magoudjou‐Pekam, Ginette Irma Kame‐Ngasse, Serges Tchatchouang, Elisabeth Zeuko'o Menkem, Etienne Atenguena Okobalemba, Efietngab Atembeh Noura, Dowbiss Meta‐Djomsi, Martin Maïdadi‐Foudi, Josiane Kenfack‐Zanguim, Raoul Kenfack‐Momo, Cyprien Kengne‐Nde, Seraphine Nkie Esemu, Wilfred Fon Mbacham, Serge Alain Sadeuh‐Mba, Lucy Ndip, Richard Njouom

**Affiliations:** ^1^ Centre for Research on Health and Priority Pathologies Institute of Medical Research and Medicinal Plants Studies Yaounde Cameroon; ^2^ Department of Biochemistry Faculty of Sciences The University of Yaounde I Yaoundé Cameroon; ^3^ Department of Microbiology and Parasitology University of Buea Buea Cameroon; ^4^ Virology Department Centre Pasteur of Cameroon Yaoundé Cameroon; ^5^ Department of Microbiology Faculty of Sciences The University of Yaounde I Yaoundé Cameroon; ^6^ Bacteriology Department Centre Pasteur of Cameroon Yaounde Cameroon; ^7^ Department of Biomedical Sciences University of Buea Buea Cameroon; ^8^ Faculty of Medicine and Biomedical Sciences The University of Yaoundé I Yaoundé Cameroon; ^9^ Research Centre on Emerging and Re‐Emerging Diseases Institute of Medical Research and Medicinal Plants Studies Yaounde Cameroon; ^10^ Epidemiological Surveillance, Evaluation and Research Unit National AIDS Control Committee Yaoundé Cameroon; ^11^ Maryland Department of Agriculture Salisbury Animal Health Laboratory Salisbury Maryland USA

**Keywords:** Africa, animals, humans, mosquitoes, Rift Valley fever virus

## Abstract

Rift Valley fever (RVF) is a severe zoonotic mosquito‐borne disease that represents an important threat to human and animal health, with major public health and socioeconomic impacts. This disease is endemic throughout many African countries and the Arabian Peninsula. This systematic review with meta‐analysis was conducted to determine the RVF prevalence in humans, mosquitoes and other animal species in Africa. The review also provides contemporary data on RVF case fatality rate (CFR) in humans. In this systematic review with meta‐analysis, a comprehensive literature search was conducted on the PubMed, Embase, Web of Science and Global Index Medicus databases from January 2000 to June 2022 to identify relevant studies. Pooled CFR and prevalence estimates were calculated using the random‐effects model. Subgroup analysis and sensitivity analysis were performed, and the *I* ^2^‐statistic was used to investigate a potential source of heterogeneity. A total of 205 articles were included in the final analysis. The overall RVF CFR in humans was found to be 27.5% [95% CI = 8.0–52.5]. The overall pooled prevalence was 7.8% [95% CI = 6.2–9.6] in humans and 9.3% [95% CI = 8.1–10.6] in animals, respectively. The RVF prevalence in individual mosquitoes ranged from 0.0% to 25%. Subgroup analysis showed substantial heterogeneity with respect to geographical regions and human categories. The study shows that there is a correspondingly similar prevalence of RVF in human and animals; however, human CFR is much higher than the observed prevalence. The lack of a surveillance programme and the fact that this virus has subclinical circulation in animals and humans could explain these observations. The implementation of a One Health approach for RVF surveillance and control would be of great interest for human and animal health.

## Graphical abstract

Graphical Abstract: A total of 205 articles were included in the final analysis. The study shows that there is a correspondingly similar prevalence of RVF in human and animals; however, human CFR is much higher than the observed prevalence.

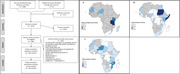

## 1 INTRODUCTION

Rift Valley fever (RVF) is one of the most important human and veterinary arthropod‐borne diseases in Africa. This emerging mosquito‐borne haemorrhagic fever disease, induced by the Rift Valley fever virus (RVFV), causes significant illness and death in humans and animals (Linthicum et al., 2016; Nanyingi et al., 2015). Since its first description, in 1930 in Kenya (Daubney et al., 1931), this disease has become endemic throughout many African countries and in the Arabian Peninsula (Chevalier et al., 2004; Clark et al., 2018; Dungu et al., 2018; Nanyingi et al., 2015). RVF‐associated outbreaks occur periodically after a 5–15‐year interval, usually when ecological and climatic factors favourable for competent vector emergence are established in some epidemiological settings (Glancey et al., 2015; Hightower et al., 2012; Nanyingi et al., 2015).

RVFV is an arbovirus that belongs to the *Phlebovirus* genus in the Phenuiviridae family (Adams et al., 2017; King et al., 2018). This virus is maintained in nature through horizontal transmission between vertebrate hosts and blood‐feeding mosquitoes and vertically, through infected mosquitoes and their offspring (Lumley et al., 2017). Transmission to humans occurs either through bites by a broad range of RVFV‐infected mosquito species, mainly from the *Aedes* and *Culex* genera (Lumley et al., 2017), or through direct contact with body fluids, blood or tissues of viremic animals (Balenghien et al., 2013; Bird et al., 2009; Pepin et al., 2010). RVFV infection is associated with high rates of abortions among pregnant domestic ruminants (mainly sheep, goats and cattle) and can induce a high case fatality rate (CFR) up to 100% in newborn ruminants (Bird et al., 2009). In humans, RVFV infection usually leads to a transient febrile illness with occasional complications that can progress to haemorrhagic syndrome and/or encephalitis which can lead to death (Ikegami & Makino, 2011; Pepin et al., 2010).

Despite its public health importance (Chauhan et al., 2020) and its economic consequences in Africa due to loss in domestic animals (Baba et al., 2016; Peyre et al., 2015; Wanyoike & Rich, 2010; Wright et al., 2019), routine surveillance and monitoring of RVF are very limited in most African countries (Oyas et al., 2018). In such context, RVFV infection may be either missed or misdiagnosed, and even outbreaks are underreported (Grossi‐Soyster & Labeaud, 2020). Although many countries are considered free from RVFV, the international trade of domestic ruminants as well as the presence of known and potentially competent vectors in those countries might provide a suitable environment for the spread of RVFV from endemic to non‐endemic countries (Bird et al., 2009; Pepin et al., 2010). Therefore, up‐to‐date knowledge of RVFV circulation, ecology, amplifying vertebrate hosts and vectors in specific regions and/or populations are critical for the design, evaluation and optimization of RVF surveillance and control programmes. Contemporary data on the RVF epidemiology might also assist research prioritization and preparedness for timely and efficient containment of outbreaks in epidemiological settings where the risk of spillover at the human, animal and vector interfaces is high.

Given the potential for viral evolution, coupled with climate change and population movements, new hotspots for vector‐borne and zoonotic viral diseases may emerge. Continuous surveillance and monitoring of RVF in Africa through One Health Approach is of utmost importance. Previous reviews and systematic reviews on RVF have focused on risk factors associated with RVFV circulation and transmission (Esser et al., 2019; Nicholas et al., 2014), RVFV transmission dynamics (Danzetta et al., 2016) and RVF general epidemiology (Clements et al., 2007; Gerken et al., 2022; Nanyingi et al., 2015). In this study, the burden of RVF in humans and animals in Africa was reviewed. A focus was also laid on mosquito species supporting RVFV transmission. Findings from this review and meta‐analysis contribute to our up‐to‐date understanding of the distribution and burden of RVF. These data might assist in optimal decision‐making about future public health interventions, resources allocation and operational research topics aimed at preventing and controlling RVF in Africa.

## 2 METHODS

### 2.1 Study design

The present systematic review and meta‐analysis was performed according to the Preferred Reporting Items for Systematic Reviews and Meta‐Analyses (PRISMA) guidelines (Table S1; Moher et al., 2010). The study protocol was registered in advance in PROSPERO under the number CRD42021235776.

### 2.2 Inclusion criteria

The studies included in this review were published from January 2000 to June 2022 and they met the following criteria: (i) observational or interventional (clinical trials) studies performed in Africa, (ii) publication language in English and/or French and (iii) studies including laboratory test results. Depending on the RVFV diagnostic target detected, infections were categorised into current infections (detection of live virus, viral antigen or RNA), recent infection (detection of IgM) or past infection (detection of antibodies or IgG). There were no restrictions on the type of sample or detection assay used to find RVFV. We considered studies with cross‐sectional data (one or more diagnostic targets detected) to estimate RVF prevalence of current, recent or past RVFV infections in humans and animals. We considered studies with cross‐sectional data (one or more targets) to estimate RVF CFR in humans. In the case of duplicate publications, studies with the most recent results or those providing the most data from the same study population were included after checking the authors’ name and affiliation, period of recruitment and population source of participants.

### 2.3 Exclusion criteria

Publications excluded from this study were: (i) reviews, systematic reviews and meta‐analyses, case reports and comments, (ii) studies reporting not about RVF prevalence, and CFR, or those whose RVF prevalence and CFR data could not be extracted, (iii) studies with experimental RVFV infection in animal or in vitro studies and (iv) studies not conducted in Africa. All studies with no report of laboratory‐confirmed prevalence or CFR data were also excluded.

### 2.4 Search strategy

A comprehensive literature search to identify all relevant studies was conducted on PubMed, Web of Science, African Journal Online and African Index Medicus databases using a sensitive search strategy with various combinations of the main keywords with Boolean operators ‘OR’ and ‘AND’ (Table S2). In addition to this search strategy, all relevant articles referenced by each included study were hand‐searched to ensure that all eligible studies were retrieved and included.

### 2.5 Study selection

Relevant studies identified through the initial search strategy were checked to eliminate duplicates using EndNote software. Then, two investigators (JTEB and SK) independently scrutinized, all articles one by one based on the titles and abstracts for study selection. The remaining articles were reviewed by all authors, based on full text and were selected based on the eligibility criteria outlined above. Disagreements were resolved by discussions resulting to final consensus among authors.

### 2.6 Data extraction

We used the Google Form questionnaire to record data independently extracted by the 24 authors who participated in the data extraction process from the included articles. The extracted data included the first author's name, date of publication, participant recruitment period, study design, sampling method, number of study sites, timing of data collection, country, UNSD region, country income level, study population (humans, animals and mosquitoes), patient demographic details (sex, age and recruitment place), hospitalization status, RVF case definitions (clinical case, suspected case, probable case and confirmed case), risk of bias assessment, detection assay, target detected, type of sample used, number of samples tested for RVFV, number of samples positive for RVFV and number of deaths among RVFV‐positive cases. For studies with less than 10 individual animals or with mosquitoes, we collected the names of positive animal or mosquito species for qualitative analysis. Any disagreements regarding eligibility and data collected were resolved by discussions and final consensus among authors.

### 2.7 Definitions

An RVF‐confirmed case was defined in this study as an individual whose laboratory tests were positive for anti‐RVFV‐specific antibodies, RVFV antigens or RVFV RNA. The prevalence/seroprevalence was defined as the ratio of the number of people confirmed positive for RVFV to the total number of people tested. The CFR was defined as the ratio of the number of RVFV‐positive individuals who died to the total number of RVFV‐positive individuals.

### 2.8 Appraisal of the methodological quality of included studies and risk of bias

The quality of the studies was evaluated using the tool developed by Hoy et al (Hoy et al., 2012). This tool consists of 10 items that evaluate the internal and external validity of prevalence studies. For each item, a score of 1 was given to a ‘yes’ answer, and a score of 0 was assigned to other answers, including ‘no’, ‘unclear’ and ‘not applicable’. As a result, a study was considered to be at low, moderate and high risk of bias if the total score was 7–10, 4–6 and 0–3, respectively.

### 2.9 Data synthesis and analysis

Data were analysed using R software version 4.1.0. The random effect model was performed to pool estimate of RVF prevalence in humans and animals. Pooled estimates of RVF prevalence and/or CFR in humans, as well as RVF prevalence in animals are depicted as forest plot diagrams with their corresponding 95% confidence intervals (CI). The *I* ^2^ statistical test was used to examine the magnitude of heterogeneity between the included studies, and an *I* ^2^ value greater than 75% was considered a significant heterogeneity among the studies (Borenstein et al., 2009; Higgins, 2003). Potential sources of heterogeneity were investigated by subgroup and sensitivity analysis. Publication bias was evaluated by a funnel plot and Egger's test (Egger et al., 1997).

## 3 RESULTS

### 3.1 Results of the study search

**FIGURE 1:**
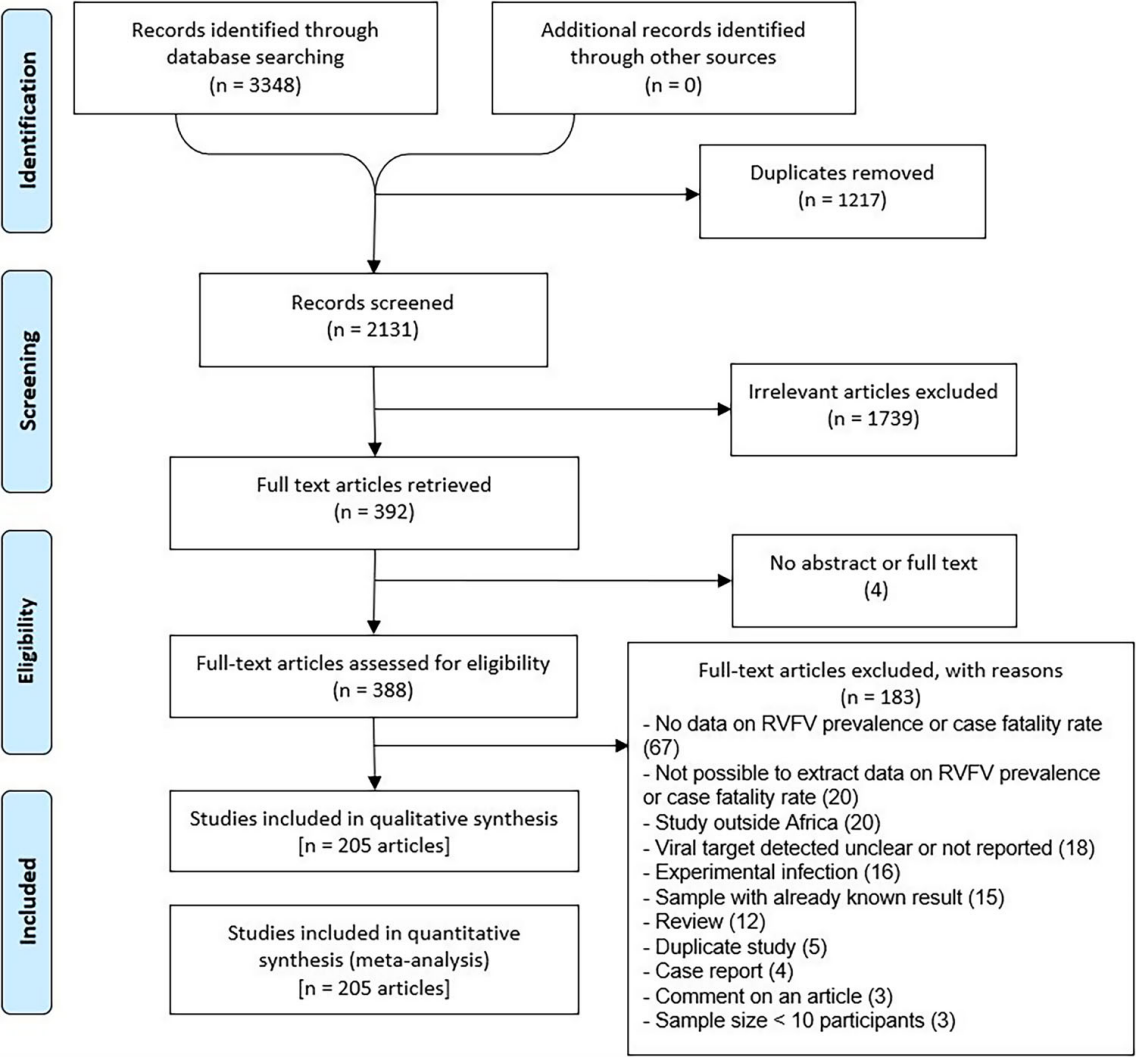
Preferred Reporting Items for Systematic Reviews and Meta‐Analyses (PRISMA) flow diagram.

As depicted in the flow chart of the article selection process presented in Figure 1, a total of 3348 articles were retrieved from databases (PubMed: 1429, Web of Science: 1868, Africa Index Medicus: 10 and African Journal Online: 41). After eliminating 1217 duplicate articles and excluding 1743 irrelevant articles based on a thorough screening of titles and abstracts, 388 articles were identified and screened for eligibility. Of the 388 eligible articles, 183 were excluded for multiple reasons described in Figure 1. Finally, a total of 205 articles (629 data on prevalence and/or CFR) met the inclusion criteria for this systematic review (Abakar et al., 2014; Abdallah et al., 2016; Adamu, 2020; Adamu et al., 2021; Adesiyun et al., 2020; Ahmed et al., 2020, 2018; Alhaji, 2020; Andayi et al., 2014; Andriamandimby et al., 2010; Andriamandimby et al., 2018; Aradaib et al., 2013; Archer et al., 2013, 2011; Atuman et al., 2022; Ayari‐Fakhfakh et al., 2011; Ba et al., 2012; Baudin et al., 2016; Beechler et al., 2015; van den Bergh et al., 2022; Bett et al., 2019; Bird et al., 2008; Bisimwa et al., 2016; Bloland et al., 2010; Blomstrom et al., 2016; Bob et al., 2017, 2022; Bonney et al., 2013; Bosworth et al., 2016; Boushab et al., 2016, 2015; Boussini et al., 2013, 2014; Breiman et al., 2010; Budasha et al., 2018; Budodo et al., 2020; Bukbuk et al., 2014; Centers for Disease Control and Prevention 2007; Chambaro et al., 2022; Chengula et al., 2014; Chevalier et al., 2005, 2011; Cichon et al., 2021; Clements et al., 2019; Cook et al., 2017; Cosseddu et al., 2021; Di Nardo et al., 2014; Diallo et al., 2005; Dione et al., 2022; Dondona et al., 2016; Durand et al., 2020, 2003; Dutuze et al., 2020; Ebogo‐Belobo et al., 2022; Eckstein et al., 2022; El Bahgy et al., 2018; El Mamy et al., 2014, 2011; El‐Harrak et al., 2011; Endale et al., 2021; Enem et al., 2020; Evans et al., 2008; Fafetine et al., 2013, 2012, 2016; Fagbo et al., 2014; Faye et al., 2014, 2007; Fischer‐Tenhagen et al., 2000; Fokam et al., 2010; Georges, 2018; Gora et al., 2000; Gray et al., 2015; Grolla et al., 2012; Grossi‐Soyster et al., 2017; Gudo, Lesko, et al., 2016; Gudo, Pinto, et al., 2016; Guillebaud et al., 2018; Halawi et al., 2019; Hama et al., 2019; Hanafi et al., 2011; Hassan et al., 2020; Hassanain et al., 2010; Hassine et al., 2017; Heinrich et al., 2012; Horton et al., 2014; Ibrahim et al., 2021; Jäckel, Eiden, El Mamy, et al., 2013; Jeanmaire et al., 2011; Jori et al., 2015; Kading et al., 2018; Kanouté et al., 2017; Kifaro et al., 2014; Labeaud et al., 2008, 2015; Labeaud, Cross, et al., 2011; Muiruri et al., 2015; Labeaud, Muiruri, et al., 2011; Labeaud Sutherland, et al., 2011; Lagare et al., 2019; Lagerqvist et al., 2013; LeBreton et al., 2006; Lubisi et al., 2020; Lutomiah et al., 2014; Lwande et al., 2015; Lysholm et al., 2022; Macharia et al., 2010; Maganga et al., 2017; Magona et al., 2013; Mahmoud & Ali, 2021; Mahmoud et al., 2018; Makiala‐Mandanda et al., 2018; Mapaco et al., 2012; Marrama et al., 2005; Matiko et al., 2018; Mbotha et al., 2018; Mease et al., 2011; Mhina et al., 2015; Miller et al., 2011; Mohamed et al., 2019; Moiane et al., 2017; Monaco et al., 2013; Mordi et al., 2020; Mroz et al., 2017a; Mroz et al., 2017b; Msimang et al., 2019; Nabeth, 2001; Nakouné et al., 2016; Nakounne et al., 2001; Nanyingi et al., 2017; Ndengu et al., 2020; Ndiana et al., 2019; Ndiaye et al., 2018; Ngoshe et al., 2020; Njenga et al., 2010; Njenga et al., 2009; O'hearn et al., 2016; Ochieng et al., 2015; Odaibo et al., 2019; Olive et al., 2013; Opayele, 2019; Opayele et al., 2018; Oragwa et al., 2022; Owange et al., 2014; Oyas et al., 2018; Paweska, Burt et al., 2003; Paweska, Burt, et al., 2005; Paweska et al., 2007; Paweska, Mortimer, et al., 2005; Paweska, Smith, et al., 2003; Paweska et al., 2008; Pawęska et al., 2021; Peterson et al., 2017; Poueme et al., 2019; Pourrut et al., 2010; Centers for Disease Control and Prevention, 2007; Rakotoarivelo et al., 2011; Ratovonjato et al., 2011; Ringot et al., 2004; Rissmann, Eiden, El Mamy, et al., 2017; Rissmann, Eiden, Wade, et al., 2017; Roger, 2011; Roger et al., 2014; Rostal et al., 2010; Roug et al., 2020; Rugarabamu et al., 2022; Sadeuh‐Mba et al., 2018; Salekwa et al., 2019; Sall et al., 2000; Sanderson et al., 2020; Nguku et al., 2010; Sang et al., 2010; Schoepp et al., 2014; Schwarz et al., 2012; Selmi et al., 2020; Sindato et al., 2015; Sindato et al., 2013; Soumare et al., 2007; Sow, Faye, Ba, et al., 2014; Sow, Faye, et al., 2016; Sow, Faye, Faye, et al., 2014; Sow, Loucoubar, et al., 2016; Spiropoulou & Nyakarahuka, 2018; Sternberg Lewerin, 2018; Stoek et al., 2022; Sumaye et al., 2015, 2013; Swai & Schoonman, 2009; Swai & Sindato, 2015; Tigoi, 2020; Tigoi et al., 2015; Traoré‐Lamizana et al., 2001; Troupin et al., 2022; Tshilenge et al., 2019; Umuhoza et al., 2017; Ushijima et al., 2021; van den Bergh et al., 2020; Wensman et al., 2015; Wolff et al., 2018; Woods et al., 2002; Youssef, 2001, 2009; Youssef & Donia, 2001, 2002; Zouaghi et al., 2021).

### 3.2 Characteristics of the included studies

The main characteristics of the included studies are presented in Table S3. These studies were published between 2000 and 2022, whereas their participant’ were recruited between 1974 and 2020. The included studies performed investigation in humans (142 of 629 data), mosquitoes (125 of 629 data) and other animal species (362 of 629 data). Most of the 629 estimated data were prospectively collected (93.5%; 588/629), from cross‐sectional study surveys (87.5%; 539/629), using a nonprobability sampling technique (71.4%; 449/628). Countries in East Africa (49.3%; 310/409) and West Africa (27.0%; 170/629) in the UNSD region had the largest number of data estimates. Kenya (20.2%; 127/629), Mauritania (15.6%; 98/629) and Tanzania (9.7%; 61/629) were, respectively, the countries with highest number of studies considered. The predominant diagnostic technique used for laboratory detection of RVFV infections was indirect ELISA (57.2%; 360/629), and RVFV‐specific IgG antibodies were the most frequently detected diagnostic target (37.5%; 236/629). Most studies included had a moderate risk of bias (74.4%; 468/629) (Table S4).

### 3.3 Host species distribution of RVFV

**FIGURE 2:**
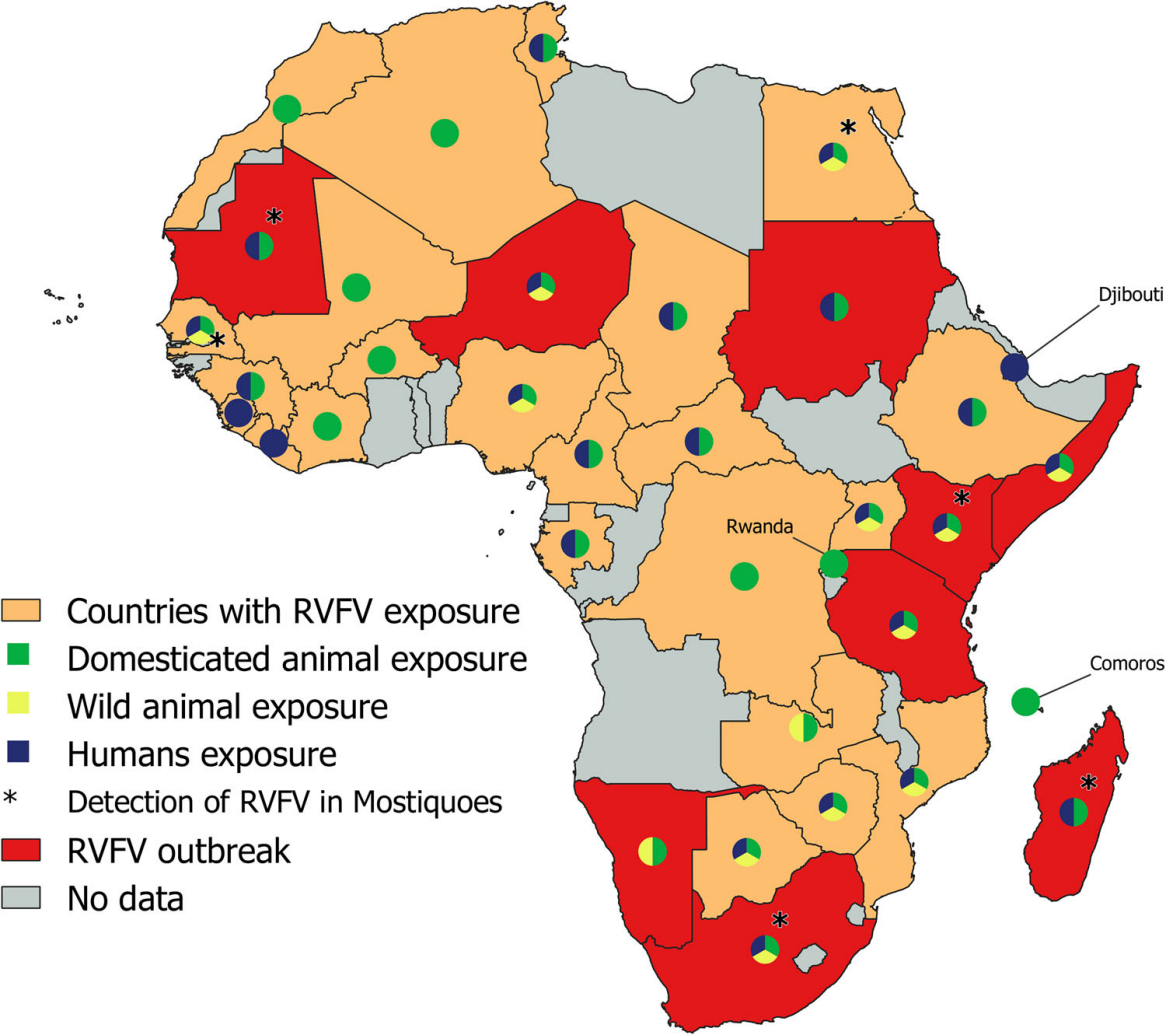
Map of the Rift Valley fever virus (RVFV) spatial distribution. The map was taken from https://www.naturalearthdata.com and modified with QGIS software version 3.16.0‐Hannover.

The biological evidence of RVFV based on laboratory results was reported in 36 African countries (Figure 2). RVF‐associated outbreaks in human and/or animal populations were reported in nine countries, and the highest number of outbreaks occurred in Mauritania, respectively, in 1998, 2003, 2010, 2012 and 2015. Of the countries which reported RVFV exposure, based either on serological evidence or direct virus detection, eight countries only reported domestic animal exposures, whereas three only reported human exposures. Of the 14 countries that reported exposure in wildlife animals, 12 also reported exposures in both humans and domestic animals. This host species distribution of RVFV infections shows that, apart from Namibia, all African countries that faced an RVF outbreak reported RVFV exposure in domestic animals (Figure 2). Only two studies carried out in Uganda and Zimbabwe reported abortion in cattle and goats (Budasha et al., 2018; Ndengu et al., 2020).

The RVF prevalence in individual mosquitoes ranged from 0.0% to 25% (Abdel‐Wahab et al., 1978; Bisimwa et al., 2016; Diallo et al., 2005; Faye et al., 2007; Hanafi et al., 2011; Lutomiah et al., 2014; Mhina et al., 2015; Nabeth, 2001; Ratovonjato et al., 2011; Roger et al., 2014; Sang et al., 2010; Sow, Faye, Faye, et al., 2014). RVFV‐infected mosquitoes belonged to the Culicidae family and were identified in six countries (Senegal, Mauritania, Egypt, Kenya, Madagascar and South Africa). The mosquito genera that were positive for RVFV included *Aedes* (*Aedes mcintoshi/circumluteolus*, *Aedes pembaensis*, *Aedes ochraceus*, *Aedes fowleri*, *Aedes vexans* and *Aedes* spp.), *Anopheles* (*Anopheles pharoensis*, *Anopheles squamosus*, *Anopheles* spp. and *Anopheles coustani)*, *Culex* (*Culex poicilipes*, *Culex univittatus*, *Culex quinquefasciatus*, *Culex bitaeniorhynchus*, *Culex antennatus* and *Culex* spp.), *Mansonia* (*Mansonia africana*, *Mansonia uniformis* and *Mansonia* spp.) and *Hodgesia* sp. field RVFV‐infected mosquitoes were reported both during outbreaks (Ba et al., 2012; Diallo et al., 2005; Faye et al., 2014, 2007; Hanafi et al., 2011; Labeaud, Sutherland, et al., 2011; Lutomiah et al., 2014; Sall et al., 2000; Sang et al., 2010; Sow, Faye, et al., 2016; Traoré‐Lamizana et al., 2001; Youssef, 2001) and during interepizootic periods (Ndiaye et al., 2018; Ratovonjato et al., 2011; Figure 2).

### 3.4 RVF CFR among humans in Africa

**FIGURE 3:**
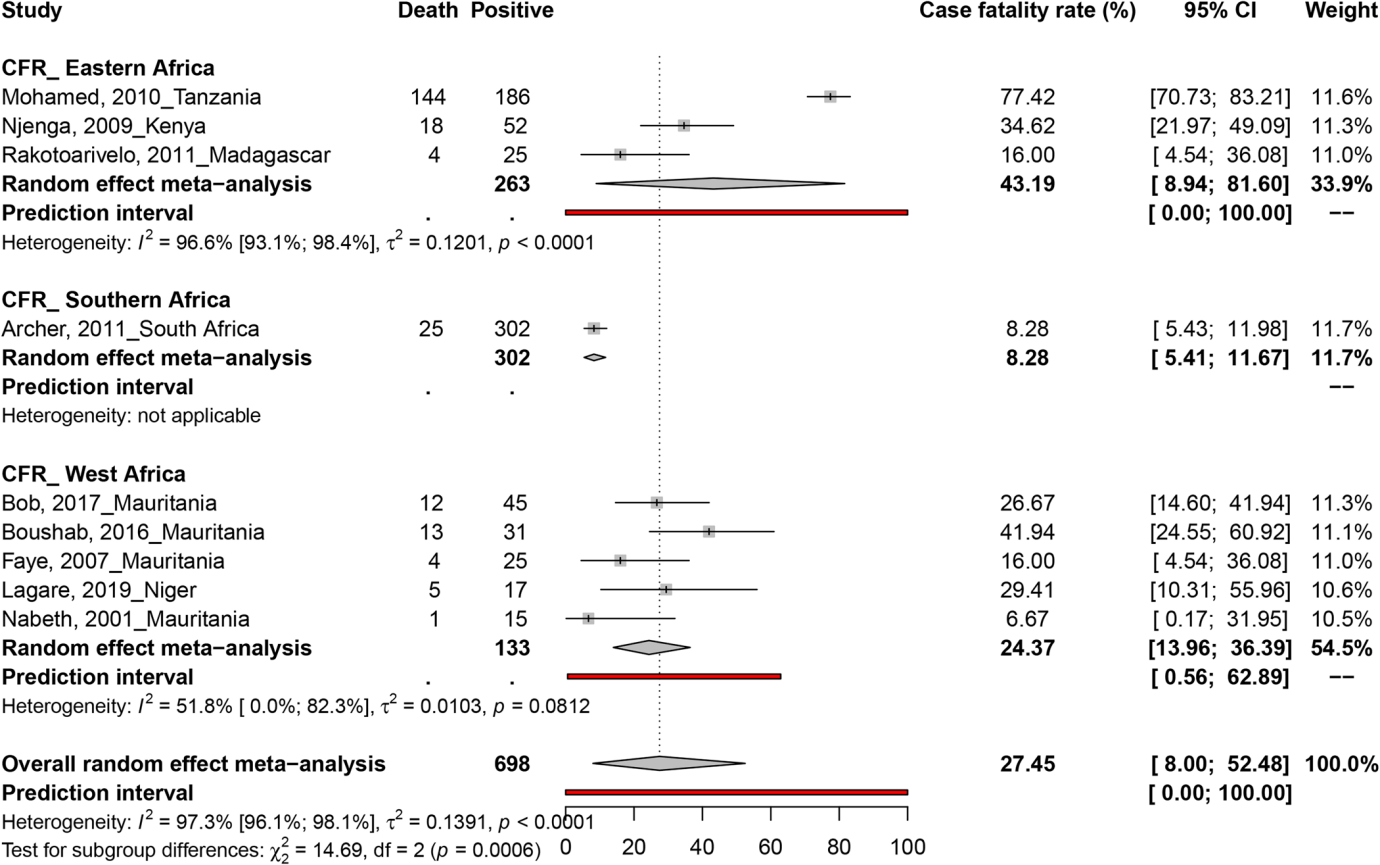
Case fatality rate (CFR) estimates of Rift Valley fever virus (RVFV) infections among humans in Africa.

The CFR due to RVFV infection in humans was reported in six countries during the outbreak periods in Kenya (Njenga et al., 2009), Madagascar (Rakotoarivelo et al., 2011), Mauritania (Bob et al., 2017; Boushab et al., 2016; Faye et al., 2007; Nabeth, 2001), Niger (Lagare et al., 2019), Tanzania (Bloland et al., 2010) and South Africa (Archer et al., 2011) (Figures 2 and 3). The pooled RVF CFR was estimated to be 27.5% [95% CI = 8.0–52.5] based on sample of 698 participants recruited in 9 studies. Data on CFR featured presented substantial heterogeneity (*I* ^2^ = 97.3% [95% CI = 96.1–98.1], *p* < 0.0001) (Archer et al., 2011; Bloland et al., 2010; Bob et al., 2017; Boushab et al., 2016; Faye et al., 2007; Lagare et al., 2019; Nabeth, 2001; Njenga et al., 2009; Rakotoarivelo et al., 2011) (Figure 3 and Table 1). As supported by the funnel plot (Figure S1), and Egger's regression test (*p* = 0.975), there was no evidence of potential publication bias on CFR data.

**TABLE 1:** Summary of meta‐analysis results for case fatality rate, and prevalence, of Rift Valley fever (RVF) virus in humans, and other animal species.

	Prevalence (%) (95% CI)	95% prediction interval	*N* studies	*N* participants	^a^ *H* (95% CI)	^b^ *I* ^2^ (95% CI)	*p* heterogeneity	*p* Egger test
RVF case fatality rate in humans								
Overall	27.5 [8–52.5]	[0–100]	9	698	6 [5.1–7.2]	97.3 [96.1–98.1]	<0.001	0.318
Cross‐sectional	27.8 [20.1–36.1]	[10.8–48.5]	6	195	1.2 [1–1.9]	32 [0–72.5]	0.196	0.318
Low risk of bias	22.4 [9–39.3]	[0–84.8]	5	420	2.8 [1.9–4.1]	86.9 [71.8–93.9]	<0.001	0.949
RVF prevalence in humans								
Overall	7.9 [6.3–9.8]	[0–37]	133	102,979	10 [9.7–10.4]	99 [98.9–99.1]	<0.001	0.001
Cross‐sectional	5.9 [4.5–7.6]	[0–30.7]	111	95,719	9.8 [9.4–10.1]	99 [98.9–99]	<0.001	0.008
Low risk of bias	8 [6–10.2]	[0–36.3]	89	86,990	10.9 [10.5–11.3]	99.2 [99.1–99.2]	<0.001	0.001
RVF prevalence in animals								
Overall	9.4 [8.2–10.7]	[0–40.5]	349	136,206	7.4 [7.3–7.6]	98.2 [98.1–98.3]	<0.001	0.94
Cross‐sectional	9.9 [8.6–11.3]	[0–41.9]	318	128,335	7.7 [7.5–7.9]	98.3 [98.2–98.4]	<0.001	0.943
Low risk of bias	7.7 [5.6–10]	[0–31.4]	63	29,262	6.4 [6.1–6.8]	97.6 [97.3–97.9]	<0.001	

Abbreviations: CI, confidence interval; N, number; NA, not applicable.

^a^ *H* Is a measure of the extent of heterogeneity, a value of *H* = 1 indicates homogeneity of effects and a value of *H* >1 indicates a potential heterogeneity of effects.

^b^ *I* ^2^ describes the proportion of total variation in study estimates that is due to heterogeneity, a value >50% indicates the presence of heterogeneity.

### 3.5 Prevalence of RVFV infection among humans in Africa

**FIGURE 4:**
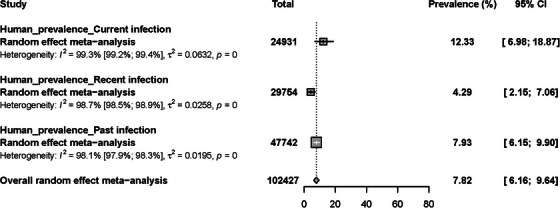
Prevalence estimates of Rift Valley fever virus infections among humans in Africa.

The pooled RVF prevalence in humans was estimated to be 7.8% [95% CI = 6.2–9.6] based on a sample of 102,427 participants. RVF prevalence data displayed presented substantial heterogeneity among the studies (*I* ^2^ = 99.0% [95% CI = 98.9–99.0], *p* < 0.0001) (Adamu et al., 2021; Ahmed et al., 2020, 2018; Andayi et al., 2014; Andriamandimby et al., 2010; Aradaib et al., 2013; Archer et al., 2013, 2011; Atuman et al., 2022; Baudin et al., 2016; van den Bergh et al., 2022; Bett et al., 2019; Bloland et al., 2010; Bob et al., 2017, 2022; Bonney et al., 2013; Bosworth et al., 2016; Boushab et al., 2015; Breiman et al., 2010; Budodo et al., 2020; Bukbuk et al., 2014; Chambaro et al., 2022; Cichon et al., 2021; Clements et al., 2019; Cook et al., 2017; Cosseddu et al., 2021; Dione et al., 2022; Durand et al., 2003; Eckstein et al., 2022; Enem et al., 2020; Faye et al., 2007; Fokam et al., 2010; Gray et al., 2015; Grolla et al., 2012; Grossi‐Soyster et al., 2017; Gudo, Lesko, et al., 2016; Gudo, Pinto, et al., 2016; Guillebaud et al., 2018; Hassan et al., 2020; Hassanain et al., 2010; Heinrich et al., 2012; Ibrahim et al., 2021; Labeaud et al., 2008; Labeaud, Muiruri, et al., 2011; Labeaud et al., 2015; Muiruri et al., 2015; Lagare et al., 2019; Lysholm et al., 2022; Macharia et al., 2010; Mahmoud & Ali, 2021; Makiala‐Mandanda et al., 2018; Marrama et al., 2005; Mease et al., 2011; Mohamed et al., 2019; Msimang et al., 2019; Nabeth, 2001; Nakouné et al., 2016; Nakounne et al., 2001; Njenga et al., 2010; O'hearn et al., 2016; Ochieng et al., 2015; Opayele et al., 2018; Oragwa et al., 2022; Paweska, Burt, et al., 2005; Paweska et al., 2007; Paweska, Mortimer, et al., 2005; Pawęska et al., 2021; Pourrut et al., 2010; Rugarabamu et al., 2022; Sadeuh‐Mba et al., 2018; Sanderson et al., 2020; Nguku et al., 2010; Schoepp et al., 2014; Schwarz et al., 2012; Sow, Faye, Ba, et al., 2014; Sow, Faye, et al., 2016; Sow, Faye, Faye, et al., 2014; Sow, Loucoubar, et al., 2016; Spiropoulou & Nyakarahuka, 2018; Stoek et al., 2022; Sumaye et al., 2015; Swai & Schoonman, 2009; Tigoi, 2020; Tigoi et al., 2015; Troupin et al., 2022; Ushijima et al., 2021; Weber et al., 2018; Wolff et al., 2018; Woods et al., 2002; Youssef, 2009; Zouaghi et al., 2021). RVF prevalence estimates were found to be 12.3% [95% CI = 7–18.9; 24,931 participants] in people with current infection, 4.3% [95% CI = 2.2–7.1; 29,754 participants] in people with recent infection and 7.9% [95% CI = 6.2–9.9; 47,742 participants] in people with past infection (Table 1, Figure 4 and Figure S2). Both funnel plot (Figure S3), and Egger's regression test (*p* < 0.01) results indicated potential publication bias in prevalence data.

### 3.6 Prevalence of RVFV infection in other animal species in Africa

**FIGURE 5:**
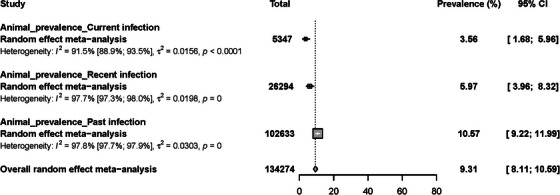
Prevalence estimates of Rift Valley fever virus infections in other animal species in Africa.

Evidence of RVFV infection was documented in other animals of the orders *Artiodactyla*, *Proboscidea*, *Perissodactyla*, *Rodentia* and *Chiroptera*. The pooled RVF prevalence in animal species was estimated to be 9.3% [95% CI = 8.1–10.6] in an overall sample of 134,274 individual animals. RVF prevalence data in animals featured presented substantial heterogeneity among studies (*I* ^2^ = 98.2% [95% CI = 98.1–98.3], *p* < 0.0001) (Abakar et al., 2014; Abdallah et al., 2016; Adamu, 2020; Adesiyun et al., 2020; Alhaji, 2020; Andriamandimby et al., 2018; Ayari‐Fakhfakh et al., 2011; Beechler et al., 2015; Bett et al., 2019; Bird et al., 2008; Blomström et al., 2016; Boussini et al., 2014; Budasha et al., 2018; Chengula et al., 2014; Chevalier et al., 2005, 2011; Di Nardo et al., 2014; Dondona et al., 2016; Durand et al., 2020; Dutuze et al., 2020; Ebogo‐Belobo et al., 2022; El Bahgy et al., 2018; El Mamy et al., 2011; El‐Harrak et al., 2011; Endale et al., 2021; Evans et al., 2008; Fafetine et al., 2012, 2013; Fagbo et al., 2014; Faye et al., 2007; Fischer‐Tenhagen et al., 2000; Georges, 2018; LaBeaud, Cross, et al., 2011; Gora et al., 2000; Halawi et al., 2019; Hanafi et al., 2011; Hassan et al., 2020; Hassine et al., 2017; Horton et al., 2014; Ibrahim et al., 2021; Jäckel, Eiden, Balkema‐Buschmann, et al., 2013; Jäckel, Eiden, El Mamy, et al., 2013; Jeanmaire et al., 2011; Jori et al., 2015; Kading et al., 2018; Kanouté et al., 2017; Kifaro et al., 2014; Lagare et al., 2019; Lagerqvist et al., 2013; LeBreton et al., 2006; Lubisi et al., 2020; Lwande et al., 2015; Maganga et al., 2017; Magona et al., 2013; Mahmoud et al., 2018; Mapaco et al., 2012; Matiko et al., 2018; Mbotha et al., 2018; Miller et al., 2011; Moiane et al., 2017; Mordi et al., 2020; Mroz et al., 2017a; Mroz et al., 2017b; Munyua et al., 2016; Nabeth, 2001; Nakouné et al., 2016; Nanyingi et al., 2017; Ndengu et al., 2020; Ndiana et al., 2019; Ngoshe et al., 2020; Odaibo et al., 2019; Olive et al., 2013; Opayele, 2019; Owange et al., 2014; Oyas et al., 2018; Paweska, Burt, et al., 2003; Paweska, Burt, et al., 2005; Paweska, Mortimer, et al., 2005; Paweska, Smith, et al., 2003; Paweska et al., 2008; Peterson et al., 2017; Poueme et al., 2019; Ringot et al., 2004; Rissmann, Eiden, El Mamy, et al., 2017; Rissmann, Eiden, Wade, et al., 2017; Roger, 2011; Roger et al., 2014; Rostal et al., 2010; Roug et al., 2020; Salekwa et al., 2019; Selmi et al., 2020; Sindato et al., 2015; Sindato et al., 2014; Soumare et al., 2007; Sow, Faye, et al., 2016; Spiropoulou & Nyakarahuka, 2018; Sternberg Lewerin, 2018; Sumaye et al., 2013; Swai & Sindato, 2015; Tshilenge et al., 2019; Umuhoza et al., 2017; van den Bergh et al., 2020; Wensman et al., 2015; Youssef, 2009; Youssef & Donia, 2001). The prevalence estimates of RVFV infection were, respectively, at 3.6% [95% CI = 1.7–6.0], 6.0% [95% CI = 4.0–8.3] and 10.6% [95% CI = 9.2–12.0] in animals with ongoing infection, recent infection and past infection (Table 1, Figure 5 and Figure S4). Based on the funnel plot (Figure S5) and Egger's regression test (*p* < 0.01), there was potential publication bias of the RVF prevalence estimates. Qualitative analysis showed that the animal species that were positive for RVFV in studies with less than 10 animals included African elephant, black‐faced impala, black rhino, cow, sheep, giraffe, goat, camel and cattle (Bird et al., 2008; Breiman et al., 2010; Dondona et al., 2016; Lagare et al., 2019; Lwande et al., 2015; Monaco et al., 2013; Nabeth, 2001; Sindato et al., 2013).

### 3.7 Subgroup analyses

**FIGURE 6:**
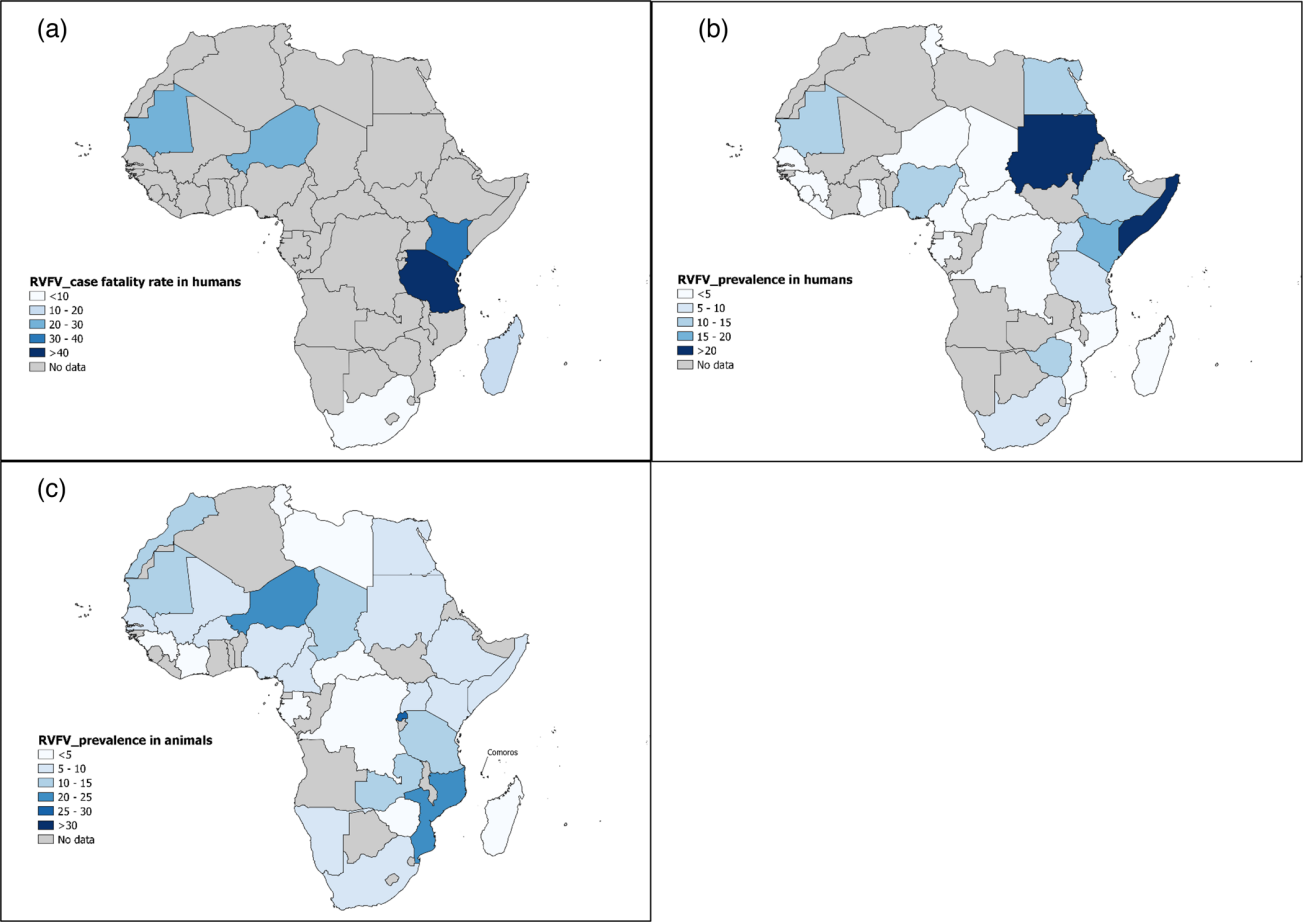
Case fatality rate and prevalence/seroprevalence estimates of Rift Valley fever virus (RVFV) infections in humans, and other animal species in Africa. Part (a) shows the case fatality rate in humans. Parts (b and c) show RVFV infection in humans and animals, respectively. *Source*: The base map was retrieved from https://www.naturalearthdata.com and modified with QGIS software version 3.16.0‐Hannover.

This systematic review and meta‐analysis investigated subgroup analysis of RFV CFR and prevalence in humans and other animal species, and the results are summarized in Tables S5–S7. RVF CFR varied with respect to location: 43.2% [95% CI = 8.9–81.6] in Eastern Africa and 24.4% [95% CI = 14.0; 36.4] in West Africa (Figure 6a).

The subgroup analyses indicated that RVF prevalence in humans showed, significantly higher prevalence in community outbreak studies (22.2%; 95% CI = 15.5–29.7; *p* < 0.001), in probabilistic sampling studies (11.9%; 95% CI = 8.0–16.4; *p* = 0.029), in Sudan (48.9%; 95% CI = 10.1–88.5; *p* < 0.001), in the Northern Africa UNSD region (20.0%; 95% CI = 3.0–46.0; *p* < 0.001) (Figure 6b), in lower‐middle‐income economy countries (9.3%, 95% CI = 6.8–12.0; *p* = 0.014), in suspected RVF cases (12.8%, 95% CI = 8.9–17.3; *p* = 0.003) and in peoples developing current infections (12.3%; 95% CI = 7.0–18.9; *p* < 0.001).

Data analysis in other animal species showed that RVF prevalence was significantly higher in cross‐sectional studies (9.9%, 95% CI = 8.6–11.3; *p* < 0.001), which was carried out in Southern Africa (10.9%, 95% CI = 6.3–16.6), Eastern Africa (10.6%, CI = 8.6–12.6) and West Africa (10.1%, 95% CI = 7.3–13.2; *p* = 0.013). Prevalence in other animal species was particularly higher in Comoros (30.9%, 95% CI = 27.3–34.6), Rwanda (26.4%, 95% CI = 14.8–39.8), Mozambique (22.6%, 95% CI = 15.3–31), Niger (21.1%, 95% CI = 8.7–36.8), and Zambia (19.6%, 95% = 5.6–38.5; *p* < 0.001) (Figure 6c). Highest prevalence estimates were found in animals from the Artiodactyla family (10.2%, 95% CI = 8.9–11.6; *p* < 0.001), domestic animals (10.4%, 95% CI = 9.0–12.0; *p* = 0.012) and animals with past infection (10.7%, 95% CI = 9.4–12.1; *p* < 0.001).

## 4 DISCUSSION

This systematic review and meta‐analysis gathering data from human, animal and vector interfaces in a One Health approach provides a substantial update of the global epidemiology of RVFV infection in Africa. It summarizes data from articles published between 2000 and 2022 in diverse epidemiological settings in Africa with the recruitment of participants spanning nearly 45 years; from 1974 to 2020. To the best of our knowledge, this is the first systematic review and meta‐analysis to concurrently summarises data about the overall CFR and prevalence of RVF in humans and animals in Africa. This study revealed an RVF CFR of 27.5% and an overall RVF prevalence of 7.8% and 9.3% in humans and animals, respectively.

Historically, it is known that most human cases of RVF are non‐severe or asymptomatic with a CFR approximately 1%–2% of infected patients with or without biological confirmation (Bird et al., 2009; Ikegami & Makino, 2011; Wright et al., 2019). However, RVF CFR can be up to 50% among patients with the severe form of the disease (Madani et al., 2003). Although the 1977 outbreak in Egypt was one of the largest human outbreaks, with nearly 200,000 suspected cases and approximately 600 deaths (Fawzy & Helmy, 2019; Meegan et al., 1979), it is not certain that all cases were associated to RVFV infection in the absence of laboratory confirmation. Indeed, RVF shares signs and symptoms with other endemic diseases that are prevalent in Africa. For example, in the 1998 outbreak in Mauritania, not all suspected cases with haemorrhagic fevers were confirmed in the laboratory; thus, suggesting that the haemorrhage observed might be related to other causes (Nabeth, 2001). This finding was consistent with another report based on pathological evaluation of post‐mortem and necropsy tissue samples from animals and humans clinically suspected of having RVFV infection during the 2006–2007 outbreak in Eastern Africa (Breiman et al., 2010). In this review, relatively high estimates of the RVF CFR in humans (27.5%) during outbreak periods were consistent with previous study among confirmed cases (Anywaine et al., 2022). This high RVF CFR could be explained by the fact that during outbreaks, epidemiological investigations focus primarily on the identification of severe cases in epidemiological settings where all RVF cases are hard to trace. In such setting, asymptomatic or uncomplicated cases would usually stay at home and would likely be poorly investigated or detected. In the other hand, this high CFR could also be explained by the absence of a specific treatment, lack of supportive care facilities and delay between disease onset and hospitalisation due to reluctance in seeking medical care on time especially in resource‐limited settings. In such conditions, the vital prognosis of patients is already poor, thus increasing the RVF CFR. However, in some studies reporting RVF CFR, many people who died had at least one complication that appeared to be the mains causes of human mortality. These complications include particularly haemorrhagic syndrome (Adam et al., 2010; Al‐Hazmi et al., 2005, 2003; Boushab et al., 2016, 2015; Madani et al., 2003; Rakotoarivelo et al., 2011) but also acute hepatic or renal failure (Anywaine et al., 2022).

The overall RVF prevalence levels obtained in this review are similar to those previously reported by Clark and colleagues (Clark et al., 2018). They reported an RVF prevalence of 5.9% in humans, and 8.8%–12.9% in animals (livestock and wildlife). Both studies report evidence of RVFV circulation in a wide range of animals with most RVFV host species belonging to *Artiodactyla* order. Interestingly, the high prevalence of RVFV infections in wildlife animals (*Perissodactyla* and *Rodentia* order) is indicative of RVFV circulation in wildlife even though the epidemiological patterns and modalities of this circulation are still to be documented. Therefore, it is necessary to assess RVFV infection where wildlife habitat has become increasingly overlapping with that of livestock as a result of deforestation, extensive farming, hunting and bushmeat trades. More interestingly, our spatial analysis revealed that of the nine countries that have reported outbreaks, six showed an overlap between domestic and wild animals. This overlapping could likely contribute to RVFV maintenance and amplification in livestock before transmission into humans, particularly in rural settings where interfaces between wildlife and livestock are favourable to spillover transmission. Furthermore, livestock and wild ungulate are vulnerable to the same floodwater mosquitoes, thus favouring interspecies transmission during mosquito feeding without any physical contact between animal hosts living in sympatry.

RVF outbreaks usually occur after exceptional years of prolonged above‐average rainfalls, leading to a large increase in mosquito populations (Linthicum et al., 2016). High diversity of potential RVFV blood‐feed vectors belonging mainly to genera *Aedes* and *Culex* spp. has been described in the literature (Linthicum et al., 2016; Lumley et al., 2017). In this review, overall 21 individual or pooled mosquito species were identified as vectors of RVFV in the included studies (Ba et al., 2012; Diallo et al., 2005; Faye et al., 2007; Hanafi et al., 2011; Labeaud, Sutherland, et al., 2011; Lutomiah et al., 2014; Ndiaye et al., 2018; Ratovonjato et al., 2011; Sall et al., 2000; Sang et al., 2010; Sow, Faye, et al., 2016; Traoré‐Lamizana et al., 2001; Youssef, 2001). This number is lower compared to more than 50 species of RVFV transmission‐competent mosquito vectors reported in the literature. Some of these RVFV vectors were identified prior to our study inclusion period or during experimental infections and transmission in the laboratory (Linthicum et al., 2016); whereas this review considered only naturally occurring field infection studies. Favourable conditions in Africa, such as heavy rainfalls, intensive agricultural activity, wet environments and other biotic and abiotic environmental conditions, are responsible for the proliferation of these vectors (Baba et al., 2016; Linthicum et al., 2016). This situation may contribute to the increase in mosquito breeding and greater exposure of animals that will ultimately result to increased risk for RVFV maintenance in particular epidemiological settings. Some of these mosquitoes, such as *A. mcintoshi*, found among RVFV‐positive mosquitoes identified in this study, are responsible for the long‐term maintenance of RVFV in an enzootic sylvatic cycle through vertical transmission of the virus to mosquito progeny via drought‐resistant eggs (Linthicum et al., 2016; Lumley et al., 2017).

The findings from this review are of great interest for the better understanding the epidemiology of RVF in Africa. These findings are needed to rule out geographical association of RVFV‐associated threat to human and animal health. This is critical for the development, optimisation and prioritization of effective and efficient surveillance and control programmes in these target vulnerable regions. Public health authorities will also find valuable data for better preparedness of emergency response systems enabling timely containment of potential RVF‐associated outbreaks. RVF surveillance programmes in Africa should include, (i) public health education and awareness campaigns targeting people and professionals at high risk of infection, (ii) early laboratory confirmation of cases and establishment of procedures for the management of RVFV‐infected patients and (iii) search for RVFV infection as a differential diagnosis of patients with high‐grade fever, in patients living/working in close contact with animals or animal product derivative.

This study also underscores the need for further research aiming to monitor the trend of RVF prevalence in animals, humans and mosquitoes, especially in rural settings affected by agro‐ecological disturbances bringing wildlife and livestock in proximity. In that perspective, syndromic, serological and virological surveys in livestock and wildlife must be carried out periodically to assess the potentially risk for public health security. Differential diagnosis studies among patients presenting with signs and symptoms that overlap with those of RVF would be very informative for addressing misdiagnosis and underestimation of the burden of RVF in areas where direct cross‐species and vector‐borne transmissions are likely to occur. Control of mosquito breeding and entomological studies must also be carried out regularly in high‐risk areas to generate key information on RVF distribution and transmission routes.

This study has some limitations that need to be taken into consideration. There was substantial heterogeneity among the studies included and that heterogeneity persisted in subgroup analyses. Besides, some of the overall estimates were affected by significant publication bias. Lack of records did not allow us differentiate animal serological markers associated to vaccination in one hand and natural infection in the other hand. Therefore, animal‐related data must be interpreted with caution. We excluded in our analysis studies with small sample sizes (those with fewer than 10 participants), to strengthen the robustness of our analysis. Although only three studies with less than 10 participants were excluded, their results could have affected our estimates. Despite these limitations, this systematic review provides an insight into the epidemiology of RVF in diverse epidemiological settings in Africa. Moreover, the high number of articles included in this study increases the accuracy of the estimates of the studied epidemiological parameters investigated.

In conclusion, this systematic review with meta‐analysis presented data on the current state of RVFV epidemiology in diverse hosts species including humans, livestock, wildlife animal and mosquito vectors from a wide range of geographical areas in Africa. Given the high RVF CFR and subclinical circulation of RVFV in humans and animals, it is crucial to implement a One Health approach to RVF surveillance and control at humans, animals and mosquito interfaces in African countries. Tackling RVFV infections in countries at risk will strengthen the development of efficient clinical and laboratory diagnostic tools and drive forward the preparedness for rapid and efficient control of potential future outbreaks.

## AUTHOR CONTRIBUTIONS

Jean Thierry Ebogo‐Belobo, Sebastien Kenmoe and Richard Njouom were responsible for conception and design of the study as well as project administration. Jean Thierry Ebogo‐Belobo, Sebastien Kenmoe, Ngu Njei Abanda, Arnol Bowo‐Ngandji, Donatien Serge Mbaga, Jeannette Nina Magoudjou‐Pekam, Ginette Irma Kame‐Ngasse, Serges Tchatchouang, Elisabeth Zeuko'o Menkem, Etienne Atenguena Okobalemba, Efietngab Atembeh Noura, Dowbiss Meta‐Djomsi, Martin Maïdadi‐Foudi, Josiane Kenfack‐Zanguim, Raoul Kenfack‐Momo, Cyprien Kengne‐Nde, Seraphine Nkie Esemu, Wilfred Fon Mbacham, Serge Alain Sadeuh‐Mba, Lucy Ndip, Richard Njouom were responsible for the data curation and interpretation of results. Cyprien Kengne‐Nde and Sebastien Kenmoe were responsible for statistical analysis and methodology. Jean Thierry Ebogo‐Belobo and Sebastien Kenmoe wrote the original draft. All authors critically reviewed the first draft and approved the final version of the paper for submission and have read and approve the final manuscript.

## CONFLICT OF INTEREST STATEMENT

No conflict of interests is declared.

### PEER REVIEW

The peer review history for this article is available at https://publons.com/publon/10.1002/vms3.1238.

## ETHICS STATEMENT

The authors confirm that the ethical policies of the journal, as noted on the journal's author guidelines page, have been adhered to. No ethical approval was required as this is a review article with no original research data.

## Supporting information

Supporting Information

Supporting Information

Supporting Information

Supporting Information

Supporting Information

Supporting Information

## Data Availability

The data that support the findings of this study are available from the corresponding author upon reasonable request.
