# Supplementary figures and images for: Contemporary epidemiological data of Rift Valley fever virus in humans, mosquitoes and other animal species in Africa: A systematic review and meta‐analysis
Source: Vet Med Sci. 2023 Aug 7;9(5):2309–28. doi: 10.1002/vms3.1238 (PMC10508527; doi:10.1002/vms3.1238)

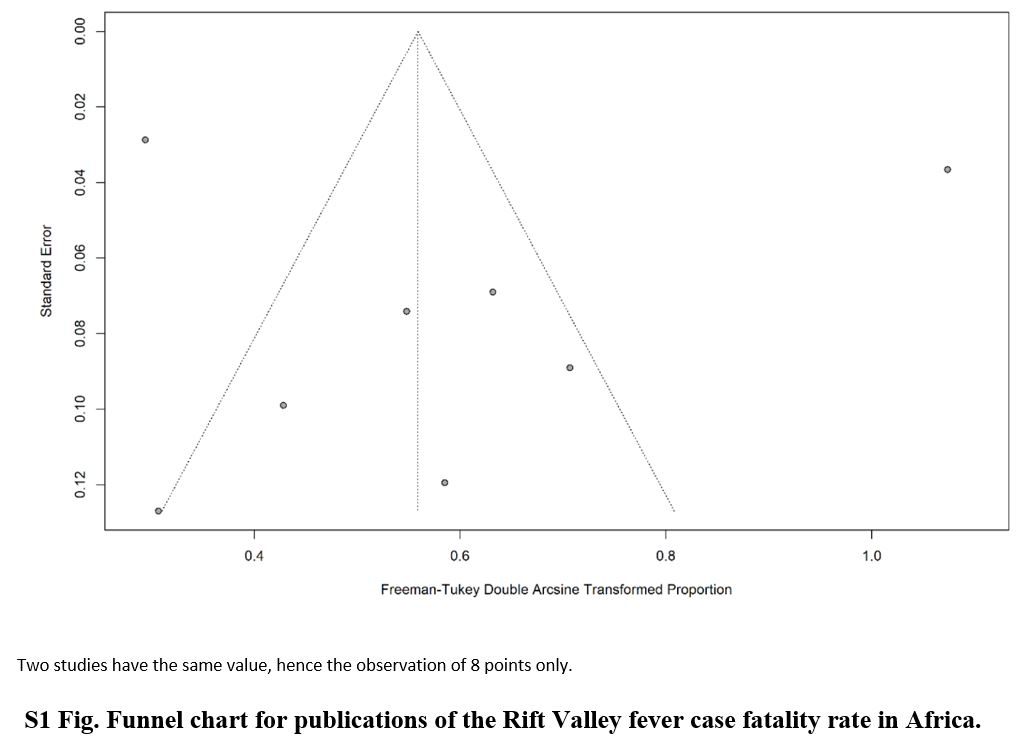

Supplement: Supplementary file 1 — Supporting Information [file VMS3-9-2309-s001.tif]

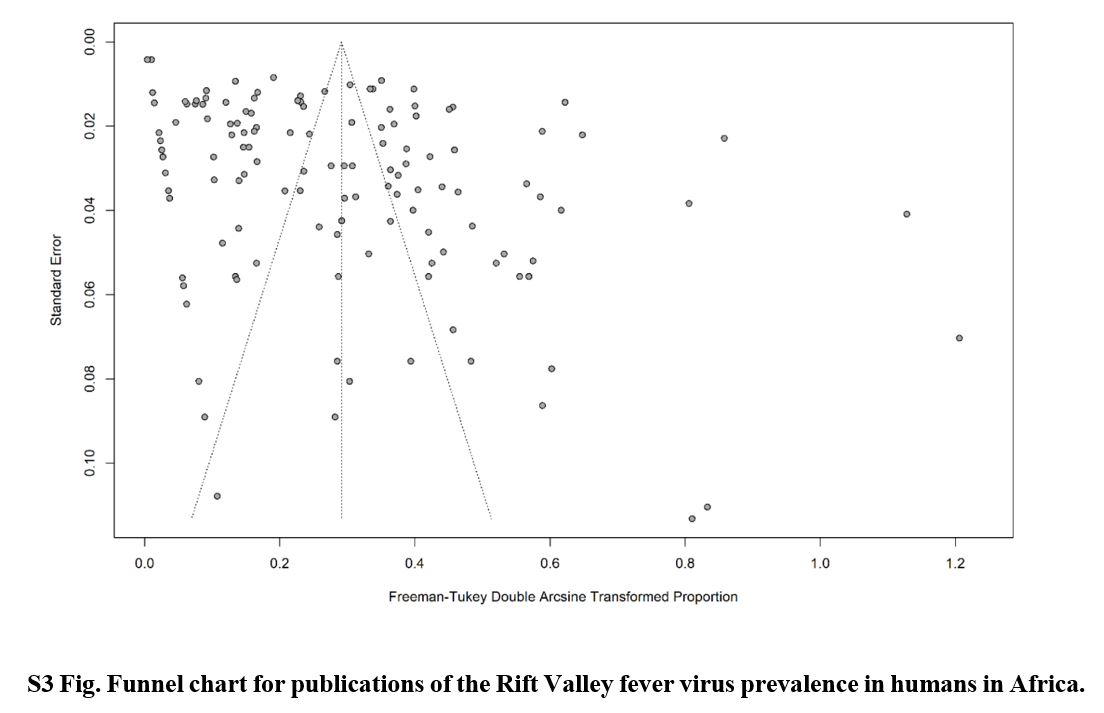

Supplement: Supplementary file 3 — Supporting Information [file VMS3-9-2309-s002.tif]

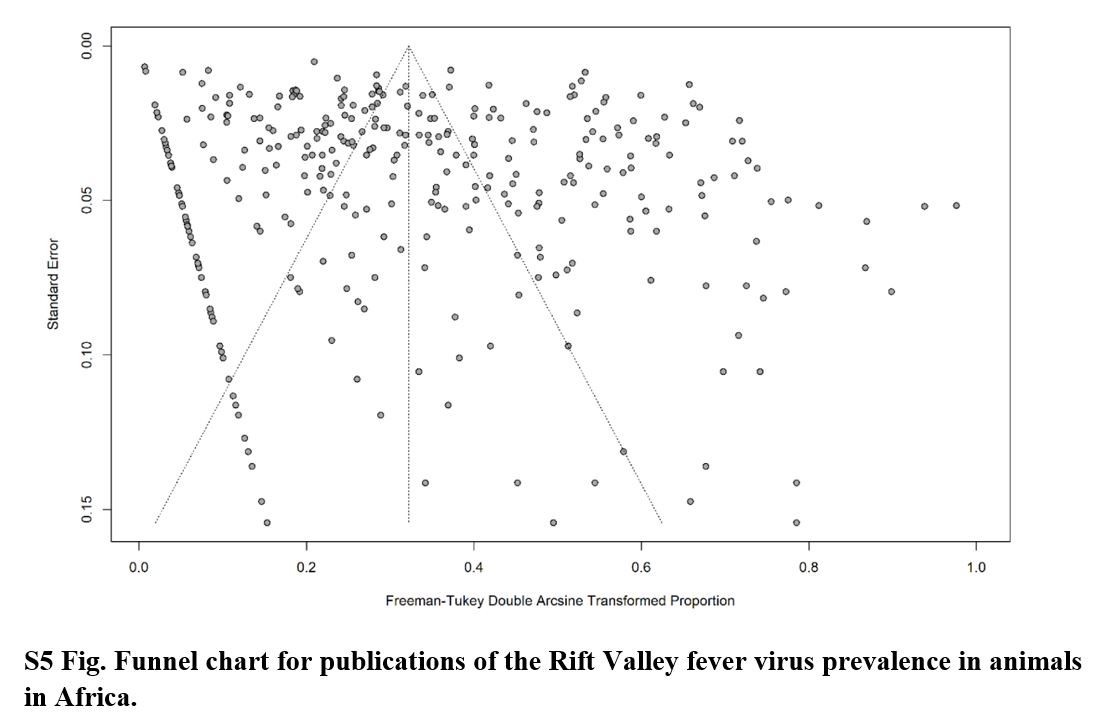

Supplement: Supplementary file 5 — Supporting Information [file VMS3-9-2309-s004.tif]
