# Supplementary material for: Contemporary epidemiological data of Rift Valley fever virus in humans, mosquitoes and other animal species in Africa: A systematic review and meta‐analysis
Source: Vet Med Sci. 2023 Aug 7;9(5):2309–28. doi: 10.1002/vms3.1238 (PMC10508527; doi:10.1002/vms3.1238)
Supplement: Supplementary file 2 — Supporting Information [file VMS3-9-2309-s006.docx]

**Study**

**Positive**

**Total**

**Prevalence (%)**

**95% CI**

**Weight**

**Human_prevalence_Current infection**

Ahmed, 2000_Sudan Anyangu, 2010_Kenya Aradaib, 2013_Kenya Aradaib, 2013_Kenya Archer, 2013_South Africa Bob, 2017_Mauritania Bob, 2022

Bonney, 2013_Ghana Boushab, 2015_Mauritania

Centers for Disease Control and Prevention (CDC), 2007_Kenya Durand, 2003_Chad

Faye, 2007_Mauritania Faye, 2007_Mauritania Grolla, 2012_Kenya Gudo, 2016_Mozambique

Guillebaud, 2018_Madagascar Hassan, 2020_Kenya

Hassan, 2020_Kenya Lagare, 2019_Niger

Makiala−Mandanda, 2018_DemocraticRepublic of the Congo Mohamed, 2010_Tanzania

Mohamed, 2019_Sudan Munyua, 2010_Kenya Nabeth, 2001_Mauritania Nguku, 2010_Kenya

Shieh, 2010_Kenya, Tanzania, Somalia Sow, 2014_Mauritania

Sow, 2014_Mauritania Sow, 2014_Senegal Sow, 2016_Senegal Sow, 2016_Senegal Ushijima, 2021

**Random effect meta−analysis Prediction interval**

Heterogeneity: *I* ^2^ = 99.3% [99.2%; 99.4%], τ^2^ = 0.0632, *p* = 0

44

202

52

18

302

56

4

0

26

10

2

25

22

10

0

1

23

1

9

0

8

9

88

2

272

11

21

13

0

0

1

0

**.**

50

1042

156

100

2009

184

1149

258

212

19

31

98

80

33

200

682

80

80

399

453

511

119

169

90

475

20

288

41

334

535

13845

1189

**24931**

**.**

88.00

19.39

33.33

18.00

15.03

30.43

0.35

0.00

12.26

52.63

6.45

25.51

27.50

30.30

0.00

0.15

28.75

1.25

2.26

0.00

1.57

7.56

52.07

2.22

57.26

55.00

7.29

31.71

0.00

0.00

0.01

0.00

**12.33**

[75.69; 95.47]

[17.03; 21.92]

[26.00; 41.32]

[11.03; 26.95]

[13.50; 16.67]

[23.88; 37.63]

[ 0.09; 0.89]

[ 0.00; 1.42]

[ 8.17; 17.45]

[28.86; 75.55]

[ 0.79; 21.42]

[17.24; 35.31]

[18.10; 38.62]

[15.59; 48.71]

[ 0.00; 1.83]

[ 0.00; 0.81]

[19.18; 39.95]

[ 0.03; 6.77]

[ 1.04; 4.24]

[ 0.00; 0.81]

[ 0.68; 3.06]

[ 3.52; 13.87]

[44.27; 59.80]

[ 0.27; 7.80]

[52.68; 61.76]

[31.53; 76.94]

[ 4.57; 10.93]

[18.08; 48.09]

[ 0.00; 1.10]

[ 0.00; 0.69]

[ 0.00; 0.04]

[ 0.00; 0.31]

**[ 6.98; 18.87]**

**[ 0.00; 60.14]**

0.7%

0.8%

0.8%

0.7%

0.8%

0.8%

0.8%

0.8%

0.8%

0.6%

0.6%

0.7%

0.7%

0.6%

0.8%

0.8%

0.7%

0.7%

0.8%

0.8%

0.8%

0.7%

0.8%

0.7%

0.8%

0.6%

0.8%

0.7%

0.8%

0.8%

0.8%

0.8%

**23.8%**

**−−**

**Human_prevalence_Past infection**

Ahmed, 2018_Tanzania Ahmed, 2018_Tanzania Andayi, 2014_Djibouti

Andriamandimby, 2010_Madagascar Archer, 2011_South Africa

Bett, 2019_Kenya Bosworth, 2016_Tunisia Bosworth, 2016_Tunisia Budodo, 2020_Tanzania Bukbuk, 2014_Nigeria Bukbuk, 2014_Nigeria Clements, 2019_Uganda Cook, 2017_Kenya Cook, 2017_Kenya Durand, 2003_Chad Enem, 2020_Guinea Faye, 2007_Mauritania Fokam, 2010_Cameroon Gray, 2015_Kenya

Gray, 2015_Madagascar Grossi−soyster, 2017_Kenya Gudo, 2016_Mozambique Gudo, 2016_Mozambique Hassanain, 2010_Sudan Heinrich, 2012_Tanzania Ibrahim, 2021_Ethiopia LaBeaud, 2008_Kenya LaBeaud, 2011_Kenya LaBeaud, 2015_Kenya Lagare, 2019_Niger Marrama, 2005_Senegal Mease, 2011_Kenya Msimang, 2019_South Africa Msimang, 2019_South Africa Muiruri, 2015_Kenya Nabeth, 2001_Mauritania

Nakouné, 2016_Central African Republic Nakounne, 2001_Cameroon

Nakounne, 2001_Central African Republic Nguku, 2010_Kenya

O'Hearn, 2016_Sierra Leone Ochieng, 2015_Kenya Opayele, 2018_Nigeria Oragwa, 2022

Paweska, 2005_Kenya

Paweska, 2005_Kenya; South Africa;Tanzania; Uganda Paweska, 2005_South Africa

Paweska, 2005_Tanzania Paweska, 2005_Uganda

Paweska, 2007_Kenya, South Africa, Tanzania, Uganda, Zimbabwe Paweska, 2021

Pourrut, 2010_Gabon Pourrut, 2010_Gabon

Sadeuh−Mba, 2018_Cameroon Sanderson, 2020_Botswana Schwarz, 2012_Madagascar Sow, 2014_Mauritania

Sow, 2014_Senegal Sow, 2016_Senegal

Spiropoulou, 2018_Uganda Sumaye, 2015_Tanzania Swai, 2009_Tanzania Tigoi, 2015_Kenya

Tigoi, 2020_Kenya Ushijima, 2021

Wolff, 2018_South Africa Wolff, 2018_South Africa Woods, 2002_Kenya Youssef, 2009_Egypt

**Random effect meta−analysis Prediction interval**

Heterogeneity: *I* ^2^ = 98.1% [97.9%; 98.3%], τ^2^ = 0.0195, *p* = 0

30

4

20

214

302

170

3

0

20

42

34

48

15

14

0

8

10

0

51

2

51

1

10

122

64

25

33

27

164

8

79

8

62

11

38

22

56

0

124

184

12

57

14

39

123

215

2

63

1

350

36

21

124

17

64

4

24

3

24

85

71

8

74

411

55

0

0

10

6

**.**

521

230

914

1995

2009

552

38

181

122

297

271

1744

1861

553

31

129

98

79

430

127

2871

78

200

149

1228

190

248

92

1082

399

1520

1141

685

138

211

90

335

21

1791

970

667

1057

265

196

806

2400

232

220

109

2967

1395

867

3456

137

1276

1244

288

334

535

655

606

199

379

1210

387

74

64

53

43

**47742**

**.**

5.76

1.74

2.19

10.73

15.03

30.80

7.89

0.00

16.39

14.14

12.55

2.75

0.81

2.53

0.00

6.20

10.20

0.00

11.86

1.57

1.78

1.28

5.00

81.88

5.21

13.16

13.31

29.35

15.16

2.01

5.20

0.70

9.05

7.97

18.01

24.44

16.72

0.00

6.92

18.97

1.80

5.39

5.28

19.90

15.26

8.96

0.86

28.64

0.92

11.80

2.58

2.42

3.59

12.41

5.02

0.32

8.33

0.90

4.49

12.98

11.72

4.02

19.53

33.97

14.21

0.00

0.00

18.87

13.95

**7.93**

[ 3.92; 8.12]

[ 0.48; 4.39]

[ 1.34; 3.36]

[ 9.40; 12.17]

[13.50; 16.67]

[26.97; 34.83]

[ 1.66; 21.38]

[ 0.00; 2.02]

[10.31; 24.18]

[10.39; 18.63]

[ 8.85; 17.09]

[ 2.04; 3.63]

[ 0.45; 1.33]

[ 1.39; 4.21]

[ 0.00; 11.22]

[ 2.72; 11.85]

[ 5.00; 17.97]

[ 0.00; 4.56]

[ 8.96; 15.30]

[ 0.19; 5.57]

[ 1.33; 2.33]

[ 0.03; 6.94]

[ 2.42; 9.00]

[74.74; 87.71]

[ 4.04; 6.61]

[ 8.70; 18.81]

[ 9.34; 18.18]

[20.31; 39.76]

[13.07; 17.43]

[ 0.87; 3.91]

[ 4.14; 6.44]

[ 0.30; 1.38]

[ 7.01; 11.45]

[ 4.05; 13.81]

[13.07; 23.87]

[16.00; 34.64]

[12.88; 21.15]

[ 0.00; 16.11]

[ 5.79; 8.20]

[16.55; 21.58]

[ 0.93; 3.12]

[ 4.11; 6.93]

[ 2.92; 8.71]

[14.55; 26.18]

[12.85; 17.93]

[ 7.85; 10.17]

[ 0.10; 3.08]

[22.76; 35.10]

[ 0.02; 5.01]

[10.66; 13.01]

[ 1.81; 3.55]

[ 1.51; 3.68]

[ 2.99; 4.26]

[ 7.40; 19.12]

[ 3.88; 6.36]

[ 0.09; 0.82]

[ 5.41; 12.15]

[ 0.19; 2.60]

[ 2.90; 6.60]

[10.50; 15.79]

[ 9.26; 14.55]

[ 1.75; 7.77]

[15.65; 23.88]

[31.30; 36.71]

[10.89; 18.09]

[ 0.00; 4.86]

[ 0.00; 5.60]

[ 9.44; 31.97]

[ 5.30; 27.93]

**[ 6.15; 9.90]**

**[ 0.00; 29.07]**

0.8%

0.8%

0.8%

0.8%

0.8%

0.8%

0.7%

0.8%

0.7%

0.8%

0.8%

0.8%

0.8%

0.8%

0.6%

0.8%

0.7%

0.7%

0.8%

0.8%

0.8%

0.7%

0.8%

0.8%

0.8%

0.8%

0.8%

0.7%

0.8%

0.8%

0.8%

0.8%

0.8%

0.8%

0.8%

0.7%

0.8%

0.6%

0.8%

0.8%

0.8%

0.8%

0.8%

0.8%

0.8%

0.8%

0.8%

0.8%

0.7%

0.8%

0.8%

0.8%

0.8%

0.8%

0.8%

0.8%

0.8%

0.8%

0.8%

0.8%

0.8%

0.8%

0.8%

0.8%

0.8%

0.7%

0.7%

0.7%

0.7%

**52.8%**

**−−**

**Human_prevalence_Recent infection** Ahmed, 2018_Tanzania Andriamandimby, 2010_Madagascar Aradaib, 2013_Kenya

Baudin, 2016_Sudan Bob, 2017_Mauritania Bob, 2022

Bosworth, 2016_Tunisia Bosworth, 2016_Tunisia Durand, 2003_Chad Faye, 2007_Mauritania Hassan, 2020_Kenya

Mohamed, 2010_Tanzania Nabeth, 2001_Mauritania

Nakouné, 2016_Central African Republic Nakounne, 2001_Cameroon

Nakounne, 2001_Central African Republic Nguku, 2010_Kenya

Paweska, 2021

Rugarabamu, 2022 Sanderson, 2020_Botswana

Schoepp, 2014_Sierra Leone; Liberia; Guinea Sow, 2014_Mauritania

Sow, 2014_Senegal Sow, 2016_Senegal Sow, 2016_Senegal

Spiropoulou, 2018_Uganda Sumaye, 2015_Tanzania Tigoi, 2015_Kenya

Tigoi, 2020_Kenya Woods, 2002_Kenya Woods, 2002_Kenya

**Random effect meta−analysis Prediction interval**

Heterogeneity: *I* ^2^ = 98.7% [98.5%; 98.9%], τ^2^ = 0.0258, *p* = 0

6

219

23

28

17

6

0

15

0

13

6

186

15

0

0

0

122

11

8

7

5

26

0

11

0

10

16

0

17

9

31

**.**

751

1995

156

130

184

1149

38

181

31

80

80

511

90

335

21

1723

970

1395

308

1276

253

288

334

535

13845

655

606

379

1210

43

202

**29754**

**.**

0.80

10.98

14.74

21.54

9.24

0.52

0.00

8.29

0.00

16.25

7.50

36.40

16.67

0.00

0.00

0.00

12.58

0.79

2.60

0.55

1.98

9.03

0.00

2.06

0.00

1.53

2.64

0.00

1.40

20.93

15.35

**4.29**

[ 0.29; 1.73]

[ 9.64; 12.43]

[ 9.58; 21.30]

[14.81; 29.60]

[ 5.47; 14.38]

[ 0.19; 1.13]

[ 0.00; 9.25]

[ 4.71; 13.30]

[ 0.00; 11.22]

[ 8.95; 26.18]

[ 2.80; 15.61]

[32.22; 40.74]

[ 9.64; 26.00]

[ 0.00; 1.10]

[ 0.00; 16.11]

[ 0.00; 0.21]

[10.56; 14.83]

[ 0.39; 1.41]

[ 1.13; 5.05]

[ 0.22; 1.13]

[ 0.64; 4.55]

[ 5.98; 12.95]

[ 0.00; 1.10]

[ 1.03; 3.65]

[ 0.00; 0.03]

[ 0.73; 2.79]

[ 1.52; 4.25]

[ 0.00; 0.97]

[ 0.82; 2.24]

[10.04; 36.04]

[10.67; 21.07]

**[ 2.15; 7.06]**

**[ 0.00; 27.21]**

0.8%

0.8%

0.8%

0.8%

0.8%

0.8%

0.7%

0.8%

0.6%

0.7%

0.7%

0.8%

0.7%

0.8%

0.6%

0.8%

0.8%

0.8%

0.8%

0.8%

0.8%

0.8%

0.8%

0.8%

0.8%

0.8%

0.8%

0.8%

0.8%

0.7%

0.8%

**23.5%**

**−−**

**Overall random effect meta−analysis Prediction interval**

Heterogeneity: *I* ^2^ = 99.0% [98.9%; 99.0%], τ^2^ = 0.0328, *p* = 0

Test for subgroup differences: χ^2^ = 8.36, df = 2 (*p* = 0.0153)

2

**102427**

# S2 Fig. Pooled prevalence estimates of Rift Valley fever virus infections in humans in Africa.

0 20 40 60 80

**7.82**

**[ 6.16; 9.64]**

**[ 0.00; 36.38]**

**100.0%**
