# Supplementary material for: Contemporary epidemiological data of Rift Valley fever virus in humans, mosquitoes and other animal species in Africa: A systematic review and meta‐analysis
Source: Vet Med Sci. 2023 Aug 7;9(5):2309–28. doi: 10.1002/vms3.1238 (PMC10508527; doi:10.1002/vms3.1238)
Supplement: Supplementary file 4 — Supporting Information [file VMS3-9-2309-s005.docx]

| **Study**  **Animal_prevalence_Current infection** | **Positive** | **Total** |  |  |  |  | **Prevalence (%) 95% CI Weight** |
| --- | --- | --- | --- | --- | --- | --- | --- |
| Bird, 2008_Kenya_Buffalo | 4 | 26 |  |  |  |  | 15.38 [ 4.36; 34.87] 0.2% |
| Bird, 2008_Kenya_Camel | 0 | 110 |  |  |  |  | 0.00 [ 0.00; 3.30] 0.3% |
| Bird, 2008_Kenya_Cattle | 72 | 1226 |  |  |  |  | 5.87 [ 4.62; 7.34] 0.3% |
| Bird, 2008_Kenya_Goat | 39 | 1181 |  |  |  |  | 3.30 [ 2.36; 4.49] 0.3% |
| Bird, 2008_Kenya_Sheep | 48 | 641 |  |  |  |  | 7.49 [ 5.57; 9.81] 0.3% |
| Bird, 2008_Kenya_Warthog (Phaecochoerus africanus) | 0 | 44 |  |  |  |  | 0.00 [ 0.00; 8.04] 0.3% |
| Capobianco Dondona, 2016_Namibia_Springbok | 18 | 200 |  |  |  |  | 9.00 [ 5.42; 13.85] 0.3% |
| Capobianco Dondona, 2016_Namibia_Wildebeest | 0 | 50 |  |  |  |  | 0.00 [ 0.00; 7.11] 0.3% |
| Chengula, 2014_Tanzania_Cattle | 11 | 53 |  |  |  |  | 20.75 [10.84; 34.11] 0.3% |
| Chengula, 2014_Tanzania_Goat | 7 | 38 |  |  |  |  | 18.42 [ 7.74; 34.33] 0.3% |
| Chengula, 2014_Tanzania_Sheep | 1 | 17 |  |  |  |  | 5.88 [ 0.15; 28.69] 0.2% |
| Cichon, 2021_Mauritania_Goat | 0 | 234 |  |  |  |  | 0.00 [ 0.00; 1.56] 0.3% |
| Cichon, 2021_Mauritania_Sheep | 0 | 253 |  |  |  |  | 0.00 [ 0.00; 1.45] 0.3% |
| Dutuze, 2020_Rwanda_Cattle | 44 | 157 |  |  |  |  | 28.03 [21.16; 35.74] 0.3% |
| Dutuze, 2020_Rwanda_Goat | 12 | 28 |  |  |  |  | 42.86 [24.46; 62.82] 0.3% |
| El Mamy, 2010_Mauritania_Camel | 4 | 14 |  |  |  |  | 28.57 [ 8.39; 58.10] 0.2% |
| Faye, 2007_Mauritania_Goat | 0 | 78 |  |  |  |  | 0.00 [ 0.00; 4.62] 0.3% |
| Faye, 2007_Mauritania_Sheep | 0 | 13 |  |  |  |  | 0.00 [ 0.00; 24.71] 0.2% |
| Lagare, 2019_Niger_Animal unspecified | 0 | 39 |  |  |  |  | 0.00 [ 0.00; 9.03] 0.3% |
| Maganga, 2017_Gabon_Goat | 0 | 106 |  |  |  |  | 0.00 [ 0.00; 3.42] 0.3% |
| Maganga, 2017_Gabon_Sheep | 0 | 95 |  |  |  |  | 0.00 [ 0.00; 3.81] 0.3% |
| Nabeth, 2001_Mauritania_Camel | 0 | 39 |  |  |  |  | 0.00 [ 0.00; 9.03] 0.3% |
| Nabeth, 2001_Mauritania_Cattle | 0 | 69 |  |  |  |  | 0.00 [ 0.00; 5.21] 0.3% |
| Nabeth, 2001_Mauritania_Goat | 5 | 141 |  |  |  |  | 3.55 [ 1.16; 8.08] 0.3% |
| Nabeth, 2001_Mauritania_Rodents | 0 | 73 |  |  |  |  | 0.00 [ 0.00; 4.93] 0.3% |
| Nabeth, 2001_Mauritania_Sheep | 6 | 89 |  |  |  |  | 6.74 [ 2.51; 14.10] 0.3% |
| Roug, 2020_Tanzania_Buffalo | 0 | 33 |  |  |  |  | 0.00 [ 0.00; 10.58] 0.3% |
| Youssef, 2002_Egypt_Rattus rattus | 29 | 300 |  |  |  |  | 9.67 [ 6.57; 13.59] 0.3% |
| **Random effect meta−analysis** |  | **5347** |  |  |  |  | **3.56 [ 1.68; 5.96] 7.8%** |

**Prediction interval**

Heterogeneity: *I* 2 = 91.5% [88.9%; 93.5%], τ2 = 0.0156, *p* < 0.0001

| **Animal_prevalence_Past infection** |  | | | | | | | | | | | | | | | | |
| --- | --- | --- | --- | --- | --- | --- | --- | --- | --- | --- | --- | --- | --- | --- | --- | --- | --- |
| Abakar, 2014_Chad_Cattle | 270 | 715 |  |  |  |  |  |  |  |  |  |  |  |  |  | 37.76 | [34.20; 41.43] 0.3% |
| Abakar, 2014_Chad_Goat | 27 | 144 |  |  |  |  |  |  |  |  |  |  |  |  |  | 18.75 | [12.73; 26.10] 0.3% |
| Abakar, 2014_Chad_Sheep | 7 | 65 |  |  |  |  |  |  |  |  |  |  |  |  |  | 10.77 | [ 4.44; 20.94] 0.3% |
| Abdallah, 2016_Sudan_Camel | 23 | 240 |  |  |  |  |  |  |  |  |  |  |  |  |  | 9.58 | [ 6.17; 14.03] 0.3% |
| Adamu, 2020_Nigeria_Sheep | 9 | 200 |  |  |  |  |  |  |  |  |  |  |  |  |  | 4.50 | [ 2.08; 8.37] 0.3% |
| Adamu, 2021_Nigeria_Camel | 143 | 720 |  |  |  |  |  |  |  |  |  |  |  |  |  | 19.86 | [17.01; 22.97] 0.3% |
| Adesiyun, 2020_South Africa_Cattle | 1 | 184 |  |  |  |  |  |  |  |  |  |  |  |  |  | 0.54 | [ 0.01; 2.99] 0.3% |
| Andriamandimby, 2018_Mali_Cattle | 34 | 941 |  |  |  |  |  |  |  |  |  |  |  |  |  | 3.61 | [ 2.51; 5.01] 0.3% |
| Atuman, 2022_Nigeria_Cattle | 34 | 300 |  |  |  |  |  |  |  |  |  |  |  |  |  | 11.33 | [ 7.98; 15.48] 0.3% |
| Atuman, 2022_Nigeria_Eland (Taurotragus otyX) | 3 | 24 |  |  |  |  |  |  |  |  |  |  |  |  |  | 12.50 | [ 2.66; 32.36] 0.2% |
| Atuman, 2022_Nigeria_Waterbuck | 4 | 111 |  |  |  |  |  |  |  |  |  |  |  |  |  | 3.60 | [ 0.99; 8.97] 0.3% |
| Atuman, 2022_Nigeria_Wildebeest | 1 | 12 |  |  |  |  |  |  |  |  |  |  |  |  |  | 8.33 | [ 0.21; 38.48] 0.2% |
| Atuman, 2022_Nigeria_Zebra | 0 | 53 |  |  |  |  |  |  |  |  |  |  |  |  |  | 0.00 | [ 0.00; 6.72] 0.3% |
| Ayari−Fakhfakh, 2011_Tunisia_Goat | 14 | 119 |  |  |  |  |  |  |  |  |  |  |  |  |  | 11.76 | [ 6.58; 18.95] 0.3% |
| Ayari−Fakhfakh, 2011_Tunisia_Sheep | 15 | 263 |  |  |  |  |  |  |  |  |  |  |  |  |  | 5.70 | [ 3.23; 9.23] 0.3% |
| Beechler, 2015_South Africa_Buffalo | 5 | 227 |  |  |  |  |  |  |  |  |  |  |  |  |  | 2.20 | [ 0.72; 5.07] 0.3% |
| Bett, 2019_Kenya_Livestock (goat, sheep, cattle) | 491 | 1932 |  |  |  |  |  |  |  |  |  |  |  |  |  | 25.41 | [23.49; 27.42] 0.3% |
| Bird, 2008_Kenya_Buffalo | 0 | 26 |  |  |  |  |  |  |  |  |  |  |  |  |  | 0.00 | [ 0.00; 13.23] 0.2% |
| Bird, 2008_Kenya_Camel | 23 | 110 |  |  |  |  |  |  |  |  |  |  |  |  |  | 20.91 | [13.74; 29.70] 0.3% |
| Bird, 2008_Kenya_Cattle | 42 | 1226 |  |  |  |  |  |  |  |  |  |  |  |  |  | 3.43 | [ 2.48; 4.60] 0.3% |
| Bird, 2008_Kenya_Goat | 41 | 1181 |  |  |  |  |  |  |  |  |  |  |  |  |  | 3.47 | [ 2.50; 4.68] 0.3% |
| Bird, 2008_Kenya_Sheep | 17 | 641 |  |  |  |  |  |  |  |  |  |  |  |  |  | 2.65 | [ 1.55; 4.21] 0.3% |
| Bird, 2008_Kenya_Warthog (Phaecochoerus africanus) | 0 | 44 |  |  |  |  |  |  |  |  |  |  |  |  |  | 0.00 | [ 0.00; 8.04] 0.3% |
| Blomstrom, 2016_Mozambique_Goat | 47 | 187 |  |  |  |  |  |  |  |  |  |  |  |  |  | 25.13 | [19.09; 31.98] 0.3% |
| Blomstrom, 2016_Mozambique_Sheep | 80 | 181 |  |  |  |  |  |  |  |  |  |  |  |  |  | 44.20 | [36.84; 51.75] 0.3% |
| Boussini, 2014_Burkina Faso_Camel | 26 | 212 |  |  |  |  |  |  |  |  |  |  |  |  |  | 12.26 | [ 8.17; 17.45] 0.3% |
| Boussini, 2014_Burkina Faso_Cattle | 18 | 120 |  |  |  |  |  |  |  |  |  |  |  |  |  | 15.00 | [ 9.14; 22.67] 0.3% |
| Boussini, 2014_Burkina Faso_Goat | 8 | 200 |  |  |  |  |  |  |  |  |  |  |  |  |  | 4.00 | [ 1.74; 7.73] 0.3% |
| Boussini, 2014_Burkina Faso_Sheep | 14 | 200 |  |  |  |  |  |  |  |  |  |  |  |  |  | 7.00 | [ 3.88; 11.47] 0.3% |
| Bronsvoort, 2022_Cameroon_Cattle | 117 | 1498 |  |  |  |  |  |  |  |  |  |  |  |  |  | 7.81 | [ 6.50; 9.29] 0.3% |
| Budasha, 2018_Uganda_Cattle | 70 | 341 |  |  |  |  |  |  |  |  |  |  |  |  |  | 20.53 | [16.37; 25.21] 0.3% |
| Budasha, 2018_Uganda_Goat | 12 | 336 |  |  |  |  |  |  |  |  |  |  |  |  |  | 3.57 | [ 1.86; 6.16] 0.3% |
| Budasha, 2018_Uganda_Sheep | 22 | 323 |  |  |  |  |  |  |  |  |  |  |  |  |  | 6.81 | [ 4.32; 10.13] 0.3% |
| Capobianco Dondona, 2016_Namibia_Springbok | 70 | 200 |  |  |  |  |  |  |  |  |  |  |  |  |  | 35.00 | [28.41; 42.05] 0.3% |
| Capobianco Dondona, 2016_Namibia_Wildebeest | 12 | 50 |  |  |  |  |  |  |  |  |  |  |  |  |  | 24.00 | [13.06; 38.17] 0.3% |
| Chambaro, 2022_Zambia_Buffalo | 30 | 128 |  |  |  |  |  |  |  |  |  |  |  |  |  | 23.44 | [16.41; 31.74] 0.3% |
| Chambaro, 2022_Zambia_Goat | 16 | 259 |  |  |  |  |  |  |  |  |  |  |  |  |  | 6.18 | [ 3.57; 9.84] 0.3% |
| Chambaro, 2022_Zambia_Hartebeest | 18 | 41 |  |  |  |  |  |  |  |  |  |  |  |  |  | 43.90 | [28.47; 60.25] 0.3% |
| Chambaro, 2022_Zambia_Impala | 46 | 98 |  |  |  |  |  |  |  |  |  |  |  |  |  | 46.94 | [36.78; 57.29] 0.3% |
| Chambaro, 2022_Zambia_Sheep | 0 | 13 |  |  |  |  |  |  |  |  |  |  |  |  |  | 0.00 | [ 0.00; 24.71] 0.2% |
| Chambaro, 2022_Zambia_Warthog (Phaecochoerus africanus) | 2 | 18 |  |  |  |  |  |  |  |  |  |  |  |  |  | 11.11 | [ 1.38; 34.71] 0.2% |
| Chengula, 2014_Tanzania_Cattle | 41 | 106 |  |  |  |  |  |  |  |  |  |  |  |  |  | 38.68 | [29.38; 48.63] 0.3% |
| Chengula, 2014_Tanzania_Goat | 32 | 82 |  |  |  |  |  |  |  |  |  |  |  |  |  | 39.02 | [28.44; 50.43] 0.3% |
| Chengula, 2014_Tanzania_Sheep | 6 | 12 |  |  |  |  |  |  |  |  |  |  |  |  |  | 50.00 | [21.09; 78.91] 0.2% |
| Chevalier, 2005_Senegal_Small ruminants (Goat and Sheep) | 3 | 610 |  |  |  |  |  |  |  |  |  |  |  |  |  | 0.49 | [ 0.10; 1.43] 0.3% |
| Chevalier, 2011_Madagascar_Cattle | 250 | 894 |  |  |  |  |  |  |  |  |  |  |  |  |  | 27.96 | [25.04; 31.03] 0.3% |
| Cichon, 2021_Mauritania_Small ruminants (Goat and Sheep) | 80 | 458 |  |  |  |  |  |  |  |  |  |  |  |  |  | 17.47 | [14.10; 21.26] 0.3% |
| Cosseddu, 2021_Mauritania_Camel | 72 | 159 |  |  |  |  |  |  |  |  |  |  |  |  |  | 45.28 | [37.39; 53.36] 0.3% |
| Cosseddu, 2021_Mauritania_Cattle | 19 | 118 |  |  |  |  |  |  |  |  |  |  |  |  |  | 16.10 | [ 9.98; 24.00] 0.3% |
| Di Nardo, 2014_Algeria_Camel, Goat, Sheep | 11 | 982 |  |  |  |  |  |  |  |  |  |  |  |  |  | 1.12 | [ 0.56; 2.00] 0.3% |
| Dione, 2022_Mali_Cattle | 39 | 304 |  |  |  |  |  |  |  |  |  |  |  |  |  | 12.83 | [ 9.28; 17.12] 0.3% |
| Dione, 2022_Mali_Goat | 9 | 290 |  |  |  |  |  |  |  |  |  |  |  |  |  | 3.10 | [ 1.43; 5.81] 0.3% |
| Dione, 2022_Mali_Sheep | 15 | 318 |  |  |  |  |  |  |  |  |  |  |  |  |  | 4.72 | [ 2.66; 7.66] 0.3% |
| Durand, 2020_Senegal_Cattle | 10 | 70 |  |  |  |  |  |  |  |  |  |  |  |  |  | 14.29 | [ 7.07; 24.71] 0.3% |
| Durand, 2020_Senegal_Goat | 10 | 54 |  |  |  |  |  |  |  |  |  |  |  |  |  | 18.52 | [ 9.25; 31.43] 0.3% |
| Durand, 2020_Senegal_Sheep | 24 | 168 |  |  |  |  |  |  |  |  |  |  |  |  |  | 14.29 | [ 9.37; 20.51] 0.3% |
| Ebogo−Belobo, 2022_Cameroon_Goat | 7 | 144 |  |  |  |  |  |  |  |  |  |  |  |  |  | 4.86 | [ 1.98; 9.76] 0.3% |
| Ebogo−Belobo, 2022_Cameroon_Sheep | 3 | 44 |  |  |  |  |  |  |  |  |  |  |  |  |  | 6.82 | [ 1.43; 18.66] 0.3% |
| Eckstein, 2022_Tunisia_Camel | 5 | 500 |  |  |  |  |  |  |  |  |  |  |  |  |  | 1.00 | [ 0.33; 2.32] 0.3% |
| El−Harrak, 2011_Morocco_Camel | 15 | 100 |  |  |  |  |  |  |  |  |  |  |  |  |  | 15.00 | [ 8.65; 23.53] 0.3% |
| El Bahgy , 2018_Egypt_Camel | 27 | 200 |  |  |  |  |  |  |  |  |  |  |  |  |  | 13.50 | [ 9.09; 19.03] 0.3% |
| El Mamy, 2011_Mauritania_Camel | 91 | 279 |  |  |  |  |  |  |  |  |  |  |  |  |  | 32.62 | [27.15; 38.46] 0.3% |
| El Mamy, 2011_Mauritania_Small ruminants (Goat and Sheep) | 114 | 262 |  |  |  |  |  |  |  |  |  |  |  |  |  | 43.51 | [37.42; 49.75] 0.3% |
| Endale, 2021_Ethiopia_Cattle | 20 | 397 |  |  |  |  |  |  |  |  |  |  |  |  |  | 5.04 | [ 3.10; 7.67] 0.3% |
| EVANS, 2008_Kenya_Black rhino | 14 | 43 |  |  |  |  |  |  |  |  |  |  |  |  |  | 32.56 | [19.08; 48.54] 0.3% |
| EVANS, 2008_Kenya_Buffalo | 49 | 265 |  |  |  |  |  |  |  |  |  |  |  |  |  | 18.49 | [14.00; 23.70] 0.3% |
| EVANS, 2008_Kenya_Elephant | 5 | 83 |  |  |  |  |  |  |  |  |  |  |  |  |  | 6.02 | [ 1.98; 13.50] 0.3% |
| EVANS, 2008_Kenya_Giraffe | 0 | 81 |  |  |  |  |  |  |  |  |  |  |  |  |  | 0.00 | [ 0.00; 4.45] 0.3% |
| EVANS, 2008_Kenya_Kongoni | 0 | 10 |  |  |  |  |  |  |  |  |  |  |  |  |  | 0.00 | [ 0.00; 30.85] 0.2% |
| EVANS, 2008_Kenya_Kudu | 5 | 10 |  |  |  |  |  |  |  |  |  |  |  |  |  | 50.00 | [18.71; 81.29] 0.2% |
| EVANS, 2008_Kenya_Lion | 0 | 34 |  |  |  |  |  |  |  |  |  |  |  |  |  | 0.00 | [ 0.00; 10.28] 0.3% |
| EVANS, 2008_Kenya_Warthog (Phaecochoerus africanus) | 2 | 81 |  |  |  |  |  |  |  |  |  |  |  |  |  | 2.47 | [ 0.30; 8.64] 0.3% |
| EVANS, 2008_Kenya_Waterbuck | 2 | 10 |  |  |  |  |  |  |  |  |  |  |  |  |  | 20.00 | [ 2.52; 55.61] 0.2% |
| EVANS, 2008_Kenya_Zebra | 1 | 102 |  |  |  |  |  |  |  |  |  |  |  |  |  | 0.98 | [ 0.02; 5.34] 0.3% |
| Fafetine, 2012_Mozambique_Goat | 65 | 657 |  |  |  |  |  |  |  |  |  |  |  |  |  | 9.89 | [ 7.72; 12.44] 0.3% |
| Fafetine, 2012_Mozambique_Sheep | 92 | 605 |  |  |  |  |  |  |  |  |  |  |  |  |  | 15.21 | [12.44; 18.32] 0.3% |
| Fafetine, 2013_Mozambique_Goat | 52 | 449 |  |  |  |  |  |  |  |  |  |  |  |  |  | 11.58 | [ 8.77; 14.91] 0.3% |
| Fafetine, 2013_Mozambique_Sheep | 29 | 313 |  |  |  |  |  |  |  |  |  |  |  |  |  | 9.27 | [ 6.29; 13.04] 0.3% |
| Fafetine, 2014_Mozambique_Small ruminants (Goat and Sheep) | 49 | 127 |  |  |  |  |  |  |  |  |  |  |  |  |  | 38.58 | [30.08; 47.63] 0.3% |
| Fagbo, 2014_South Africa_Buffalo | 15 | 240 |  |  |  |  |  |  |  |  |  |  |  |  |  | 6.25 | [ 3.54; 10.10] 0.3% |
| Faye, 2007_Mauritania_Goat | 0 | 48 |  |  |  |  |  |  |  |  |  |  |  |  |  | 0.00 | [ 0.00; 7.40] 0.3% |
| Fischer−Tenhagen, 2000_Kenya, Namibia, South Africa_Rhinoceros | 0 | 272 |  |  |  |  |  |  |  |  |  |  |  |  |  | 0.00 | [ 0.00; 1.35] 0.3% |
| Gora, 2000_Senegal_Rodents | 6 | 140 |  |  |  |  |  |  |  |  |  |  |  |  |  | 4.29 | [ 1.59; 9.09] 0.3% |
| Halawi, 2019_Democratic Republic of the Congo_Cattle | 43 | 677 |  |  |  |  |  |  |  |  |  |  |  |  |  | 6.35 | [ 4.63; 8.46] 0.3% |
| Hassine, 2017_Tunisia_Dromedaries | 0 | 118 |  |  |  |  |  |  |  |  |  |  |  |  |  | 0.00 | [ 0.00; 3.08] 0.3% |
| Horton, 2014_Egypt_Buffalo | 3 | 153 |  |  |  |  |  |  |  |  |  |  |  |  |  | 1.96 | [ 0.41; 5.62] 0.3% |
| Horton, 2014_Egypt_Camel | 0 | 10 |  |  |  |  |  |  |  |  |  |  |  |  |  | 0.00 | [ 0.00; 30.85] 0.2% |
| Horton, 2014_Egypt_Cattle | 2 | 161 |  |  |  |  |  |  |  |  |  |  |  |  |  | 1.24 | [ 0.15; 4.42] 0.3% |
| Horton, 2014_Egypt_Sheep | 0 | 174 |  |  |  |  |  |  |  |  |  |  |  |  |  | 0.00 | [ 0.00; 2.10] 0.3% |
| Ibrahim, 2021_Ethiopia_Camel | 60 | 141 |  |  |  |  |  |  |  |  |  |  |  |  |  | 42.55 | [34.27; 51.15] 0.3% |
| Ibrahim, 2021_Ethiopia_Cattle | 19 | 108 |  |  |  |  |  |  |  |  |  |  |  |  |  | 17.59 | [10.94; 26.10] 0.3% |
| Ibrahim, 2021_Ethiopia_Goat | 15 | 252 |  |  |  |  |  |  |  |  |  |  |  |  |  | 5.95 | [ 3.37; 9.63] 0.3% |
| Ibrahim, 2021_Ethiopia_Sheep | 17 | 229 |  |  |  |  |  |  |  |  |  |  |  |  |  | 7.42 | [ 4.38; 11.62] 0.3% |
| Jäckel, 2013_Mauritania_Camel | 28 | 62 |  |  |  |  |  |  |  |  |  |  |  |  |  | 45.16 | [32.48; 58.32] 0.3% |
| Jäckel, 2013_Mauritania_Small ruminants (Goat and Sheep) | 64 | 93 |  |  |  |  |  |  |  |  |  |  |  |  |  | 68.82 | [58.37; 78.02] 0.3% |
| Jeanmaire, 2011_Madagascar_Cattle | 887 | 3437 |  |  |  |  |  |  |  |  |  |  |  |  |  | 25.81 | [24.35; 27.31] 0.3% |
| Jeanmaire, 2011_Madagascar_Small ruminants (Goat and Sheep) | 244 | 989 |  |  |  |  |  |  |  |  |  |  |  |  |  | 24.67 | [22.01; 27.48] 0.3% |
| Jori, 2015_Botswana_Buffalo | 19 | 150 |  |  |  |  |  |  |  |  |  |  |  |  |  | 12.67 | [ 7.80; 19.07] 0.3% |
| Jori, 2015_Botswana_Cattle | 49 | 863 |  |  |  |  |  |  |  |  |  |  |  |  |  | 5.68 | [ 4.23; 7.44] 0.3% |
| Kading, 2018_Egypt_Bat | 3 | 54 |  |  |  |  |  |  |  |  |  |  |  |  |  | 5.56 | [ 1.16; 15.39] 0.3% |
| Kading, 2018_Uganda_Bat | 5 | 264 |  |  |  |  |  |  |  |  |  |  |  |  |  | 1.89 | [ 0.62; 4.36] 0.3% |
| Kanoute, 2017_Ivory Coast_Cattle | 7 | 192 |  |  |  |  |  |  |  |  |  |  |  |  |  | 3.65 | [ 1.48; 7.37] 0.3% |
| Kanoute, 2017_Ivory Coast_Goat | 0 | 161 |  |  |  |  |  |  |  |  |  |  |  |  |  | 0.00 | [ 0.00; 2.27] 0.3% |
| Kanoute, 2017_Ivory Coast_Sheep | 8 | 333 |  |  |  |  |  |  |  |  |  |  |  |  |  | 2.40 | [ 1.04; 4.68] 0.3% |
| Kifaro, 2014_Tanzania_Goat | 18 | 379 |  |  |  |  |  |  |  |  |  |  |  |  |  | 4.75 | [ 2.84; 7.40] 0.3% |
| Kifaro, 2014_Tanzania_Sheep | 4 | 32 |  |  |  |  |  |  |  |  |  |  |  |  |  | 12.50 | [ 3.51; 28.99] 0.3% |
| LaBeaud, 2011_South Africa_Buffalo | 115 | 550 |  |  |  |  |  |  |  |  |  |  |  |  |  | 20.91 | [17.58; 24.55] 0.3% |
| Lagare, 2019_Niger_Animal unspecified | 24 | 39 |  |  |  |  |  |  |  |  |  |  |  |  |  | 61.54 | [44.62; 76.64] 0.3% |
| Lagerqvist, 2013_Mozambique_Animal unspecified | 149 | 404 |  |  |  |  |  |  |  |  |  |  |  |  |  | 36.88 | [32.16; 41.79] 0.3% |
| LeBreton, 2006_Cameroon_Goat | 6 | 26 |  |  |  |  |  |  |  |  |  |  |  |  |  | 23.08 | [ 8.97; 43.65] 0.2% |
| Lubisi, 2020_South Africa_Pig | 27 | 3984 |  |  |  |  |  |  |  |  |  |  |  |  |  | 0.68 | [ 0.45; 0.98] 0.3% |
| Lubisi, 2020_South Africa_Warthog (Phaecochoerus africanus) | 2 | 107 |  |  |  |  |  |  |  |  |  |  |  |  |  | 1.87 | [ 0.23; 6.59] 0.3% |
| Lwande, 2015_Kenya_Baboon | 0 | 34 |  |  |  |  |  |  |  |  |  |  |  |  |  | 0.00 | [ 0.00; 10.28] 0.3% |
| Lwande, 2015_Kenya_Buffalo | 17 | 95 |  |  |  |  |  |  |  |  |  |  |  |  |  | 17.89 | [10.78; 27.10] 0.3% |
| Lwande, 2015_Kenya_Cattle | 0 | 474 |  |  |  |  |  |  |  |  |  |  |  |  |  | 0.00 | [ 0.00; 0.78] 0.3% |
| Lwande, 2015_Kenya_Elephant | 10 | 45 |  |  |  |  |  |  |  |  |  |  |  |  |  | 22.22 | [11.20; 37.09] 0.3% |
| Lwande, 2015_Kenya_Vervet monkey | 0 | 25 |  |  |  |  |  |  |  |  |  |  |  |  |  | 0.00 | [ 0.00; 13.72] 0.2% |
| Lwande, 2015_Kenya_Warthog (Phaecochoerus africanus) | 5 | 57 |  |  |  |  |  |  |  |  |  |  |  |  |  | 8.77 | [ 2.91; 19.30] 0.3% |
| Lwande, 2015_Kenya_Wildebeest | 1 | 21 |  |  |  |  |  |  |  |  |  |  |  |  |  | 4.76 | [ 0.12; 23.82] 0.2% |
| Lysholm, 2022_ Zambia; Tanzania_Goat | 26 | 950 |  |  |  |  |  |  |  |  |  |  |  |  |  | 2.74 | [ 1.80; 3.98] 0.3% |
| Lysholm, 2022_ Zambia; Tanzania_Sheep | 1 | 27 |  |  |  |  |  |  |  |  |  |  |  |  |  | 3.70 | [ 0.09; 18.97] 0.2% |
| Maganga, 2017_Gabon_Goat | 5 | 106 |  |  |  |  |  |  |  |  |  |  |  |  |  | 4.72 | [ 1.55; 10.67] 0.3% |
| Maganga, 2017_Gabon_Sheep | 8 | 95 |  |  |  |  |  |  |  |  |  |  |  |  |  | 8.42 | [ 3.71; 15.92] 0.3% |
| Magona, 2013_Uganda_Goat | 144 | 1470 |  |  |  |  |  |  |  |  |  |  |  |  |  | 9.80 | [ 8.32; 11.43] 0.3% |
| Mahmoud, 2018_Libya_Cattle | 0 | 171 |  |  |  |  |  |  |  |  |  |  |  |  |  | 0.00 | [ 0.00; 2.13] 0.3% |
| Mahmoud, 2018_Libya_Small ruminants (Goat and Sheep) | 0 | 686 |  |  |  |  |  |  |  |  |  |  |  |  |  | 0.00 | [ 0.00; 0.54] 0.3% |
| Mahmoud, 2021_Egypt_Camel | 19 | 92 |  |  |  |  |  |  |  |  |  |  |  |  |  | 20.65 | [12.92; 30.36] 0.3% |
| Mahmoud, 2021_Egypt_Cattle | 5 | 92 |  |  |  |  |  |  |  |  |  |  |  |  |  | 5.43 | [ 1.79; 12.23] 0.3% |
| Mahmoud, 2021_Egypt_Donkey | 0 | 92 |  |  |  |  |  |  |  |  |  |  |  |  |  | 0.00 | [ 0.00; 3.93] 0.3% |
| Mahmoud, 2021_Egypt_Goat | 13 | 92 |  |  |  |  |  |  |  |  |  |  |  |  |  | 14.13 | [ 7.74; 22.95] 0.3% |
| Mahmoud, 2021_Egypt_Sheep | 60 | 92 |  |  |  |  |  |  |  |  |  |  |  |  |  | 65.22 | [54.57; 74.85] 0.3% |
| Mapaco, 2012_South Africa_Cattle | 35 | 288 |  |  |  |  |  |  |  |  |  |  |  |  |  | 12.15 | [ 8.61; 16.49] 0.3% |
| Mapaco, 2012_South Africa_Sheep | 1 | 73 |  |  |  |  |  |  |  |  |  |  |  |  |  | 1.37 | [ 0.03; 7.40] 0.3% |
| Marietou, 2019_Niger_Cattle | 49 | 160 |  |  |  |  |  |  |  |  |  |  |  |  |  | 30.63 | [23.59; 38.39] 0.3% |
| Marietou, 2019_Niger_Goat | 23 | 125 |  |  |  |  |  |  |  |  |  |  |  |  |  | 18.40 | [12.04; 26.32] 0.3% |
| Marietou, 2019_Niger_Sheep | 41 | 275 |  |  |  |  |  |  |  |  |  |  |  |  |  | 14.91 | [10.92; 19.68] 0.3% |
| Matiko, 2018_Tanzania_Cattle | 104 | 356 |  |  |  |  |  |  |  |  |  |  |  |  |  | 29.21 | [24.54; 34.24] 0.3% |
| Mbotha, 2018_Kenya_Goat | 16 | 182 |  |  |  |  |  |  |  |  |  |  |  |  |  | 8.79 | [ 5.11; 13.88] 0.3% |
| Mbotha, 2018_Kenya_Sheep | 5 | 65 |  |  |  |  |  |  |  |  |  |  |  |  |  | 7.69 | [ 2.54; 17.05] 0.3% |
| Miller, 2011_South Africa_Rhinoceros | 49 | 100 |  |  |  |  |  |  |  |  |  |  |  |  |  | 49.00 | [38.86; 59.20] 0.3% |
| Moiane, 2017_Mozambique_Buffalo | 21 | 69 |  |  |  |  |  |  |  |  |  |  |  |  |  | 30.43 | [19.92; 42.69] 0.3% |
| Moiane, 2017_Mozambique_Cattle | 590 | 1581 |  |  |  |  |  |  |  |  |  |  |  |  |  | 37.32 | [34.93; 39.76] 0.3% |
| Moiane, 2017_Mozambique_Goat | 105 | 1117 |  |  |  |  |  |  |  |  |  |  |  |  |  | 9.40 | [ 7.75; 11.26] 0.3% |
| Moiane, 2017_Mozambique_Sheep | 16 | 85 |  |  |  |  |  |  |  |  |  |  |  |  |  | 18.82 | [11.16; 28.76] 0.3% |
| Mordi, 2020_Ethiopia_Cattle | 28 | 368 |  |  |  |  |  |  |  |  |  |  |  |  |  | 7.61 | [ 5.12; 10.81] 0.3% |
| Mroz, 2017_Egypt_Buffalo | 9 | 173 |  |  |  |  |  |  |  |  |  |  |  |  |  | 5.20 | [ 2.41; 9.65] 0.3% |
| Mroz, 2017_Egypt_Camel | 1 | 131 |  |  |  |  |  |  |  |  |  |  |  |  |  | 0.76 | [ 0.02; 4.18] 0.3% |
| Mroz, 2017_Egypt_Cattle | 533 | 4032 |  |  |  |  |  |  |  |  |  |  |  |  |  | 13.22 | [12.19; 14.30] 0.3% |
| Mroz, 2017_Egypt_Goat | 0 | 26 |  |  |  |  |  |  |  |  |  |  |  |  |  | 0.00 | [ 0.00; 13.23] 0.2% |
| Mroz, 2017_Egypt_Sheep | 3 | 471 |  |  |  |  |  |  |  |  |  |  |  |  |  | 0.64 | [ 0.13; 1.85] 0.3% |
| Munyua, 2010_Kenya_Livestock (goat, sheep, cattle) | 223 | 2849 |  |  |  |  |  |  |  |  |  |  |  |  |  | 7.83 | [ 6.87; 8.88] 0.3% |
| Nabeth, 2001_Mauritania_Camel | 0 | 39 |  |  |  |  |  |  |  |  |  |  |  |  |  | 0.00 | [ 0.00; 9.03] 0.3% |
| Nabeth, 2001_Mauritania_Cattle | 23 | 69 |  |  |  |  |  |  |  |  |  |  |  |  |  | 33.33 | [22.44; 45.71] 0.3% |
| Nabeth, 2001_Mauritania_Goat | 34 | 141 |  |  |  |  |  |  |  |  |  |  |  |  |  | 24.11 | [17.31; 32.03] 0.3% |
| Nabeth, 2001_Mauritania_Rodents | 0 | 73 |  |  |  |  |  |  |  |  |  |  |  |  |  | 0.00 | [ 0.00; 4.93] 0.3% |
| Nabeth, 2001_Mauritania_Sheep | 11 | 89 |  |  |  |  |  |  |  |  |  |  |  |  |  | 12.36 | [ 6.33; 21.04] 0.3% |
| Nakouné, 2016_Central African Republic_Cattle | 57 | 727 |  |  |  |  |  |  |  |  |  |  |  |  |  | 7.84 | [ 5.99; 10.04] 0.3% |
| Nakouné, 2016_Central African Republic_Goat | 11 | 219 |  |  |  |  |  |  |  |  |  |  |  |  |  | 5.02 | [ 2.53; 8.81] 0.3% |
| Nakouné, 2016_Central African Republic_Sheep | 42 | 325 |  |  |  |  |  |  |  |  |  |  |  |  |  | 12.92 | [ 9.47; 17.06] 0.3% |
| Nanyingi, 2017_Kenya_Cattle | 2 | 12 |  |  |  |  |  |  |  |  |  |  |  |  |  | 16.67 | [ 2.09; 48.41] 0.2% |
| Nanyingi, 2017_Kenya_Goat | 70 | 271 |  |  |  |  |  |  |  |  |  |  |  |  |  | 25.83 | [20.72; 31.47] 0.3% |
| Nanyingi, 2017_Kenya_Sheep | 28 | 87 |  |  |  |  |  |  |  |  |  |  |  |  |  | 32.18 | [22.56; 43.06] 0.3% |
| Ndengu, 2020_Zimbabwe_Buffalo | 13 | 111 |  |  |  |  |  |  |  |  |  |  |  |  |  | 11.71 | [ 6.39; 19.19] 0.3% |
| Ndengu, 2020_Zimbabwe_Cattle | 17 | 1011 |  |  |  |  |  |  |  |  |  |  |  |  |  | 1.68 | [ 0.98; 2.68] 0.3% |
| Ndengu, 2020_Zimbabwe_Impala | 0 | 32 |  |  |  |  |  |  |  |  |  |  |  |  |  | 0.00 | [ 0.00; 10.89] 0.3% |
| Ndengu, 2020_Zimbabwe_Kudu | 0 | 18 |  |  |  |  |  |  |  |  |  |  |  |  |  | 0.00 | [ 0.00; 18.53] 0.2% |
| Ndiana, 2019_South Africa_Cattle | 131 | 423 |  |  |  |  |  |  |  |  |  |  |  |  |  | 30.97 | [26.59; 35.62] 0.3% |
| Ndiana, 2019_South Africa_Goat | 33 | 104 |  |  |  |  |  |  |  |  |  |  |  |  |  | 31.73 | [22.95; 41.58] 0.3% |
| Ngoshe, 2020_South Africa_Cattle | 311 | 977 |  |  |  |  |  |  |  |  |  |  |  |  |  | 31.83 | [28.92; 34.86] 0.3% |
| Ngoshe, 2020_South Africa_Goat | 56 | 523 |  |  |  |  |  |  |  |  |  |  |  |  |  | 10.71 | [ 8.19; 13.68] 0.3% |
| Ngoshe, 2020_South Africa_Sheep | 255 | 1549 |  |  |  |  |  |  |  |  |  |  |  |  |  | 16.46 | [14.65; 18.40] 0.3% |
| Odaibo, 2019_Democratic Republic of the Congo_Cattle | 28 | 450 |  |  |  |  |  |  |  |  |  |  |  |  |  | 6.22 | [ 4.17; 8.87] 0.3% |
| Olive, 2013_Madagascar_Eliurus majori | 0 | 15 |  |  |  |  |  |  |  |  |  |  |  |  |  | 0.00 | [ 0.00; 21.80] 0.2% |
| Olive, 2013_Madagascar_Eliurus minor | 0 | 17 |  |  |  |  |  |  |  |  |  |  |  |  |  | 0.00 | [ 0.00; 19.51] 0.2% |
| Olive, 2013_Madagascar_Eliurus tanala | 0 | 15 |  |  |  |  |  |  |  |  |  |  |  |  |  | 0.00 | [ 0.00; 21.80] 0.2% |
| Olive, 2013_Madagascar_Gymnuromys roberti | 0 | 11 |  |  |  |  |  |  |  |  |  |  |  |  |  | 0.00 | [ 0.00; 28.49] 0.2% |
| Olive, 2013_Madagascar_Hemicentetes semispinosus | 0 | 25 |  |  |  |  |  |  |  |  |  |  |  |  |  | 0.00 | [ 0.00; 13.72] 0.2% |
| Olive, 2013_Madagascar_Microgale dobsoni | 0 | 219 |  |  |  |  |  |  |  |  |  |  |  |  |  | 0.00 | [ 0.00; 1.67] 0.3% |
| Olive, 2013_Madagascar_Microgale soricoides | 0 | 61 |  |  |  |  |  |  |  |  |  |  |  |  |  | 0.00 | [ 0.00; 5.87] 0.3% |
| Olive, 2013_Madagascar_Microtus thomasi | 0 | 15 |  |  |  |  |  |  |  |  |  |  |  |  |  | 0.00 | [ 0.00; 21.80] 0.2% |
| Olive, 2013_Madagascar_Nesomys rufus | 0 | 53 |  |  |  |  |  |  |  |  |  |  |  |  |  | 0.00 | [ 0.00; 6.72] 0.3% |
| Olive, 2013_Madagascar_Oryzorictes hova | 0 | 38 |  |  |  |  |  |  |  |  |  |  |  |  |  | 0.00 | [ 0.00; 9.25] 0.3% |
| Olive, 2013_Madagascar_Rattus rattus | 0 | 471 |  |  |  |  |  |  |  |  |  |  |  |  |  | 0.00 | [ 0.00; 0.78] 0.3% |
| Owange, 2014_Kenya_Cattle | 183 | 1396 |  |  |  |  |  |  |  |  |  |  |  |  |  | 13.11 | [11.38; 14.99] 0.3% |
| Oyas, 2018_Kenya_Cattle | 0 | 19 |  |  |  |  |  |  |  |  |  |  |  |  |  | 0.00 | [ 0.00; 17.65] 0.2% |
| Oyas, 2018_Kenya_Goat | 4 | 167 |  |  |  |  |  |  |  |  |  |  |  |  |  | 2.40 | [ 0.66; 6.02] 0.3% |
| Oyas, 2018_Kenya_Sheep | 0 | 332 |  |  |  |  |  |  |  |  |  |  |  |  |  | 0.00 | [ 0.00; 1.10] 0.3% |
| Paweska, 2003_Kenya, Senegal, Somalia, South Africa,Tanzania_Buffalo | 54 | 928 |  |  |  |  |  |  |  |  |  |  |  |  |  | 5.82 | [ 4.40; 7.52] 0.3% |
| Paweska, 2003_Kenya, Senegal, Somalia, South Africa,Tanzania_Cattle | 51 | 203 |  |  |  |  |  |  |  |  |  |  |  |  |  | 25.12 | [19.31; 31.67] 0.3% |
| Paweska, 2003_Kenya, Senegal, Somalia, South Africa,Tanzania_Eland (Taurotragus otyX) | 0 | 14 |  |  |  |  |  |  |  |  |  |  |  |  |  | 0.00 | [ 0.00; 23.16] 0.2% |
| Paweska, 2003_Kenya, Senegal, Somalia, South Africa,Tanzania_Goat | 245 | 636 |  |  |  |  |  |  |  |  |  |  |  |  |  | 38.52 | [34.72; 42.43] 0.3% |
| Paweska, 2003_Kenya, Senegal, Somalia, South Africa,Tanzania_Kudu | 0 | 50 |  |  |  |  |  |  |  |  |  |  |  |  |  | 0.00 | [ 0.00; 7.11] 0.3% |
| Paweska, 2003_Kenya, Senegal, Somalia, South Africa,Tanzania_Sheep | 92 | 1159 |  |  |  |  |  |  |  |  |  |  |  |  |  | 7.94 | [ 6.45; 9.65] 0.3% |
| Paweska, 2003_Kenya, Senegal, Somalia, South Africa,Tanzania_Wildebeest | 0 | 65 |  |  |  |  |  |  |  |  |  |  |  |  |  | 0.00 | [ 0.00; 5.52] 0.3% |
| Paweska, 2003_Kenya, Somalia, South Africa, Tanzania, Uganda_Cattle | 82 | 997 |  |  |  |  |  |  |  |  |  |  |  |  |  | 8.22 | [ 6.59; 10.11] 0.3% |
| Paweska, 2003_Kenya, Somalia, South Africa, Tanzania, Uganda_Goat | 357 | 1459 |  |  |  |  |  |  |  |  |  |  |  |  |  | 24.47 | [22.28; 26.76] 0.3% |
| Paweska, 2003_Kenya, Somalia, South Africa, Tanzania, Uganda_Sheep | 105 | 1321 |  |  |  |  |  |  |  |  |  |  |  |  |  | 7.95 | [ 6.55; 9.54] 0.3% |
| Paweska, 2005_Ethiopia_Camel | 2 | 51 |  |  |  |  |  |  |  |  |  |  |  |  |  | 3.92 | [ 0.48; 13.46] 0.3% |
| Paweska, 2005_Ethiopia_Cattle | 0 | 21 |  |  |  |  |  |  |  |  |  |  |  |  |  | 0.00 | [ 0.00; 16.11] 0.2% |
| Paweska, 2005_Ethiopia_Sheep | 2 | 34 |  |  |  |  |  |  |  |  |  |  |  |  |  | 5.88 | [ 0.72; 19.68] 0.3% |
| Paweska, 2005_Kenya_Camel | 4 | 11 |  |  |  |  |  |  |  |  |  |  |  |  |  | 36.36 | [10.93; 69.21] 0.2% |
| Paweska, 2005_Kenya_Cattle | 45 | 77 |  |  |  |  |  |  |  |  |  |  |  |  |  | 58.44 | [46.64; 69.57] 0.3% |
| Paweska, 2005_Kenya_Goat | 184 | 426 |  |  |  |  |  |  |  |  |  |  |  |  |  | 43.19 | [38.43; 48.05] 0.3% |
| Paweska, 2005_Kenya_Sheep | 43 | 165 |  |  |  |  |  |  |  |  |  |  |  |  |  | 26.06 | [19.55; 33.46] 0.3% |
| Paweska, 2005_Somalia_Camel | 25 | 94 |  |  |  |  |  |  |  |  |  |  |  |  |  | 26.60 | [18.01; 36.71] 0.3% |
| Paweska, 2005_Somalia_Cattle | 3 | 12 |  |  |  |  |  |  |  |  |  |  |  |  |  | 25.00 | [ 5.49; 57.19] 0.2% |
| Paweska, 2005_Somalia_Goat | 19 | 39 |  |  |  |  |  |  |  |  |  |  |  |  |  | 48.72 | [32.42; 65.22] 0.3% |
| Paweska, 2005_Somalia_Sheep | 8 | 33 |  |  |  |  |  |  |  |  |  |  |  |  |  | 24.24 | [11.09; 42.26] 0.3% |
| Paweska, 2005_South Africa_Buffalo | 53 | 258 |  |  |  |  |  |  |  |  |  |  |  |  |  | 20.54 | [15.78; 25.99] 0.3% |
| Paweska, 2005_South Africa_Cattle | 0 | 537 |  |  |  |  |  |  |  |  |  |  |  |  |  | 0.00 | [ 0.00; 0.68] 0.3% |
| Paweska, 2005_South Africa_Sheep | 0 | 198 |  |  |  |  |  |  |  |  |  |  |  |  |  | 0.00 | [ 0.00; 1.85] 0.3% |
| Paweska, 2005_Tanzania_Cattle | 11 | 47 |  |  |  |  |  |  |  |  |  |  |  |  |  | 23.40 | [12.30; 38.03] 0.3% |
| Paweska, 2005_Tanzania_Goat | 20 | 96 |  |  |  |  |  |  |  |  |  |  |  |  |  | 20.83 | [13.21; 30.33] 0.3% |
| Paweska, 2005_Tanzania_Sheep | 12 | 58 |  |  |  |  |  |  |  |  |  |  |  |  |  | 20.69 | [11.17; 33.35] 0.3% |
| Paweska, 2005_Uganda_Goat | 9 | 237 |  |  |  |  |  |  |  |  |  |  |  |  |  | 3.80 | [ 1.75; 7.09] 0.3% |
| Paweska, 2008_Kenya, South Africa_Buffalo | 77 | 1023 |  |  |  |  |  |  |  |  |  |  |  |  |  | 7.53 | [ 5.99; 9.32] 0.3% |
| Peterson, 2017_Madagascar_Cattle | 91 | 1140 |  |  |  |  |  |  |  |  |  |  |  |  |  | 7.98 | [ 6.48; 9.71] 0.3% |
| Poueme, 2019_Cameroon_Goat | 8 | 355 |  |  |  |  |  |  |  |  |  |  |  |  |  | 2.25 | [ 0.98; 4.39] 0.3% |
| Poueme, 2019_Cameroon_Sheep | 15 | 325 |  |  |  |  |  |  |  |  |  |  |  |  |  | 4.62 | [ 2.61; 7.50] 0.3% |
| Ringot, 2004_Chad_Cattle | 5 | 114 |  |  |  |  |  |  |  |  |  |  |  |  |  | 4.39 | [ 1.44; 9.94] 0.3% |
| Ringot, 2004_Chad_Goat | 12 | 139 |  |  |  |  |  |  |  |  |  |  |  |  |  | 8.63 | [ 4.54; 14.59] 0.3% |
| Ringot, 2004_Chad_Sheep | 32 | 300 |  |  |  |  |  |  |  |  |  |  |  |  |  | 10.67 | [ 7.41; 14.72] 0.3% |
| Rissmann, 2017_Cameroon_Cattle | 118 | 1005 |  |  |  |  |  |  |  |  |  |  |  |  |  | 11.74 | [ 9.82; 13.89] 0.3% |
| Rissmann, 2017_Cameroon_Small ruminants (Goat and Sheep) | 30 | 917 |  |  |  |  |  |  |  |  |  |  |  |  |  | 3.27 | [ 2.22; 4.64] 0.3% |
| Rissmann, 2017_Mauritania_Camel | 24 | 79 |  |  |  |  |  |  |  |  |  |  |  |  |  | 30.38 | [20.53; 41.75] 0.3% |
| Rissmann, 2017_Mauritania_Cattle | 73 | 484 |  |  |  |  |  |  |  |  |  |  |  |  |  | 15.08 | [12.01; 18.59] 0.3% |
| Rissmann, 2017_Mauritania_Small ruminants (Goat and Sheep) | 29 | 497 |  |  |  |  |  |  |  |  |  |  |  |  |  | 5.84 | [ 3.94; 8.27] 0.3% |
| Roger, 2011_Comoros_Cattle | 60 | 196 |  |  |  |  |  |  |  |  |  |  |  |  |  | 30.61 | [24.24; 37.58] 0.3% |
| Roger, 2011_Comoros_Goat | 84 | 251 |  |  |  |  |  |  |  |  |  |  |  |  |  | 33.47 | [27.66; 39.67] 0.3% |
| Roger, 2011_Comoros_Sheep | 16 | 41 |  |  |  |  |  |  |  |  |  |  |  |  |  | 39.02 | [24.20; 55.50] 0.3% |
| Roger, 2014_Comoros_Livestock (goat, sheep, cattle) | 76 | 275 |  |  |  |  |  |  |  |  |  |  |  |  |  | 27.64 | [22.44; 33.33] 0.3% |
| Rostal, 2010_Kenya_Goat | 2 | 75 |  |  |  |  |  |  |  |  |  |  |  |  |  | 2.67 | [ 0.32; 9.30] 0.3% |
| Rostal, 2010_Kenya_Sheep | 34 | 188 |  |  |  |  |  |  |  |  |  |  |  |  |  | 18.09 | [12.86; 24.34] 0.3% |
| Roug, 2020_Tanzania_Buffalo | 2 | 40 |  |  |  |  |  |  |  |  |  |  |  |  |  | 5.00 | [ 0.61; 16.92] 0.3% |
| Salekwa, 2019_Tanzania_Cattle | 34 | 443 |  |  |  |  |  |  |  |  |  |  |  |  |  | 7.67 | [ 5.37; 10.56] 0.3% |
| Selmi, 2020_Tunisia_Camel | 162 | 470 |  |  |  |  |  |  |  |  |  |  |  |  |  | 34.47 | [30.17; 38.96] 0.3% |
| Sindato, 2013_Tanzania_Buffalo | 9 | 22 |  |  |  |  |  |  |  |  |  |  |  |  |  | 40.91 | [20.71; 63.65] 0.2% |
| Sindato, 2013_Tanzania_Cattle | 11 | 93 |  |  |  |  |  |  |  |  |  |  |  |  |  | 11.83 | [ 6.05; 20.18] 0.3% |
| Sindato, 2013_Tanzania_Goat | 8 | 95 |  |  |  |  |  |  |  |  |  |  |  |  |  | 8.42 | [ 3.71; 15.92] 0.3% |
| Sindato, 2013_Tanzania_Lion | 0 | 18 |  |  |  |  |  |  |  |  |  |  |  |  |  | 0.00 | [ 0.00; 18.53] 0.2% |
| Sindato, 2013_Tanzania_Sheep | 11 | 97 |  |  |  |  |  |  |  |  |  |  |  |  |  | 11.34 | [ 5.80; 19.39] 0.3% |
| Sindato, 2013_Tanzania_Thomson Gazelle | 0 | 11 |  |  |  |  |  |  |  |  |  |  |  |  |  | 0.00 | [ 0.00; 28.49] 0.2% |
| Sindato, 2013_Tanzania_Wildebeest | 0 | 13 |  |  |  |  |  |  |  |  |  |  |  |  |  | 0.00 | [ 0.00; 24.71] 0.2% |
| Sindato, 2013_Tanzania_Zebra | 0 | 24 |  |  |  |  |  |  |  |  |  |  |  |  |  | 0.00 | [ 0.00; 14.25] 0.2% |
| Sindato, 2015_Tanzania_Cattle | 210 | 756 |  |  |  |  |  |  |  |  |  |  |  |  |  | 27.78 | [24.61; 31.12] 0.3% |
| Sindato, 2015_Tanzania_Goat | 116 | 531 |  |  |  |  |  |  |  |  |  |  |  |  |  | 21.85 | [18.40; 25.61] 0.3% |
| Sindato, 2015_Tanzania_Sheep | 44 | 148 |  |  |  |  |  |  |  |  |  |  |  |  |  | 29.73 | [22.50; 37.79] 0.3% |
| Soumare, 2007_Somalia_Animal unspecified | 411 | 9517 |  |  |  |  |  |  |  |  |  |  |  |  |  | 4.32 | [ 3.92; 4.75] 0.3% |
| Soumare, 2007_Somalia_Cattle | 0 | 31 |  |  |  |  |  |  |  |  |  |  |  |  |  | 0.00 | [ 0.00; 11.22] 0.3% |
| Sow, 2016_Senegal_Livestock (goat, sheep, cattle) | 55 | 137 |  |  |  |  |  |  |  |  |  |  |  |  |  | 40.15 | [31.87; 48.86] 0.3% |
| Spiropoulou, 2018_Uganda_Cattle | 86 | 324 |  |  |  |  |  |  |  |  |  |  |  |  |  | 26.54 | [21.81; 31.71] 0.3% |
| Spiropoulou, 2018_Uganda_Goat | 40 | 569 |  |  |  |  |  |  |  |  |  |  |  |  |  | 7.03 | [ 5.07; 9.45] 0.3% |
| Spiropoulou, 2018_Uganda_Sheep | 7 | 158 |  |  |  |  |  |  |  |  |  |  |  |  |  | 4.43 | [ 1.80; 8.92] 0.3% |
| Sternberg Lewerin, 2018_Uganda_Cattle | 9 | 456 |  |  |  |  |  |  |  |  |  |  |  |  |  | 1.97 | [ 0.91; 3.71] 0.3% |
| Sumaye, 2013_Tanzania_Cattle | 107 | 970 |  |  |  |  |  |  |  |  |  |  |  |  |  | 11.03 | [ 9.13; 13.17] 0.3% |
| Sumaye, 2013_Tanzania_Goat | 54 | 455 |  |  |  |  |  |  |  |  |  |  |  |  |  | 11.87 | [ 9.04; 15.20] 0.3% |
| Sumaye, 2013_Tanzania_Sheep | 29 | 255 |  |  |  |  |  |  |  |  |  |  |  |  |  | 11.37 | [ 7.75; 15.92] 0.3% |
| Swai, 2015_Tanzania_Camel | 30 | 109 |  |  |  |  |  |  |  |  |  |  |  |  |  | 27.52 | [19.40; 36.90] 0.3% |
| Troupin, 2022_Guinea_Cattle | 76 | 463 |  |  |  |  |  |  |  |  |  |  |  |  |  | 16.41 | [13.16; 20.11] 0.3% |
| Troupin, 2022_Guinea_Goat | 4 | 408 |  |  |  |  |  |  |  |  |  |  |  |  |  | 0.98 | [ 0.27; 2.49] 0.3% |
| Troupin, 2022_Guinea_Sheep | 5 | 486 |  |  |  |  |  |  |  |  |  |  |  |  |  | 1.03 | [ 0.33; 2.38] 0.3% |
| Tshilenge, 2019_Democratic Republic of the Congo_Goat | 38 | 672 |  |  |  |  |  |  |  |  |  |  |  |  |  | 5.65 | [ 4.03; 7.68] 0.3% |
| Tshilenge, 2019_Democratic Republic of the Congo_Sheep | 16 | 221 |  |  |  |  |  |  |  |  |  |  |  |  |  | 7.24 | [ 4.19; 11.49] 0.3% |
| Umuhoza, 2017_Rwanda_Cattle | 100 | 595 |  |  |  |  |  |  |  |  |  |  |  |  |  | 16.81 | [13.89; 20.06] 0.3% |
| Van den Bergh, 2020_South Africa_Impala | 17 | 37 |  |  |  |  |  |  |  |  |  |  |  |  |  | 45.95 | [29.49; 63.08] 0.3% |
| Van den Bergh, 2020_South Africa_Nyala | 97 | 289 |  |  |  |  |  |  |  |  |  |  |  |  |  | 33.56 | [28.14; 39.33] 0.3% |
| Wensman, 2015_Tanzania_Small ruminants (Goat and Sheep) | 29 | 354 |  |  |  |  |  |  |  |  |  |  |  |  |  | 8.19 | [ 5.55; 11.55] 0.3% |
| Youssef, 2001_Egypt_Rattus rattus | 88 | 300 |  |  |  |  |  |  |  |  |  |  |  |  |  | 29.33 | [24.24; 34.84] 0.3% |
| Youssef, 2009_Egypt_Pig | 37 | 245 |  |  |  |  |  |  |  |  |  |  |  |  |  | 15.10 | [10.86; 20.21] 0.3% |
| Zouaghi, 2021_Tunisia_Cattle | 10 | 299 |  |  |  |  |  |  |  |  |  |  |  |  |  | 3.34 | [ 1.62; 6.06] 0.3% |
| Zouaghi, 2021_Tunisia_Goat | 0 | 165 |  |  |  |  |  |  |  |  |  |  |  |  |  | 0.00 | [ 0.00; 2.21] 0.3% |
| Zouaghi, 2021_Tunisia_Sheep | 6 | 235 |  |  |  |  |  |  |  |  |  |  |  |  |  | 2.55 | [ 0.94; 5.47] 0.3% |
| **Random effect meta−analysis** |  | **102633** |  |  |  |  |  |  |  |  |  |  |  |  |  | **10.57** | **[ 9.22; 11.99] 78.9%** |

**Prediction interval**

Heterogeneity: *I* 2 = 97.8% [97.7%; 97.9%], τ2 = 0.0303, *p* = 0

| **Animal_prevalence_Recent infection** |  | | | | | | | | | | | | | |  |
| --- | --- | --- | --- | --- | --- | --- | --- | --- | --- | --- | --- | --- | --- | --- | --- |
| Alhaji, 2020_Nigeria_Cattle | 6 | 107 |  |  |  |  |  |  |  |  |  |  |  | 5.61 | [ 2.09; 11.81] 0.3% |
| Capobianco Dondona, 2016_Namibia_Springbok | 30 | 200 |  |  |  |  |  |  |  |  |  |  |  | 15.00 | [10.35; 20.72] 0.3% |
| Capobianco Dondona, 2016_Namibia_Wildebeest | 0 | 50 |  |  |  |  |  |  |  |  |  |  |  | 0.00 | [ 0.00; 7.11] 0.3% |
| Chevalier, 2011_Madagascar_Cattle | 7 | 894 |  |  |  |  |  |  |  |  |  |  |  | 0.78 | [ 0.32; 1.61] 0.3% |
| Cichon, 2021_Mauritania_Small ruminants (Goat and Sheep) | 22 | 458 |  |  |  |  |  |  |  |  |  |  |  | 4.80 | [ 3.03; 7.18] 0.3% |
| El Mamy, 2011_Mauritania_Camel | 12 | 279 |  |  |  |  |  |  |  |  |  |  |  | 4.30 | [ 2.24; 7.39] 0.3% |
| El Mamy, 2011_Mauritania_Small ruminants (Goat and Sheep) | 111 | 262 |  |  |  |  |  |  |  |  |  |  |  | 42.37 | [36.31; 48.60] 0.3% |
| Fafetine, 2014_Mozambique_Small ruminants (Goat and Sheep) | 31 | 127 |  |  |  |  |  |  |  |  |  |  |  | 24.41 | [17.23; 32.82] 0.3% |
| Faye, 2007_Mauritania_Goat | 28 | 48 |  |  |  |  |  |  |  |  |  |  |  | 58.33 | [43.21; 72.39] 0.3% |
| Faye, 2007_Mauritania_Goat | 18 | 78 |  |  |  |  |  |  |  |  |  |  |  | 23.08 | [14.29; 34.00] 0.3% |
| Faye, 2007_Mauritania_Sheep | 5 | 13 |  |  |  |  |  |  |  |  |  |  |  | 38.46 | [13.86; 68.42] 0.2% |
| Georges, 2018_Democratic Republic of the Congo_Cattle | 8 | 450 |  |  |  |  |  |  |  |  |  |  |  | 1.78 | [ 0.77; 3.47] 0.3% |
| Hanafi, 2011_Egypt_Cattle | 5 | 48 |  |  |  |  |  |  |  |  |  |  |  | 10.42 | [ 3.47; 22.66] 0.3% |
| Hanafi, 2011_Egypt_Sheep | 2 | 36 |  |  |  |  |  |  |  |  |  |  |  | 5.56 | [ 0.68; 18.66] 0.3% |
| Hassan, 2020_Kenya_Camel | 2 | 22 |  |  |  |  |  |  |  |  |  |  |  | 9.09 | [ 1.12; 29.16] 0.2% |
| Hassan, 2020_Kenya_Goat | 10 | 22 |  |  |  |  |  |  |  |  |  |  |  | 45.45 | [24.39; 67.79] 0.2% |
| Hassan, 2020_Kenya_Sheep | 10 | 22 |  |  |  |  |  |  |  |  |  |  |  | 45.45 | [24.39; 67.79] 0.2% |
| Jäckel, 2013_Mauritania_Small ruminants (Goat and Sheep) | 49 | 93 |  |  |  |  |  |  |  |  |  |  |  | 52.69 | [42.06; 63.14] 0.3% |
| Jeanmaire, 2011_Madagascar_Cattle | 9 | 3437 |  |  |  |  |  |  |  |  |  |  |  | 0.26 | [ 0.12; 0.50] 0.3% |
| Jeanmaire, 2011_Madagascar_Small ruminants (Goat and Sheep) | 33 | 989 |  |  |  |  |  |  |  |  |  |  |  | 3.34 | [ 2.31; 4.65] 0.3% |
| Mapaco, 2012_South Africa_Cattle | 16 | 288 |  |  |  |  |  |  |  |  |  |  |  | 5.56 | [ 3.21; 8.87] 0.3% |
| Mapaco, 2012_South Africa_Sheep | 1 | 73 |  |  |  |  |  |  |  |  |  |  |  | 1.37 | [ 0.03; 7.40] 0.3% |
| Matiko, 2018_Tanzania_Cattle | 30 | 356 |  |  |  |  |  |  |  |  |  |  |  | 8.43 | [ 5.76; 11.81] 0.3% |
| Munyua, 2010_Kenya_Livestock (goat, sheep, cattle) | 126 | 2296 |  |  |  |  |  |  |  |  |  |  |  | 5.49 | [ 4.59; 6.50] 0.3% |
| Nabeth, 2001_Mauritania_Camel | 1 | 39 |  |  |  |  |  |  |  |  |  |  |  | 2.56 | [ 0.06; 13.48] 0.3% |
| Nabeth, 2001_Mauritania_Cattle | 1 | 69 |  |  |  |  |  |  |  |  |  |  |  | 1.45 | [ 0.04; 7.81] 0.3% |
| Nabeth, 2001_Mauritania_Goat | 23 | 141 |  |  |  |  |  |  |  |  |  |  |  | 16.31 | [10.63; 23.46] 0.3% |
| Nabeth, 2001_Mauritania_Rodents | 0 | 73 |  |  |  |  |  |  |  |  |  |  |  | 0.00 | [ 0.00; 4.93] 0.3% |
| Nabeth, 2001_Mauritania_Sheep | 31 | 89 |  |  |  |  |  |  |  |  |  |  |  | 34.83 | [25.04; 45.67] 0.3% |
| Nakouné, 2016_Central African Republic_Cattle | 8 | 727 |  |  |  |  |  |  |  |  |  |  |  | 1.10 | [ 0.48; 2.16] 0.3% |
| Nakouné, 2016_Central African Republic_Goat | 3 | 219 |  |  |  |  |  |  |  |  |  |  |  | 1.37 | [ 0.28; 3.95] 0.3% |
| Nakouné, 2016_Central African Republic_Sheep | 14 | 325 |  |  |  |  |  |  |  |  |  |  |  | 4.31 | [ 2.37; 7.12] 0.3% |
| Odaibo, 2019_Democratic Republic of the Congo_Cattle | 8 | 450 |  |  |  |  |  |  |  |  |  |  |  | 1.78 | [ 0.77; 3.47] 0.3% |
| Opayele, 2019_Nigeria_Cattle | 1 | 244 |  |  |  |  |  |  |  |  |  |  |  | 0.41 | [ 0.01; 2.26] 0.3% |
| Opayele, 2019_Nigeria_Goat | 1 | 44 |  |  |  |  |  |  |  |  |  |  |  | 2.27 | [ 0.06; 12.02] 0.3% |
| Owange, 2014_Kenya_Cattle | 20 | 1396 |  |  |  |  |  |  |  |  |  |  |  | 1.43 | [ 0.88; 2.20] 0.3% |
| Roug, 2020_Tanzania_Buffalo | 1 | 40 |  |  |  |  |  |  |  |  |  |  |  | 2.50 | [ 0.06; 13.16] 0.3% |
| Salekwa, 2019_Tanzania_Cattle | 1 | 443 |  |  |  |  |  |  |  |  |  |  |  | 0.23 | [ 0.01; 1.25] 0.3% |
| Soumare, 2007_Somalia_Camel | 0 | 218 |  |  |  |  |  |  |  |  |  |  |  | 0.00 | [ 0.00; 1.68] 0.3% |
| Soumare, 2007_Somalia_Goat | 0 | 5527 |  |  |  |  |  |  |  |  |  |  |  | 0.00 | [ 0.00; 0.07] 0.3% |
| Soumare, 2007_Somalia_Sheep | 0 | 3741 |  |  |  |  |  |  |  |  |  |  |  | 0.00 | [ 0.00; 0.10] 0.3% |
| Sow, 2016_Senegal_Cattle | 0 | 14 |  |  |  |  |  |  |  |  |  |  |  | 0.00 | [ 0.00; 23.16] 0.2% |
| Sow, 2016_Senegal_Goat | 0 | 49 |  |  |  |  |  |  |  |  |  |  |  | 0.00 | [ 0.00; 7.25] 0.3% |
| Sow, 2016_Senegal_Sheep | 0 | 74 |  |  |  |  |  |  |  |  |  |  |  | 0.00 | [ 0.00; 4.86] 0.3% |
| Stoek, 2022_Mauritania_Cattle | 9 | 44 |  |  |  |  |  |  |  |  |  |  |  | 20.45 | [ 9.80; 35.30] 0.3% |
| Sumaye, 2013_Tanzania_Sheep | 9 | 1680 |  |  |  |  |  |  |  |  |  |  |  | 0.54 | [ 0.25; 1.01] 0.3% |
| **Random effect meta−analysis** |  | **26294** |  |  |  |  |  |  |  |  |  |  |  | **5.97** | **[ 3.96; 8.32] 13.3%** |

**Prediction interval**

Heterogeneity: *I* 2 = 97.7% [97.3%; 98.0%], τ2 = 0.0198, *p* = 0

**. . [ 0.00; 20.26] −−**

**. . [ 0.00; 39.74] −−**

**. . [ 0.00; 26.69] −−**


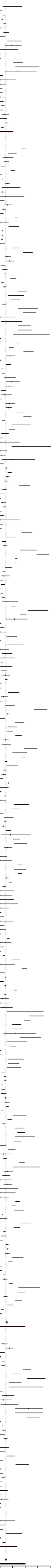


**Overall random effect meta−analysis Prediction interval**

Heterogeneity: *I* 2 = 98.2% [98.1%; 98.2%], τ2 = 0.0347, *p* = 0

Test for subgroup differences: χ2 = 26.30, df = 2 (*p* < 0.0001)

2

# S4 Fig. Pooled prevalence estimates of Rift Valley fever virus infections in other animal species in Africa.

**134274**

0 20 40 60 80

**9.31**

**[ 8.11; 10.59] 100.0%**

**[ 0.00; 40.07]**
