# Supplementary material for: Contemporary epidemiological data of Rift Valley fever virus in humans, mosquitoes and other animal species in Africa: A systematic review and meta‐analysis
Source: Vet Med Sci. 2023 Aug 7;9(5):2309–28. doi: 10.1002/vms3.1238 (PMC10508527; doi:10.1002/vms3.1238)
Supplement: Supplementary file 6 — Supporting Information [file VMS3-9-2309-s003.docx]

**Supplemental Material (Tables)**

**Contemporary epidemiological data of Rift Valley fever virus in humans, mosquitoes, and other animal species in Africa: a systematic review and meta-analysis.**

**Table of content**

[S1 Table. Preferred reporting items for systematic reviews and meta-analyses checklist 2](#_Toc130927942)

[S2 Table: Search strategy in databases 5](#_Toc130927943)

[S3 Table. Individual characteristics of included studies 6](#_Toc130927944)

[S4 Table. Risk of bias assessment 17](#_Toc130927945)

[S5 Table. Subgroup analyses of case fatality rate of Rift Valley fever in humans in Africa. 35](#_Toc130927946)

[S6 Table. Subgroup analyses of prevalence of Rift Valley fever in humans in Africa. 36](#_Toc130927947)

[S7 Table. Subgroup analyses of prevalence of Rift Valley fever in other animal species in Africa. 38](#_Toc130927948)

# S1 Table. Preferred reporting items for systematic reviews and meta-analyses checklist

| **Section/topic** | **#** | **Checklist item** | **Reported on page #** |
| --- | --- | --- | --- |
| **TITLE** | | |  |
| Title | 1 | Identify the report as a systematic review, meta-analysis, or both. | 1 |
| **ABSTRACT** | | |  |
| Structured summary | 2 | Provide a structured summary including, as applicable: background; objectives; data sources; study eligibility criteria, participants, and interventions; study appraisal and synthesis methods; results; limitations; conclusions and implications of key findings; systematic review registration number. | 2 |
| **INTRODUCTION** | | |  |
| Rationale | 3 | Describe the rationale for the review in the context of what is already known. | 7-8 |
| Objectives | 4 | Provide an explicit statement of questions being addressed with reference to participants, interventions, comparisons, outcomes, and study design (PICOS). | 8 |
| **METHODS** | | |  |
| Protocol and registration | 5 | Indicate if a review protocol exists, if and where it can be accessed (e.g., Web address), and, if available, provide registration information including registration number. | 9 |
| Eligibility criteria | 6 | Specify study characteristics (e.g., PICOS, length of follow-up) and report characteristics (e.g., years considered, language, publication status) used as criteria for eligibility, giving rationale. | 9 |
| Information sources | 7 | Describe all information sources (e.g., databases with dates of coverage, contact with study authors to identify additional studies) in the search and date last searched. | 9-10 |
| Search | 8 | Present full electronic search strategy for at least one database, including any limits used, such that it could be repeated. | Appendix |
| Study selection | 9 | State the process for selecting studies (i.e., screening, eligibility, included in systematic review, and, if applicable, included in the meta-analysis). | 10 |
| Data collection process | 10 | Describe method of data extraction from reports (e.g., piloted forms, independently, in duplicate) and any processes for obtaining and confirming data from investigators. | 10 |
| Data items | 11 | List and define all variables for which data were sought (e.g., PICOS, funding sources) and any assumptions and simplifications made. | 10 |
| Risk of bias in individual studies | 12 | Describe methods used for assessing risk of bias of individual studies (including specification of whether this was done at the study or outcome level), and how this information is to be used in any data synthesis. | 11 |
| Summary measures | 13 | State the principal summary measures (e.g., risk ratio, difference in means). | 11-12 |
| Synthesis of results | 14 | Describe the methods of handling data and combining results of studies, if done, including measures of consistency (e.g., I^2^) for each meta-analysis. | 11-12 |

| **Section/topic** | **#** | **Checklist item** | **Reported on page #** |
| --- | --- | --- | --- |
| Risk of bias across studies | 15 | Specify any assessment of risk of bias that may affect the cumulative evidence (e.g., publication bias, selective reporting within studies). | 12 |
| Additional analyses | 16 | Describe methods of additional analyses (e.g., sensitivity or subgroup analyses, meta-regression), if done, indicating which were pre-specified. | 12 |
| **RESULTS** | | |  |
| Study selection | 17 | Give numbers of studies screened, assessed for eligibility, and included in the review, with reasons for exclusions at each stage, ideally with a flow diagram. | 12 |
| Study characteristics | 18 | For each study, present characteristics for which data were extracted (e.g., study size, PICOS, follow-up period) and provide the citations. | 13 |
| Risk of bias within studies | 19 | Present data on risk of bias of each study and, if available, any outcome level assessment (see item 12). | 13 |
| Results of individual studies | 20 | For all outcomes considered (benefits or harms), present, for each study: (a) simple summary data for each intervention group (b) effect estimates and confidence intervals, ideally with a forest plot. | 13 |
| Synthesis of results | 21 | Present results of each meta-analysis done, including confidence intervals and measures of consistency. | 13-16 |
| Risk of bias across studies | 22 | Present results of any assessment of risk of bias across studies (see Item 15). | 13 |
| Additional analysis | 23 | Give results of additional analyses, if done (e.g., sensitivity or subgroup analyses, meta-regression [see Item 16]). | 13-16 |
| **DISCUSSION** | | |  |
| Summary of evidence | 24 | Summarize the main findings including the strength of evidence for each main outcome; consider their relevance to key groups (e.g., healthcare providers, users, and policy makers). | 17 |
| Limitations | 25 | Discuss limitations at study and outcome level (e.g., risk of bias), and at review-level (e.g., incomplete retrieval of identified research, reporting bias). | 19-20 |
| Conclusions | 26 | Provide a general interpretation of the results in the context of other evidence, and implications for future research. | 20 |
| **FUNDING** | | |  |
| Funding | 27 | Describe sources of funding for the systematic review and other support (e.g., supply of data); role of funders for the systematic review. | 13 |

# S2 Table: Search strategy in databases

| Database | Search | Items |
| --- | --- | --- |
| PubMed | (RVFV OR RVF OR Rift Valley fever virus OR Rift Valley Fever) AND (Africa* OR Algeria OR Angola OR Benin OR Botswana OR "Burkina Faso" OR Burundi OR Cameroon OR "Canary Islands" OR "Cape Verde" OR "Central African Republic" OR Chad OR Comoros OR Congo OR "Democratic Republic of Congo" OR Djibouti OR Egypt OR "Equatorial Guinea" OR Eritrea OR Ethiopia OR Gabon OR Gambia OR Ghana OR Guinea OR "Guinea Bissau" OR "Ivory Coast" OR "Cote d'Ivoire" OR Jamahiriya OR Kenya OR Lesotho OR Liberia OR Libya OR Madagascar OR Malawi OR Mali OR Mauritania OR Mauritius OR Mayotte OR Morocco OR Mozambique OR Namibia OR Niger OR Nigeria OR Principe OR Reunion OR Rwanda OR "Sao Tome" OR Senegal OR Seychelles OR "Sierra Leone" OR Somalia OR "South Africa" OR "South Sudan" OR "St Helena" OR Sudan OR Swaziland OR Tanzania OR Togo OR Tunisia OR Uganda OR "Western Sahara" OR Zaire OR Zambia OR Zimbabwe OR "Central Africa" OR "Central African" OR "West Africa" OR "West African" OR "Western Africa" OR "Western African" OR "East Africa" OR "East African" OR "Eastern Africa" OR "Eastern African" OR "North Africa" OR "North African" OR "Northern Africa" OR "Northern African" OR "South African" OR "Southern Africa" OR "Southern African" OR "sub Saharan Africa" OR "sub Saharan African" OR "sub Saharan Africa" OR "sub Saharan African") | 1429 |
|  |  |  |
| Web of Science | (RVFV OR RVF OR Rift Valley fever virus OR Rift Valley Fever) AND (Africa* OR Algeria OR Angola OR Benin OR Botswana OR "Burkina Faso" OR Burundi OR Cameroon OR "Canary Islands" OR "Cape Verde" OR "Central African Republic" OR Chad OR Comoros OR Congo OR "Democratic Republic of Congo" OR Djibouti OR Egypt OR "Equatorial Guinea" OR Eritrea OR Ethiopia OR Gabon OR Gambia OR Ghana OR Guinea OR "Guinea Bissau" OR "Ivory Coast" OR "Cote d'Ivoire" OR Jamahiriya OR Kenya OR Lesotho OR Liberia OR Libya OR Madagascar OR Malawi OR Mali OR Mauritania OR Mauritius OR Mayotte OR Morocco OR Mozambique OR Namibia OR Niger OR Nigeria OR Principe OR Reunion OR Rwanda OR "Sao Tome" OR Senegal OR Seychelles OR "Sierra Leone" OR Somalia OR "South Africa" OR "South Sudan" OR "St Helena" OR Sudan OR Swaziland OR Tanzania OR Togo OR Tunisia OR Uganda OR "Western Sahara" OR Zaire OR Zambia OR Zimbabwe OR "Central Africa" OR "Central African" OR "West Africa" OR "West African" OR "Western Africa" OR "Western African" OR "East Africa" OR "East African" OR "Eastern Africa" OR "Eastern African" OR "North Africa" OR "North African" OR "Northern Africa" OR "Northern African" OR "South African" OR "Southern Africa" OR "Southern African" OR "sub Saharan Africa" OR "sub Saharan African" OR "sub Saharan Africa" OR "sub Saharan African") | 1868 |
|  |  |  |
| African Index Medicus | RVFV OR RVF OR Rift Valley fever virus OR Rift Valley Fever | 10 |
|  |  |  |
| African Journal Online | Rift Valley Fever | 41 |

# S3 Table. Individual characteristics of included studies

| **N°** | **Author** | **Year of publication** | **Study Design** | **Sampling** | **Sampling method** | **Number of sites** | **Timing of data collection** | **Countries** | **Study period** | **Age range** | **Recrutment setting** | **Setting** | **Hospitalization** | **Population characteristics** | **Study population: Other animal species** | **Type of animal** | **Other animal species orders** | **Detection assay** | **Target detected** | **Sample types** |
| --- | --- | --- | --- | --- | --- | --- | --- | --- | --- | --- | --- | --- | --- | --- | --- | --- | --- | --- | --- | --- |
| 1 | Abakar | 2014 | Cross-sectional | Probabilistic | Multistage sampling | Multicenter | Prospetively | Chad | Unclear/ Not reported | Not applicable | Rural | Community-based | Not applicable | Other animal species | Cattle, Sheep, Goat | Domesticated animal | Artiodactyla | Indirect ELISA | Antibodies | Serum |
| 2 | Abdallah | 2016 | Cross-sectional | Probabilistic | Multistage sampling | Monocenter | Prospetively | Sudan | Oct/2014-Mar/2015 | Not applicable | Urban | Community-based | Not applicable | Other animal species | Camel | Domesticated animal | Artiodactyla | Indirect ELISA | IgG | Serum |
| 3 | Adamu | 2020 | Cross-sectional | Probabilistic | Simple random sampling | Multicenter | Prospetively | Nigeria | Aug/2016-Sep/2016 | Not applicable | Rural | Community-based | Not applicable | Other animal species | Sheep | Domesticated animal | Artiodactyla | Indirect ELISA | Antibodies | Serum |
| 4 | Adamu | 2021 | Cross-sectional | Probabilistic | Simple random sampling | Multicenter | Prospetively | Nigeria | Nov/2016-Apr/2017 | Unclear/ Not reported | Unclear/ Not reported | Community-based | Not applicable | Other animal species | Camel | Domesticated animal | Artiodactyla | Indirect ELISA | IgG | Serum |
| 5 | Adesiyun | 2020 | Cross-sectional | Probabilistic | Simple random sampling | Multicenter | Retrospectively | South Africa | 2013 | Not applicable | Rural | Community-based | Not applicable | Other animal species | Cattle | Domesticated animal | Artiodactyla | Indirect ELISA | IgG | Serum |
| 6 | Ahmed | 2018 | Cross-sectional | Non probabilistic | Consecutive sampling | Multicenter | Prospetively | Tanzania | Aug/2014–Oct/2014 | All ages | Rural | Hospital-based | Unclear/ Not reported | Humans |  |  |  | Indirect ELISA | IgG, IgM | Serum |
| 7 | Ahmed | 2000 | Community outbreak | Probabilistic | Simple random sampling | Multicenter | Retrospectively | Sudan | May/2019–Jul/2019 | Unclear/ Not reported | Urban/rural | Community-based | Not applicable | Humans |  |  |  | Classical RT-PCR | Viral RNA | Serum |
| 8 | Alhaji | 2020 | Cross-sectional | Probabilistic | Simple random sampling | Multicenter | Prospetively | Nigeria | Oct/2017-Sep/2018 | Not applicable | Rural | Community-based | Not applicable | Other animal species | Cattle | Domesticated animal | Artiodactyla | Indirect ELISA | IgM | Serum |
| 9 | Andayi | 2014 | Cross-sectional | Probabilistic | Simple random sampling | Multicenter | Prospetively | Djibouti | Nov/2010-Feb/2011 | Unclear/ Not reported | Unclear/ Not reported | Community-based | Not applicable | Humans |  |  |  | Indirect ELISA | IgG | Serum |
| 10 | Andriamandimby | 2010 | Cross-sectional | Non probabilistic | Consecutive sampling | Multicenter | Retrospectively | Madagascar | 2008 | Unclear/ Not reported | Unclear/ Not reported | Hospital-based | Hospitalized | Humans |  |  |  | Indirect ELISA | IgG, IgM | Serum |
| 11 | Andriamandimby | 2018 | Cross-sectional | Non probabilistic | Trapping | Multicenter | Retrospectively | Mali | 2005 -2014 | Not applicable | Unclear/ Not reported | Community-based | Not applicable | Other animal species | Cattle | Domesticated animal | Artiodactyla | Indirect ELISA | IgG | Serum |
| 12 | Anyangu | 2010 | Cross-sectional | Non probabilistic | Consecutive sampling | Multicenter | Prospetively | Kenya | Jan/2007-Mar/2007 | Unclear/ Not reported | Unclear/ Not reported | Community-based | Not applicable | Humans |  |  |  | Indirect ELISA, Classical RT-PCR | IgM, Viral RNA | Serum |
| 13 | Aradaib | 2013 | Cross-sectional | Non probabilistic | Consecutive sampling | Multicenter | Prospetively | Kenya | 2007-2010 | All ages | Urban/rural | Hospital-based | Hospitalized | Humans |  |  |  | Indirect ELISA, Classical RT-PCR | IgM, viral RNA | Serum |
| 14 | Archer | 2011 | Community outbreak | Non probabilistic | Consecutive sampling | Multicenter | Prospetively | South Africa | 2008–2011 | Unclear/ Not reported | Unclear/ Not reported | Hospital-based | Hospitalized | Humans |  |  |  | Indirect ELISA | Antibodies | Serum |
| 15 | Archer | 2013 | Cross-sectional | Non probabilistic | Consecutive sampling | Multicenter | Prospetively | South Africa | 2008–2011 | Unclear/ Not reported | Unclear/ Not reported | Community-based | Not applicable | Humans |  |  |  | Culture, Hemagglutination inhibition test, Indirect ELISA, Real Time RT-PCR, LAMP | Antibodies, Live virus, Viral RNA | Serum |
| 16 | Atuman | 2022 | Cross-sectional | Probabilistic | Simple random sampling | Multicenter | Prospetively | Nigeria | 2013-2015 | Unclear/ Not reported | Rural | Community-based | Not applicable | Other animal species | Cattle, Waterbuck, Eland, Wildebeest, Zebra, Elephant | Domesticated animal and wild animal | Artiodactyla, Perissodactyla, Proboscidea | Indirect ELISA | IgG | Serum |
| 17 | Ayari-Fakhfakh | 2011 | Cross-sectional | Non probabilistic | Consecutive sampling | Multicenter | Retrospectively | Tunisia | Sep/2006-Jan/2007 | Not applicable | Urban/rural | Community-based | Not applicable | Other animal species | Goat, sheep | Domesticated animal | Artiodactyla | Indirect ELISA | Antibodies | Serum |
| 18 | Ba | 2012 | Cross-sectional | Non probabilistic | Capture | Monocenter | Prospetively | Senegal | Jul/2002-Nov/2002; Jul/2003-Nov/2003; | Not applicable | Rural | Community-based | Not applicable | Mosquitoes |  |  |  | Culture | Live virus | Mosquitoes |
| 19 | Baudin | 2016 | Cross-sectional | Non probabilistic | Consecutive sampling | Monocenter | Prospetively | Sudan | Jun/2011-Nov/ 2012. | Adults | Urban | Hospital-based | Hospitalized | Humans |  |  |  | Indirect ELISA | IgM | Serum |
| 20 | Beechler | 2015 | Cross-sectional | Probabilistic | Trapping | Monocenter | Prospetively | South Africa | 2008-2012 | Not applicable | Rural | Community-based | Not applicable | Other animal species | Buffalo | Wild animal | Artiodactyla | Neutralization test | Antibodies | Serum |
| 21 | Bett | 2019 | Cross-sectional | Probabilistic | Simple random sampling | Multicenter | Prospetively | Kenya | Sep/2013- Mar/ 2014 | Adults | Rural | Community-based | Not applicable | Humans, other animal species | Livestock (goat, sheep, cattle) | Domesticated animal | Artiodactyla | Indirect ELISA | IgG | Serum |
| 22 | Bird | 2008 | Community outbreak | Probabilistic | Simple random sampling | Multicenter | Prospetively | Kenya | Nov/2006- May/2007 | Not applicable | Rural | Community-based | Not applicable | Other animal species | Buffalo, Giraffe, Warthog, Camel, Sheep, Goat, Cattle, Buffalo, | Wild animal, Domesticated animal | Artiodactyla | Indirect ELISA, Direct ELISA, Real-time RT-PCR | Viral antigen, IgM, Viral RNA, IgG | Serum |
| 23 | Bisimwa | 2015 | Cross-sectional | Non probabilistic | Capture | Multicenter | Prospetively | Tanzania | Apr/2015-May/2015 | Not applicable | Rural | Community-based | Not applicable | Mosquitoes |  |  |  | Classical RT-PCR | Viral RNA | Mosquitoes |
| 24 | Blomstrom | 2016 | Cross-sectional | Non probabilistic | Consecutive sampling | Multicenter | Prospetively | Mozambique | Sep/2013-Oct/2013 | Not applicable | Rural | Community-based | Not applicable | Other animal species | Goat, sheep | Domesticated animal | Artiodactyla | Indirect ELISA | Antibodies | Serum |
| 25 | Bob | 2017 | Cross-sectional | Non probabilistic | Consecutive sampling | Multicenter | Prospetively | Mauritania | Sep/2015-Nov/2015 | All ages | Urban | Hospital-based | Hospitalized | Humans |  |  |  | Indirect ELISA, Classical RT-PCR | IgM, Viral RNA | Serum |
| 26 | Bob | 2022 | Cross-sectional | Non probabilistic | Consecutive sampling | Multicenter | Prospetively | Senegal | Jan/2020-Dec/2020 | Unclear/ Not reported | Unclear/ Not reported | Hospital-based | Unclear/ Not reported | Humans |  |  |  | Indirect ELISA, Classical RT-PCR | IgM, Viral RNA | Serum |
| 27 | Bonney | 2013 | Cross-sectional | Non probabilistic | Consecutive sampling | Multicenter | Retrospectively | Ghana | 2008-2011 | Unclear/ Not reported | Urban/rural | Community-based | Not applicable | Humans |  |  |  | Classical RT-PCR | Viral RNA | Serum |
| 28 | Bosworth | 2016 | Cross-sectional | Non probabilistic | Consecutive sampling | Multicenter | Prospetively | Tunisia | 2014 | Unclear/ Not reported | Urban/rural | Hospital-based | Hospitalized | Humans |  |  |  | Indirect immunofluorescence assay | IgG, IgM | Serum |
| 29 | Boushab | 2015 | Cross-sectional | Non probabilistic | Consecutive sampling | Multicenter | Prospetively | Mauritania | Oct/2012-Nov/2012 | Unclear/ Not reported | Rural | Community-based | Not applicable | Humans |  |  |  | Classical RT-PCR | Viral RNA | Serum |
| 30 | Boushab | 2016 | Cross-sectional | Non probabilistic | Consecutive sampling | Multicenter | Prospetively | Mauritania | Sep/2015-Nov/2015 | All ages | Unclear/ Not reported | Hospital-based | Hospitalized | Humans |  |  |  | Indirect ELISA | IgM | Serum |
| 31 | Boussini | 2014 | Cross-sectional | Non probabilistic | Consecutive sampling | Multicenter | Retrospectively | Burkina Faso | Unclear/ Not reported | Not applicable | Urban/rural | Community-based | Not applicable | Other animal species | Cattle, Sheep, Goat | Domesticated animal | Artiodactyla | Indirect ELISA | IgG | Serum |
| 32 | Boussini | 2013 | Cross-sectional | Non probabilistic | Consecutive sampling | Multicenter | Prospetively | Burkina Faso | Unclear/ Not reported | Not applicable | Rural | Community-based | Not applicable | Other animal species | Camel | Domesticated animal | Artiodactyla | Neutralization test | Antibodies | Serum |
| 33 | Bronsvoort | 2022 | Cross-sectional | Probabilistic | Multistage sampling | Multicenter | Prospetively | Cameroon | Jan/2013-May/2013; Sep/2013-Nov/2013 | Unclear/ Not reported | Unclear/ Not reported | Community-based | Not applicable | Other animal species | Cattle | Domesticated animal | Artiodactyla | Indirect ELISA | IgG | Serum |
| 34 | Budasha | 2018 | Cross-sectional | Probabilistic | Stratified sampling | Multicenter | Prospetively | Uganda | 2016 | Not applicable | Rural | Community-based | Not applicable | Other animal species | Cattle, Sheep, Goat | Domesticated animal | Artiodactyla | Indirect ELISA | IgG | Serum |
| 35 | Budodo | 2020 | Cross-sectional | Non probabilistic | Convenience sampling | Multicenter | Prospetively | Tanzania | Jun/2019-Aug/2019 | Adults | Rural | Community-based | Not applicable | Humans |  |  |  | Indirect ELISA | Antibodies | Serum |
| 36 | Bukbuk | 2014 | Cross-sectional | Probabilistic | Simple random sampling | Multicenter | Prospetively | Nigeria | Sep/2011-Feb/2012 | Unclear/ Not reported | Urban | Hospital-based | Unclear/ Not reported | Humans |  |  |  | Indirect ELISA, Neutralisation test | IgG | Serum |
| 37 | Capobianco Dondona | 2016 | Cross-sectional | Probabilistic | Simple random sampling | Monocenter | Prospetively | Namibia | May/2011–Dec/2011 | Not applicable | Rural | Community-based | Not applicable | Other animal species | Springbok, Wildebeest, Black-faced impala | Wild animal | Artiodactyla | Indirect ELISA, Real Time RT-PCR | Antibodies, IgM, Viral RNA | Serum |
| 38 | Centers for Disease Control and Prevention (CDC) | 2007 | Community outbreak | Non probabilistic | Consecutive sampling | Multicenter | Prospetively | Kenya | Nov/2006--Jan/2007 | Unclear/ Not reported | Unclear/ Not reported | Community-based | Not applicable | Humans |  |  |  | Indirect ELISA, Classical RT-PCR | IgM, Viral RNA | Serum |
| 39 | Chambaro | 2022 | Cross-sectional | Probabilistic | Simple random sampling | Multicenter | Prospetively | Zambia | Aug/2018-May/2019 | Unclear/ Not reported | Urban/rural | Community-based | Not applicable | Other animal species | Buffalo, Impala, Warthog, Hartebeest, Sheep, Goat, Cattle | Wild animal, Domesticated animal | Artiodactyla | Indirect ELISA | IgG | Serum |
| 40 | Chengula | 2014 | Cross-sectional | Non probabilistic | Consecutive sampling | Multicenter | Retrospectively | Tanzania | Mar/2007-Nov/2007 | Not applicable | Unclear/ Not reported | Community-based | Not applicable | Other animal species | Cattle, Sheep, Goat | Domesticated animal | Artiodactyla | Indirect ELISA, Classical RT-PCR | IgG, Viral RNA | Serum |
| 41 | Chevalier | 2005 | Cross-sectional | Non probabilistic | Consecutive sampling | Multicenter | Prospetively | Senegal | 2003 | Not applicable | Rural | Community-based | Not applicable | Other animal species | Small ruminants (Goat and Sheep) | Domesticated animal | Artiodactyla | Neutralization test | Antibodies | Serum |
| 42 | Chevalier | 2011 | Cross-sectional | Probabilistic | Simple random sampling | Multicenter | Prospetively | Madagascar | May/2009-Jun/2009 | Not applicable | Rural | Community-based | Not applicable | Other animal species | Cattle | Domesticated animal | Artiodactyla | Indirect ELISA | IgG, IgM | Serum |
| 43 | Cichon | 2021 | Cross-sectional | Non probabilistic | Consecutive sampling | Multicenter | Prospetively | Mauritania | Feb/2015-Nov/2015 | Children | Unclear/ Not reported | Community-based | Not applicable | Other animal species | Small ruminants (Goat and Sheep) | Domesticated animal | Artiodactyla | Indirect ELISA, Real Time RT-PCR | IgG, IgM, Viral RNA | Serum |
| 44 | Clements | 2019 | Cross-sectional | Non probabilistic | Consecutive sampling | Multicenter | Prospetively | Uganda | 2006-2007 | Unclear/ Not reported | Unclear/ Not reported | Community-based | Not applicable | Humans |  |  |  | Indirect ELISA | IgG | Serum |
| 45 | Cook | 2017 | Cross-sectional | Probabilistic | Cluster sampling | Multicenter | Prospetively | Kenya | Jul/2010-Jul/2012 | All ages | Rural | Community-based | Not applicable | Humans |  |  |  | Indirect ELISA | IgG | Serum |
| 46 | Cosseddu | 2021 | Cross-sectional | Probabilistic | Simple random sampling | Monocenter | Prospetively | Mauritania | mars-13 | Unclear/ Not reported | Urban | Community-based | Not applicable | Other animal species | Camel, Cattle | Domesticated animal | Artiodactyla | Indirect ELISA | IgG | Serum |
| 47 | Di Nardo | 2014 | Cross-sectional | Probabilistic | Cluster sampling | Multicenter | Prospetively | Algeria | Mar/2008-Apr/2008 | Not applicable | Unclear/ Not reported | Community-based | Not applicable | Other animal species | Camel, Goat, Sheep | Domesticated animal | Artiodactyla | Indirect ELISA | IgG | Serum |
| 48 | Diallo | 2000 | Cross-sectional | Non probabilistic | Capture | Multicenter | Prospetively | Senegal | Unclear/ Not reported | Not applicable | Unclear/ Not reported | Community-based | Not applicable | Mosquitoes |  |  |  | Culture | Live virus | Mosquitoes |
| 49 | Diallo | 2005 | Cross-sectional | Non probabilistic | Capture | Multicenter | Prospetively | Mauritanie, Senegal | Nov/1998; Dec/1998; August/1999; Oct/1999-Nov/1999 | Not applicable | Unclear/ Not reported | Community-based | Not applicable | Mosquitoes |  |  |  | Culture | Live virus | Mosquitoes |
| 50 | Dione | 2022 | Cross-sectional | Probabilistic | Simple random sampling | Multicenter | Prospetively | Mali | 2016 | Unclear/ Not reported | Unclear/ Not reported | Community-based | Not applicable | Other animal species | Cattle, Sheep, Goat | Domesticated animal | Artiodactyla | Indirect ELISA | IgG | Serum |
| 51 | Durand | 2003 | Cross-sectional | Non probabilistic | Consecutive sampling | Multicenter | Prospetively | Chad | Aug/2001-Sep/2001 | Unclear/ Not reported | Unclear/ Not reported | Community-based | Not applicable | Humans |  |  |  | Indirect ELISA, Culture | IgG, IgM, Live virus | Serum |
| 52 | Durand | 2020 | Cross-sectional | Probabilistic | Simple random sampling | Multicenter | Prospetively | Senegal | May/2016 | Not applicable | Unclear/ Not reported | Community-based | Not applicable | Other animal species | Cattle, Sheep, Goat | Domesticated animal | Artiodactyla | Indirect ELISA | IgG | Serum |
| 53 | Dutuze | 2020 | Cross-sectional | Non probabilistic | Consecutive sampling | Multicenter | Prospetively | Rwanda | May/2018-Jul/2018. | Not applicable | Unclear/ Not reported | Community-based | Not applicable | Other animal species | Cattle, Goat | Domesticated animal | Artiodactyla | Classical RT-PCR | Viral RNA | Serum |
| 54 | Ebogo-Belobo | 2022 | Cross-sectional | Non probabilistic | Consecutive sampling | Monocenter | Prospetively | Cameroon | mars-20 | Unclear/ Not reported | Urban | Community-based | Not applicable | Other animal species | Goat, sheep | Domesticated animal | Artiodactyla | Indirect ELISA | IgG | Serum |
| 55 | Eckstein | 2022 | Cross-sectional | Non probabilistic | Consecutive sampling | Multicenter | Prospetively | Tunisia | janv-20 | Unclear/ Not reported | Rural | Community-based | Not applicable | Other animal species | Camel | Domesticated animal | Artiodactyla | Indirect ELISA | IgG | Serum |
| 56 | El Bahgy | 2018 | Cross-sectional | Non probabilistic | Consecutive sampling | Multicenter | Prospetively | Egypt | Unclear/ Not reported | Not applicable | Unclear/ Not reported | Community-based | Not applicable | Other animal species | Camel | Domesticated animal | Artiodactyla | Indirect ELISA | IgG | Serum |
| 57 | El Mamy | 2011 | Cross-sectional | Non probabilistic | Consecutive sampling | Multicenter | Prospetively | Mauritania | Sep/2010-Oct/2010 | Not applicable | Rural | Community-based | Not applicable | Other animal species | Camel, Small ruminants (Goat and Sheep) | Domesticated animal | Artiodactyla | Indirect ELISA | IgM, Antibodies | Serum |
| 58 | El Mamy | 2010 | Cross-sectional | Non probabilistic | Consecutive sampling | Multicenter | Prospetively | Mauritania | oct-10 | Not applicable | Rural | Community-based | Not applicable | Other animal species | Camel | Domesticated animal | Artiodactyla | Classical RT-PCR | Viral RNA | Serum |
| 59 | El-Harrak | 2011 | Cross-sectional | Non probabilistic | Consecutive sampling | Multicenter | Prospetively | Morocco | 2009 | Not applicable | Rural | Community-based | Not applicable | Other animal species | Camel | Domesticated animal | Artiodactyla | Indirect ELISA | Antibodies | Serum |
| 60 | Endale | 2021 | Cross-sectional | Non probabilistic | Consecutive sampling | Multicenter | Prospetively | Ethiopia | May/2019-Jun/2019 | Not applicable | Rural | Community-based | Not applicable | Other animal species | Cattle | Domesticated animal | Artiodactyla | Indirect ELISA | IgG | Serum |
| 61 | Enem | 2020 | Cross-sectional | Non probabilistic | Consecutive sampling | Multicenter | Prospetively | Guinea | 2011-2012 | Unclear/ Not reported | Unclear/ Not reported | Community-based | Not applicable | Humans |  |  |  | Multiplex microsphere immunoassay | IgG | Serum |
| 62 | EVANS | 2008 | Cross-sectional | Non probabilistic | Trapping | Multicenter | Prospetively | Kenya | 1999-2005; Jan/2007-Fev/2007 | Not applicable | Rural | Community-based | Not applicable | Other animal species | Buffalo, Elephant, Warthog, Black rhino, Zebra, Giraffe, Kongoni, Lion, Kudu, Waterbuck | Wild animal | Artiodactyla, Perissodactyla, Carnivora | Indirect ELISA | IgG | Serum |
| 63 | Fafetine | 2012 | Cross-sectional | Non probabilistic | Consecutive sampling | Monocenter | Prospetively | Mozambique | 2010-2011 | Not applicable | Unclear/ Not reported | Community-based | Not applicable | Other animal species | Goat, sheep | Domesticated animal | Artiodactyla | Indirect ELISA | IgG | Serum |
| 64 | Fafetine | 2013 | Cross-sectional | Probabilistic | Systematic sampling | Multicenter | Prospetively | Mozambique | 2007; Sep/2010-Apr/2011 | Not applicable | Rural | Community-based | Not applicable | Other animal species | Goat, sheep | Domesticated animal | Artiodactyla | Indirect ELISA | IgG | Serum |
| 65 | Fafetine | 2014 | Cross-sectional | Non probabilistic | Consecutive sampling | Multicenter | Prospetively | Mozambique | March 2014 | Not applicable | Rural | Community-based | Not applicable | Other animal species | Small ruminants (Goat and Sheep) | Domesticated animal | Artiodactyla | Indirect ELISA | IgG, IgM | Serum |
| 66 | Fagbo | 2014 | Cross-sectional | Non probabilistic | Consecutive sampling | Multicenter | Prospetively | South Africa | 2003-2004 | Not applicable | Rural | Community-based | Not applicable | Other animal species | Buffalo | Wild animal | Artiodactyla | Indirect ELISA | IgG | Serum |
| 67 | Faye | 2010 | Community outbreak | Non probabilistic | Consecutive sampling | Multicenter | Prospetively | Mauritania | Oct/2010-Dec/2010 | Adults | Unclear/ Not reported | Community-based | Not applicable | Humans, other animal species, Mosquitoes | Goat, sheep | Domesticated animal | Artiodactyla | Real Time RT-PCR, Indirect ELISA, Culture | IgM, Viral RNA, Live virus | Serum, Mosquitoes |
| 68 | Faye | 2007 | Cross-sectional | Non probabilistic | Consecutive sampling | Multicenter | Prospetively | Mauritania | Sep/2003-Dec/2003 | All ages | Unclear/ Not reported | Community-based | Not applicable | Humans, other animal species, Mosquitoes | Goat | Domesticated animal | Artiodactyla | Classical RT-PCR, Indirect ELISA, Culture | IgG, IgM, Viral RNA, Live virus | Serum, Mosquitoes |
| 69 | Fischer-Tenhagen | 2000 | Cross-sectional | Non probabilistic | Trapping | Multicenter | Prospetively | Kenya, Namibia, South Africa | 1987-1997 | Not applicable | Unclear/ Not reported | Community-based | Not applicable | Other animal species | Rhinoceros | Wild animal | Perissodactyla | Indirect ELISA | Antibodies | Serum |
| 70 | Fokam | 2010 | Cross-sectional | Non probabilistic | Consecutive sampling | Multicenter | Prospetively | Cameroon | Unclear/ Not reported | Unclear/ Not reported | Urban | Hospital-based | Ambulatory | Humans |  |  |  | Hemagglutination inhibition test | Antibodies | Serum |
| 71 | Georges | 2018 | Cross-sectional | Probabilistic | Simple random sampling | Multicenter | Prospetively | Democratic Republic of the Congo | Nov/2013-Dec/2013 | Not applicable | Unclear/ Not reported | Community-based | Not applicable | Other animal species | Cattle | Domesticated animal | Artiodactyla | Indirect ELISA | IgM | Serum |
| 72 | Gora | 2000 | Cross-sectional | Non probabilistic | Trapping | Multicenter | Prospetively | Senegal | Jun/1996-Apr/1998 | Not applicable | Rural | Community-based | Not applicable | Other animal species | Rodents | Wild animal | Rodentia | Neutralization test | Antibodies | Serum |
| 73 | Gray | 2015 | Cross-sectional | Non probabilistic | Consecutive sampling | Multicenter | Prospetively | Madagascar, Kenya | Jul/2010-Jun/2012 | Adults | Unclear/ Not reported | Community-based | Not applicable | Humans |  |  |  | Indirect ELISA | IgG | Serum |
| 74 | Grolla | 2012 | Cross-sectional | Non probabilistic | Consecutive sampling | Multicenter | Prospetively | Kenya | Jan/2007-Jan/2007 | Unclear/ Not reported | Unclear/ Not reported | Hospital/community based | Ambulatory | Humans |  |  |  | Real Time RT-PCR | Viral RNA | Serum |
| 75 | Grossi-soyster | 2017 | Cross-sectional | Non probabilistic | Consecutive sampling | Multicenter | Prospetively | Kenya | 2009-2011 | All ages | Rural | Community-based | Not applicable | Humans |  |  |  | Indirect ELISA | IgG | Serum |
| 76 | Gudo | 2016 | Cross-sectional | Non probabilistic | Consecutive sampling | Monocenter | Prospetively | Mozambique | 2012–2013 | All ages | Urban/rural | Hospital-based | Ambulatory | Humans |  |  |  | Indirect immunofluorescence assay | IgG | Serum |
| 77 | Gudo | 2016 | Cross-sectional | Non probabilistic | Consecutive sampling | Monocenter | Prospetively | Mozambique | Jan/2013-Sep/2013 | Unclear/ Not reported | Urban/rural | Hospital-based | Ambulatory | Humans |  |  |  | Indirect ELISA, Real Time RT-PCR | IgG, Viral RNA | Serum |
| 78 | Guillebaud | 2018 | Cross-sectional | Probabilistic | Simple random sampling | Multicenter | Prospetively | Madagascar | Apr/2014-Sep/2015. | All ages | Urban/rural | Community-based | Not applicable | Humans |  |  |  | Classical RT-PCR | Viral RNA | Serum |
| 79 | Halawi | 2019 | Cross-sectional | Probabilistic | Simple random sampling | Multicenter | Prospetively | Democratic Republic of the Congo | 2017 | Not applicable | Rural | Community-based | Not applicable | Other animal species | Cattle | Domesticated animal | Artiodactyla | Immunofluorescent assay | Antibodies | Serum |
| 80 | Hanafi | 2011 | Cross-sectional | Non probabilistic | Consecutive sampling | Multicenter | Retrospectively | Egypt | Aug/2003-Sep/2003 | Not applicable | Urban/rural | Community-based | Not applicable | Other animal species, Mosquitoes | Cattle, sheep | Domesticated animal | Artiodactyla | Immunoassay, Culture | IgM, Live virus | Serum, Mosquitoes |
| 81 | Hassan | 2020 | Cross-sectional | Non probabilistic | Consecutive sampling | Multicenter | Prospetively | Kenya | May/2018-Jun/2018 | Unclear/ Not reported | Rural | Community-based | Not applicable | Humans, other animal species | Camel, Goat, Sheep | Domesticated animal | Artiodactyla | Indirect ELISA, Classical RT-PCR | IgM, Viral RNA | Serum |
| 82 | Hassanain | 2010 | Cross-sectional | Non probabilistic | Consecutive sampling | Monocenter | Prospetively | Sudan | Sep/2007-Nov/2007 | Unclear/ Not reported | Rural | Hospital-based | Ambulatory | Humans |  |  |  | Indirect ELISA | IgG | Serum |
| 83 | Hassine | 2017 | Cross-sectional | Non probabilistic | Consecutive sampling | Multicenter | Prospetively | Tunisia | 2016 | Not applicable | Unclear/ Not reported | Community-based | Not applicable | Other animal species | Dromedaries | Domesticated animal | Artiodactyla | Indirect ELISA | Antibodies | Serum |
| 84 | Heinrich | 2012 | Cross-sectional | Probabilistic | Stratified sampling | Multicenter | Prospetively | Tanzania | Jun/2007-Jun/2008 | Unclear/ Not reported | Urban/rural | Community-based | Not applicable | Humans |  |  |  | Indirect immunofluorescence assay | IgG | Serum |
| 85 | Horton | 2014 | Cross-sectional | Non probabilistic | Consecutive sampling | Monocenter | Prospetively | Egypt | juil-09 | Not applicable | Urban | Community-based | Not applicable | Other animal species | Buffalo, Cattle, Sheep, Goat | Wild animal, Domesticated animal | Artiodactyla | Indirect ELISA | IgG | Serum |
| 86 | Ibrahim | 2021 | Cross-sectional | Probabilistic | Systematic sampling | Multicenter | Prospetively | Ethiopia | May/2016-Aug/2016. | Adults | Rural | Community-based | Not applicable | Humans, other animal species | Cattle, Camel, Goat, Sheep | Domesticated animal | Artiodactyla | Indirect ELISA | Antibodies | Serum |
| 87 | Jäckel | 2013 | Cross-sectional | Non probabilistic | Consecutive sampling | Multicenter | Prospetively | Mauritania | Dec/2010-Feb/2011 | Not applicable | Unclear/ Not reported | Community-based | Not applicable | Other animal species | Camel, Small ruminants (Goat and Sheep) | Domesticated animal | Artiodactyla | Indirect ELISA | IgG, IgM, Antibodies | Serum |
| 88 | Jeanmaire | 2011 | Cross-sectional | Probabilistic | Stratified sampling | Multicenter | Prospetively | Madagascar | Aug/2008 | Not applicable | Unclear/ Not reported | Community-based | Not applicable | Other animal species | Cattle, Small ruminants (Goat and Sheep) | Domesticated animal | Artiodactyla | Indirect ELISA | IgG, IgM | Serum |
| 89 | Jori | 2015 | Cross-sectional | Non probabilistic | Trapping | Multicenter | Prospetively | Botswana | 2010-2011 | Not applicable | Rural | Community-based | Not applicable | Other animal species | Buffalo, Cattle | Wild animal, Domesticated animal | Artiodactyla | Neutralization test | Antibodies | Serum |
| 90 | Kading | 2018 | Cross-sectional | Non probabilistic | Trapping | Multicenter | Prospetively | Egypt, Uganda | 2009-2013 | Not applicable | Rural | Community-based | Not applicable | Other animal species | Bat | Wild animal | Chiroptera | Neutralization test | Antibodies | Serum |
| 91 | Kanoute | 2017 | Cross-sectional | Probabilistic | Simple random sampling | Multicenter | Prospetively | Ivory Coast | May/2012 - Jul/2012 and May/2013 - Dec/ 2013 and Mar/2014 - Jun/2014 | Not applicable | Urban/rural | Community-based | Not applicable | Other animal species | Cattle, Sheep, Goat | Domesticated animal | Artiodactyla | Indirect ELISA | IgG | Serum |
| 92 | Kifaro | 2014 | Cross-sectional | Non probabilistic | Consecutive sampling | Multicenter | Prospetively | Tanzania | May/2011-Jun/2011 | Not applicable | Urban/rural | Community-based | Not applicable | Other animal species | Goat, sheep | Domesticated animal | Artiodactyla | Indirect ELISA | IgG | Serum |
| 93 | LaBeaud | 2008 | Cross-sectional | Probabilistic | Cluster sampling | Multicenter | Prospetively | Kenya | Mar/2006-Apr/2006 | Unclear/ Not reported | Urban/rural | Community-based | Not applicable | Humans |  |  |  | Indirect ELISA | IgG | Serum |
| 94 | LaBeaud | 2011 | Cross-sectional | Non probabilistic | Consecutive sampling | Multicenter | Prospetively | South Africa | 2001–2006 | Not applicable | Rural | Community-based | Not applicable | Other animal species | Buffalo | Wild animal | Artiodactyla | Hemagglutination inhibition test | Antibodies | Serum |
| 95 | LaBeaud | 2011 | Cohort (Baseline data) | Probabilistic | Cluster sampling | Multicenter | Prospetively | Kenya | Aug/2009-Nov/2009 | All ages | Urban/rural | Community-based | Not applicable | Humans |  |  |  | Indirect ELISA | IgG | Serum |
| 96 | LaBeaud | 2011 | Cross-sectional | Non probabilistic | Capture | Multicenter | Prospetively | Kenya | 2006 and (Dec/2006–Jan/2007) | Not applicable | Urban/rural | Community-based | Not applicable | Mosquitoes |  |  |  | Real Time RT-PCR | Viral RNA | Mosquitoes |
| 97 | LaBeaud | 2015 | Cross-sectional | Probabilistic | Systematic sampling | Multicenter | Prospetively | Kenya | Aug/2011-Nov/2011 | Unclear/ Not reported | Rural | Community-based | Not applicable | Humans |  |  |  | Indirect ELISA | IgG | Serum |
| 98 | Lagare | 2019 | Community outbreak | Non probabilistic | Consecutive sampling | Multicenter | Prospetively | Niger | Aug/2016-Dec/2016 | All ages | Urban/rural | Community-based | Unclear/ Not reported | Humans, other animal species | Sheep, Goat, Camel, Cattle, unspecified animal | Wild animal, Domesticated animal | Artiodactyla | Classical RT-PCR, Real Time RT-PCR, Indirect ELISA | Viral RNA, Antibodies | Serum |
| 99 | Lagerqvist | 2013 | Cross-sectional | Non probabilistic | Consecutive sampling | Multicenter | Prospetively | Mozambique | 2010–2011 | Not applicable | Urban/rural | Community-based | Not applicable | Other animal species | Animal unspecified | Wild animal | Unclassified | Neutralization test | Antibodies | Serum |
| 100 | LeBreton | 2006 | Cross-sectional | Non probabilistic | Consecutive sampling | Multicenter | Prospetively | Cameroon | 2003 | Not applicable | Urban/rural | Community-based | Not applicable | Other animal species | Goat | Domesticated animal | Artiodactyla | Indirect ELISA | IgG | Serum |
| 101 | Lubisi | 2020 | Cross-sectional | Non probabilistic | Consecutive sampling | Multicenter | Prospetively | South Africa | 2007 and 2012–2015 | Not applicable | Urban/rural | Community-based | Not applicable | Other animal species | Pig, Warthog | Wild animal, Domesticated animal | Artiodactyla | Neutralization test | Antibodies | Serum |
| 102 | Lutomiah | 2014 | Cross-sectional | Non probabilistic | Capture | Multicenter | Prospetively | Kenya | Dec/2006-Mar/2007 | Not applicable | Urban/rural | Community-based | Not applicable | Mosquitoes |  |  |  | Real Time RT-PCR | Viral RNA | Mosquitoes |
| 103 | Lwande | 2015 | Cross-sectional | Non probabilistic | Consecutive sampling | Multicenter | Prospetively | Kenya | 2008–2015 | Not applicable | Urban/rural | Community-based | Not applicable | Other animal species | Buffalo, Black rhino, Buffalo, Elephant, Vervet Monkey, Warthog, Wildebeest, Cattle, Baboon | Wild animal, Domesticated animal | Artiodactyla, Perissodactyla, Primate | Indirect ELISA | IgG | Serum |
| 104 | Lysholm | 2022 | Cross-sectional | Probabilistic | Simple random sampling | Multicenter | Prospetively | Zambia; Tanzania | Sep/2018-Oct/2018 | Unclear/ Not reported | Rural | Community-based | Not applicable | Other animal species | Goat, sheep | Domesticated animal | Artiodactyla | Indirect ELISA | IgG | Serum |
| 105 | Maganga | 2017 | Cross-sectional | Non probabilistic | Consecutive sampling | Multicenter | Prospetively | Gabon | Jun/2014-Sep/2014 | Not applicable | Urban/rural | Community-based | Not applicable | Other animal species | Goat, sheep | Domesticated animal | Artiodactyla | Indirect ELISA, Real Time RT-PCR | IgG, Viral RNA | Serum |
| 106 | Magona | 2013 | Cross-sectional | Probabilistic | Systematic sampling | Multicenter | Prospetively | Uganda | 2009 | Not applicable | Rural | Community-based | Not applicable | Other animal species | Goat | Domesticated animal | Artiodactyla | Indirect ELISA | IgG | Serum |
| 107 | Mahmoud | 2018 | Cross-sectional | Non probabilistic | Consecutive sampling | Multicenter | Prospetively | Libya | 2015-2016 | Not applicable | Urban | Community-based | Not applicable | Other animal species | Cattle, Small ruminants (Goat and Sheep) | Domesticated animal | Artiodactyla | Indirect ELISA | Antibodies | Serum |
| 108 | Mahmoud | 2021 | Cross-sectional | Non probabilistic | Consecutive sampling | Multicenter | Prospetively | Egypt | May/2017-Jun/2019 | Unclear/ Not reported | Rural | Community-based | Not applicable | Other animal species | Cattle, Sheep, Camel, Donkey, Goat | Domesticated animal | Artiodactyla, Perissodactyla | Indirect ELISA | IgG | Serum |
| 109 | Makiala-Mandanda | 2018 | Cross-sectional | Non probabilistic | Consecutive sampling | Monocenter | Prospetively | Democratic Republic of the Congo | 2003-2012 | All ages | Unclear/ Not reported | Unclear/ Not reported | Unclear/ Not reported | Humans |  |  |  | Real Time RT-PCR | Viral RNA | Serum |
| 110 | Mapaco | 2012 | Cross-sectional | Non probabilistic | Consecutive sampling | Multicenter | Prospetively | South Africa | Mar/2008-May/2008 | Not applicable | Rural | Community-based | Not applicable | Other animal species | Cattle, Sheep | Domesticated animal | Artiodactyla | Indirect ELISA | IgG, IgM | Serum |
| 111 | Marietou | 2019 | Cross-sectional | Non probabilistic | Consecutive sampling | Multicenter | Prospetively | Niger | 2017 | Not applicable | Urban/rural | Community-based | Not applicable | Other animal species | Cattle, Sheep, Goat | Domesticated animal | Artiodactyla | Indirect ELISA | Antibodies | Serum |
| 112 | Marrama | 2005 | Cross-sectional | Probabilistic | Cluster sampling | Multicenter | Prospetively | Senegal | 1999 | All ages | Rural | Community-based | Not applicable | Humans |  |  |  | Indirect ELISA | IgG | Serum |
| 113 | Matiko | 2018 | Cross-sectional | Probabilistic | Multistage sampling | Multicenter | Prospetively | Tanzania | Jun/2014-Oct/2015 | Not applicable | Urban/rural | Community-based | Not applicable | Other animal species | Cattle | Domesticated animal | Artiodactyla | Indirect ELISA | Antibodies, IgM | Serum |
| 114 | Mbotha | 2018 | Cross-sectional | Probabilistic | Simple random sampling | Multicenter | Prospetively | Kenya | Sep/2014–Jun/2015 | Not applicable | Urban/rural | Community-based | Not applicable | Other animal species | Goat, sheep | Domesticated animal | Artiodactyla | Indirect ELISA | IgG | Serum |
| 115 | Mease | 2011 | Cross-sectional | Probabilistic | Multistage sampling | Multicenter | Prospetively | Kenya | 2004 | Adults | Rural | Community-based | Not applicable | Humans |  |  |  | Indirect ELISA | IgG | Serum |
| 116 | Mhina | 2015 | Cross-sectional | Non probabilistic | Capture | Multicenter | Prospetively | Tanzania | May/2013 | Not applicable | Unclear/ Not reported | Community-based | Not applicable | Mosquitoes |  |  |  | Real Time RT-PCR | Viral RNA | Mosquitoes |
| 117 | Miller | 2011 | Cross-sectional | Probabilistic | Trapping | Monocenter | Prospetively | South Africa | Feb/2007-Aug/2007 | Not applicable | Rural | Community-based | Not applicable | Other animal species | Rhinoceros | Wild animal | Perissodactyla | Indirect ELISA | Antibodies | Serum |
| 118 | Mohamed | 2010 | Community outbreak | Non probabilistic | Consecutive sampling | Multicenter | Prospetively | Tanzania | Feb/2007-Jun/2007 | Adults | Urban/rural | Hospital-based | Hospitalized/ambulatory | Humans |  |  |  | Indirect ELISA, Real Time RT-PCR | IgM, Viral RNA | Serum |
| 119 | Mohamed | 2019 | Cross-sectional | Non probabilistic | Consecutive sampling | Monocenter | Prospetively | Sudan | Jun/2015–Nov/2015 | Unclear/ Not reported | Urban | Hospital-based | Unclear/ Not reported | Humans |  |  |  | Real Time RT-PCR | Viral RNA | Serum |
| 120 | Moiane | 2017 | Cross-sectional | Non probabilistic | Consecutive sampling | Multicenter | Prospetively | Mozambique | Apr/2013-Jun/2014 | Not applicable | Urban/rural | Community-based | Not applicable | Other animal species | Cattle, Buffalo, Sheep, Goat | Wild animal, Domesticated animal | Artiodactyla | Indirect ELISA | Antibodies | Serum |
| 121 | Monaco | 2013 | Community outbreak | Non probabilistic | Consecutive sampling | Multicenter | Prospetively | Namibia | May/2010-July/2010 | Not applicable | Rural | Community-based | Not applicable | Other animal species | Goat, sheep | Domesticated animal | Artiodactyla | Classical RT-PCR, Culture | Viral RNA, Live virus | Serum |
| 122 | Mordi | 2020 | Cross-sectional | Non probabilistic | Consecutive sampling | Multicenter | Prospetively | Ethiopia | Dec/2018-Jan/2019 | Not applicable | Unclear/ Not reported | Community-based | Not applicable | Other animal species | Cattle | Domesticated animal | Artiodactyla | Indirect ELISA | IgG | Serum |
| 123 | Mroz | 2017 | Cross-sectional | Probabilistic | Simple random sampling | Multicenter | Prospetively | Egypt | 2014-2015 | Not applicable | Unclear/ Not reported | Community-based | Not applicable | Other animal species | Sheep, Goat, Camel, Cattle, Buffalo | Wild animal, Domesticated animal | Artiodactyla | Indirect ELISA | Antibodies | Serum |
| 124 | Mroz | 2017 | Cross-sectional | Probabilistic | Simple random sampling | Multicenter | Prospetively | Egypt | 2013-2015 | Not applicable | Unclear/ Not reported | Community-based | Not applicable | Other animal species | Cattle | Domesticated animal | Artiodactyla | Indirect ELISA | Antibodies | Serum |
| 125 | Msimang | 2019 | Cross-sectional | Non probabilistic | Consecutive sampling | Multicenter | Prospetively | South Africa | Oct/2015-Feb/2016 | Adults | Unclear/ Not reported | Community-based | Not applicable | Humans |  |  |  | Indirect ELISA | IgG | Serum |
| 126 | Muiruri | 2015 | Cross-sectional | Probabilistic | Cluster sampling | Multicenter | Prospetively | Kenya | sept-06 | All ages | Rural | Community-based | Not applicable | Humans |  |  |  | Indirect ELISA | IgG | Serum |
| 127 | Munyua | 2010 | Cross-sectional | Probabilistic | Simple random sampling | Nationally representative (as stated by authors) | Prospetively | Kenya | Dec/2006-Jun/2007 | Unclear/ Not reported | Urban/rural | Community-based | Not applicable | Humans, other animal species |  |  |  | Indirect ELISA, Real Time RT-PCR | IgG, IgM, Viral RNA | Serum |
| 128 | Nabeth | 2001 | Community outbreak | Non probabilistic | Consecutive sampling | Multicenter | Prospetively | Mauritania | Sep/1998-Dec/1998 | Unclear/ Not reported | Unclear/ Not reported | Hospital/community based | Hospitalized/ambulatory | Humans, other animal species, Mosquitoes | Rodents, Sheep, Goat, Camel, Cattle, Donkeys | Wild animal, Domesticated animal | Rodentia, Artiodactyla, Perissodactyla | Indirect ELISA, Culture | IgM, Live virus | Serum |
| 129 | Nakouné | 2016 | Cross-sectional | Non probabilistic | Consecutive sampling | Multicenter | Prospetively | Central African Republic | Nov/2010-Nov/2012 | Adults | Rural | Community-based | Not applicable | Humans, other animal species | Cattle, Sheep, Goat | Domesticated animal | Artiodactyla | Indirect ELISA | IgG, IgM | Serum |
| 130 | Nakounne | 2001 | Cross-sectional | Non probabilistic | Consecutive sampling | Multicenter | Retrospectively | Central African Republic | 1992-1998 | Unclear/ Not reported | Rural | Community-based | Not applicable | Humans |  |  |  | Indirect ELISA | IgG, IgM | Serum |
| 131 | Nanyingi | 2017 | Cross-sectional | Probabilistic | Simple random sampling | Multicenter | Prospetively | Kenya | juil-13 | Not applicable | Rural | Community-based | Not applicable | Other animal species | Cattle, Sheep, Goat | Domesticated animal | Artiodactyla | Indirect ELISA | IgG | Serum |
| 132 | Ndengu | 2020 | Cross-sectional | Probabilistic | Systematic sampling | Multicenter | Prospetively | Zimbabwe | Unclear/ Not reported | Not applicable | Rural | Community-based | Not applicable | Other animal species | Buffalo, Kudu, Impala, Cattle | Wild animal, Domesticated animal | Artiodactyla | Indirect ELISA | Antibodies | Serum |
| 133 | Ndiana | 2019 | Cross-sectional | Probabilistic | Systematic sampling | Multicenter | Prospetively | South Africa | Jun/2016, Feb/2017-Apr/2017 | Not applicable | Unclear/ Not reported | Community-based | Not applicable | Other animal species | Cattle, Goat | Domesticated animal | Artiodactyla | Indirect ELISA, Neutralisation test | Antibodies | Serum |
| 134 | Ndiaye | 2018 | Cross-sectional | Non probabilistic | Capture | Multicenter | Prospetively | Senegal | 2012-2013 | Not applicable | Unclear/ Not reported | Community-based | Not applicable | Mosquitoes |  |  |  | Culture | Live virus | Mosquitoes |
| 135 | Ngoshe | 2020 | Cross-sectional | Probabilistic | Simple random sampling | Monocenter | Prospetively | South Africa | 2015–2016 | Not applicable | Unclear/ Not reported | Community-based | Not applicable | Other animal species | Cattle, Sheep, Goat | Domesticated animal | Artiodactyla | Indirect ELISA | Antibodies | Serum |
| 136 | Nguku | 2010 | Community outbreak | Non probabilistic | Consecutive sampling | Multicenter | Prospetively | Kenya | 2006–2007 | Unclear/ Not reported | Unclear/ Not reported | Community-based | Not applicable | Humans |  |  |  | Indirect ELISA, Real Time RT-PCR | IgG, IgM, Viral RNA | Serum |
| 137 | Njenga | 2009 | Cross-sectional | Non probabilistic | Consecutive sampling | Multicenter | Prospetively | Kenya | Unclear/ Not reported | Unclear/ Not reported | Unclear/ Not reported | Community-based | Unclear/ Not reported | Humans |  |  |  | Indirect ELISA, Classical RT-PCR | IgM, Viral RNA | Serum |
| 138 | Ochieng | 2015 | Cross-sectional | Probabilistic | Cluster sampling | Nationally representative (as stated by authors) | Retrospectively | Kenya | 2007 | Adults | Urban/rural | Community-based | Not applicable | Humans |  |  |  | Indirect ELISA | IgG | Serum |
| 139 | Odaibo | 2019 | Cross-sectional | Probabilistic | Simple random sampling | Multicenter | Prospetively | Democratic Republic of the Congo | Nov/2013-Dec/2013 | Not applicable | Unclear/ Not reported | Community-based | Not applicable | Other animal species | Cattle | Domesticated animal | Artiodactyla | Indirect ELISA | IgG, IgM | Serum |
| 140 | O'Hearn | 2016 | Cross-sectional | Non probabilistic | Consecutive sampling | Monocenter | Prospetively | Sierra Leone | 2007-2014 | Unclear/ Not reported | Unclear/ Not reported | Unclear/ Not reported | Unclear/ Not reported | Humans |  |  |  | Luminex Mag- Pix | IgG | Serum |
| 141 | Olive | 2013 | Cross-sectional | Non probabilistic | Trapping | Multicenter | Prospetively | Madagascar | Oct/2008-Mar/2010 | Not applicable | Rural | Community-based | Not applicable | Other animal species | Eliurus minor, Gymnuromys roberti, Microtus thomasi, Oryzorictes hova, Eliurus tanala, Hemicentetes semispinosus, Microgale dobsoni, Rattus rattus, Eliurus majori, Microgale soricoides, Nesomys rufus | Wild animal | Rodentia, Afrosoricida | Indirect ELISA | IgG | Serum |
| 142 | Opayele | 2018 | Cross-sectional | Non probabilistic | Consecutive sampling | Multicenter | Prospetively | Nigeria | Oct/2016-Apr/2017 | Adults | Urban | Community-based | Not applicable | Humans |  |  |  | Indirect ELISA | Antibodies | Serum |
| 143 | Opayele | 2019 | Cross-sectional | Non probabilistic | Consecutive sampling | Monocenter | Prospetively | Nigeria | Jan/2017-Feb/2017 | Not applicable | Urban | Community-based | Not applicable | Other animal species | Cattle, Goat | Domesticated animal | Artiodactyla | Indirect ELISA | IgM | Serum |
| 144 | Oragwa | 2022 | Cross-sectional | Non probabilistic | Consecutive sampling | Multicenter | Prospetively | Nigeria | Feb/2019-Sep/2019 | Unclear/ Not reported | Unclear/ Not reported | Community-based | Not applicable | Humans |  |  |  | Indirect ELISA | IgG | Serum |
| 145 | Owange | 2014 | Cross-sectional | Probabilistic | Simple random sampling | Multicenter | Prospetively | Kenya | Aug/2012-Jun/2013 | Not applicable | Rural | Community-based | Not applicable | Other animal species | Cattle | Domesticated animal | Artiodactyla | Indirect ELISA | Antibodies, IgM | Serum |
| 146 | Oyas | 2018 | Cross-sectional | Non probabilistic | Consecutive sampling | Multicenter | Prospetively | Kenya | Nov/2015-Feb/2016 | Not applicable | Rural | Community-based | Not applicable | Other animal species | Cattle, Sheep, Goat | Domesticated animal | Artiodactyla | Indirect ELISA | IgG | Serum |
| 147 | Paweska | 2003 | Cross-sectional | Non probabilistic | Consecutive sampling | Multicenter | Prospetively | Kenya, Somalia, South Africa, Tanzania, Uganda | 1997–1998 | Not applicable | Unclear/ Not reported | Community-based | Not applicable | Other animal species | Cattle, Sheep, Goat | Domesticated animal | Artiodactyla | Neutralization test | IgG | Serum |
| 148 | Paweska | 2003 | Cross-sectional | Non probabilistic | Consecutive sampling | Multicenter | Prospetively | Kenya, Senegal, Somalia, South Africa,Tanzania | 1997–1998 | Not applicable | Unclear/ Not reported | Community-based | Not applicable | Other animal species | Sheep, Goat, Cattle, Buffalo, Wildebeest, Kudu, Eland | Domesticated animal | Artiodactyla | Neutralization test | IgG | Serum |
| 149 | Paweska | 2005 | Cross-sectional | Non probabilistic | Consecutive sampling | Multicenter | Prospetively | Kenya, South Africa,Tanzania, Uganda | 1974; 1997–1998; 1999–2003 | Unclear/ Not reported | Unclear/ Not reported | Unclear/ Not reported | Unclear/ Not reported | Humans |  |  |  | Neutralization test | IgG | Serum |
| 150 | Paweska | 2005 | Cross-sectional | Non probabilistic | Consecutive sampling | Multicenter | Prospetively | Ethiopia, Kenya, Somalia, South Africa, Tanzania, Uganda | 1997–1998; 2001–2003 | Unclear/ Not reported | Unclear/ Not reported | Unclear/ Not reported | Unclear/ Not reported | Humans, other animal species | Cattle, Goat, Sheep, Buffalo, Camel | Wild animal, Domesticated animal | Artiodactyla | Neutralization test | Antibodies | Serum |
| 151 | Paweska | 2007 | Cross-sectional | Non probabilistic | Consecutive sampling | Multicenter | Prospetively | Kenya, South Africa, Tanzania, Uganda, Zimbabwe | 1997–1998; 1999–2005 | Unclear/ Not reported | Unclear/ Not reported | Unclear/ Not reported | Unclear/ Not reported | Humans |  |  |  | Neutralization test | IgG | Serum |
| 152 | Paweska | 2008 | Cross-sectional | Non probabilistic | Consecutive sampling | Multicenter | Prospetively | Kenya, South Africa | 2002–2007 | Not applicable | Unclear/ Not reported | Community-based | Not applicable | Other animal species | Buffalo | Wild animal | Artiodactyla | Neutralization test | IgG | Serum |
| 153 | Pawęska | 2021 | Cross-sectional | Non probabilistic | Consecutive sampling | Multicenter | Prospetively | South Africa | Apr/2018–Aug/2019 | All ages | Urban | Hospital-based | Ambulatory | Humans |  |  |  | Indirect ELISA | IgG, IgM | Serum |
| 154 | Peterson | 2017 | Cross-sectional | Probabilistic | Stratified sampling | Multicenter | Prospetively | Madagascar | Mar/2014-May/2014 | Not applicable | Unclear/ Not reported | Community-based | Not applicable | Other animal species | Cattle | Domesticated animal | Artiodactyla | Indirect ELISA | Antibodies | Serum |
| 155 | Poueme | 2019 | Cross-sectional | Probabilistic | Multistage sampling | Multicenter | Prospetively | Cameroon | Jan/2016–Jan/2017 | Not applicable | Unclear/ Not reported | Community-based | Not applicable | Other animal species | Goat, sheep | Domesticated animal | Artiodactyla | Indirect ELISA | IgG | Serum |
| 156 | Pourrut | 2010 | Cross-sectional | Probabilistic | Simple random sampling | Multicenter | Prospetively | Gabon | 2005-2008 | Adults | Rural | Community-based | Not applicable | Humans |  |  |  | Indirect ELISA | IgG | Serum |
| 157 | Rakotoarivelo | 2011 | Cross-sectional | Non probabilistic | Convenience sampling | Multicenter | Retrospectively | Madagascar | jan/2008-jul/2008 | Adults | Urban | Hospital-based | Hospitalized | Humans |  |  |  | Classical RT-PCR | Viral RNA | Serum |
| 158 | Ratovonjato | 2011 | Cross-sectional | Non probabilistic | Capture | Multicenter | Prospetively | Madagascar | Dec/2008; Apr/2009 | Not applicable | Urban/rural | Community-based | Not applicable | Mosquitoes |  |  |  | Real Time RT-PCR | Viral RNA | Mosquitoes |
| 159 | Ringot | 2004 | Cross-sectional | Probabilistic | Simple random sampling | Multicenter | Prospetively | Chad | Aug/2002-Oct/2002 | Not applicable | Urban | Community-based | Not applicable | Other animal species | Cattle, Sheep, Goat | Domesticated animal | Artiodactyla | Indirect ELISA | IgG | Serum |
| 160 | Rissmann | 2017 | Cross-sectional | Non probabilistic | Consecutive sampling | Multicenter | Prospetively | Mauritania | 2012-2013 | Not applicable | Unclear/ Not reported | Community-based | Not applicable | Other animal species | Cattle, Camel, Small ruminants (Goat and Sheep) | Domesticated animal | Artiodactyla | Indirect ELISA | IgG | Serum |
| 161 | Rissmann | 2017 | Cross-sectional | Probabilistic | Simple random sampling | Multicenter | Prospetively | Cameroon | 2013-2014 | Not applicable | Unclear/ Not reported | Community-based | Not applicable | Other animal species | Cattle, Small ruminants (Goat and Sheep) | Domesticated animal | Artiodactyla | Indirect ELISA | IgG | Serum |
| 162 | Roger | 2011 | Cross-sectional | Probabilistic | Simple random sampling | Multicenter | Prospetively | Comoros | Apr/2009–Aug/2009 | Not applicable | Unclear/ Not reported | Community-based | Not applicable | Other animal species | Cattle, Sheep, Goat | Domesticated animal | Artiodactyla | Indirect ELISA | IgG | Serum |
| 163 | Roger | 2014 | Cross-sectional | Non probabilistic | Consecutive sampling | Multicenter | Prospetively | Comoros | Apr/2010-Aug/2011 | Not applicable | Unclear/ Not reported | Community-based | Not applicable | Other animal species, Mosquitoes | Livestock (goat, sheep, cattle) | Domesticated animal | Artiodactyla | Indirect ELISA, Real Time RT-PCR | IgG, Viral RNA | Serum, Mosquitoes |
| 164 | Rostal | 2010 | Cross-sectional | Non probabilistic | Consecutive sampling | Multicenter | Prospetively | Kenya | juil-06 | Not applicable | Unclear/ Not reported | Community-based | Not applicable | Other animal species | Goat, sheep | Domesticated animal | Artiodactyla | Indirect ELISA | IgG | Serum |
| 165 | Roug | 2020 | Cross-sectional | Non probabilistic | Consecutive sampling | Multicenter | Prospetively | Tanzania | 2014-2017 | Not applicable | Unclear/ Not reported | Community-based | Not applicable | Other animal species | Buffalo | Wild animal | Artiodactyla | Indirect ELISA, Real Time RT-PCR | IgG, Viral RNA | Serum |
| 166 | Rugarabamu | 2022 | Cross-sectional | Probabilistic | Simple random sampling | Multicenter | Prospetively | Tanzania | Jun/2018-Nov/2018 | Unclear/ Not reported | Urban | Hospital-based | Ambulatory | Humans |  |  |  | Indirect ELISA | IgM | Serum |
| 167 | Sadeuh-Mba | 2018 | Cross-sectional | Probabilistic | Simple random sampling | Multicenter | Prospetively | Cameroon | 2005-2012 | Unclear/ Not reported | Rural | Community-based | Not applicable | Humans |  |  |  | Indirect ELISA | IgG | Serum |
| 168 | Salekwa | 2019 | Cross-sectional | Non probabilistic | Consecutive sampling | Multicenter | Prospetively | Tanzania | Sep/2015-May/2016 | Not applicable | Rural | Community-based | Not applicable | Other animal species | Cattle | Domesticated animal | Artiodactyla | Indirect ELISA | IgG, IgM | Serum |
| 169 | Sanderson | 2020 | Cross-sectional | Non probabilistic | Consecutive sampling | Multicenter | Prospetively | Botswana | 2013–2014 | All ages | Urban | Hospital-based | Unclear/ Not reported | Humans |  |  |  | Indirect ELISA | IgG, IgM | Serum |
| 170 | Sang | 2010 | Cross-sectional | Non probabilistic | Capture | Multicenter | Prospetively | Kenya | Jan/2007-Feb/2007 | Not applicable | Rural | Community-based | Not applicable | Mosquitoes |  |  |  | Classical RT-PCR | Viral RNA | Mosquitoes |
| 171 | Schoepp | 2014 | Cross-sectional | Non probabilistic | Consecutive sampling | Multicenter | Prospetively | Sierra Leone, Liberia, Guinea | Oct/2006–Oct/2008 | Unclear/ Not reported | Unclear/ Not reported | Unclear/ Not reported | Unclear/ Not reported | Humans |  |  |  | Indirect ELISA | IgM | Serum |
| 172 | Schwarz | 2012 | Cross-sectional | Non probabilistic | Consecutive sampling | Multicenter | Prospetively | Madagascar | May/2010–Jul/2010 | Adults | Urban | Hospital-based | Unclear/ Not reported | Humans |  |  |  | Indirect immunofluorescence assay | IgG | Serum |
| 173 | Selmi | 2020 | Cross-sectional | Probabilistic | Simple random sampling | Multicenter | Prospetively | Tunisia | Jan/2017-Dec/2018 | Not applicable | Unclear/ Not reported | Community-based | Not applicable | Other animal species | Camel | Domesticated animal | Artiodactyla | Indirect ELISA | IgG | Serum |
| 174 | Shieh | 2010 | Community outbreak | Non probabilistic | Convenience sampling | Multicenter | Prospetively | Kenya, Tanzania, Somalia | Dec/2006–Jan/2007 | Unclear/ Not reported | Unclear/ Not reported | Unclear/ Not reported | Unclear/ Not reported | Humans, other animal species | Cow, Sheep | Domesticated animal | Artiodactyla | Immunohistochemical assays | Viral antigen | Organ tissue |
| 175 | Sindato | 2013 | Cross-sectional | Probabilistic | Multistage sampling | Multicenter | Retrospectively | Tanzania | Jun/2002-Sep/2007 | Not applicable | Rural | Community-based | Not applicable | Other animal species | Sheep, Goat, Cattle, Gazelle, Elephant, Wildebeest, Lion, Buffalo, Zebra | Wild animal, Domesticated animal | Artiodactyla, Proboscidea, Carnivora, Perissodactyla | Indirect ELISA | IgG | Serum |
| 176 | Sindato | 2015 | Cross-sectional | Probabilistic | Simple random sampling | Multicenter | Prospetively | Tanzania | Apr/2013-Aug/2013 | Not applicable | Rural | Community-based | Not applicable | Other animal species | Cattle, Sheep, Goat | Domesticated animal | Artiodactyla | Indirect ELISA | Antibodies | Serum |
| 177 | Soumare | 2007 | Cross-sectional | Non probabilistic | Consecutive sampling | Multicenter | Prospetively | Somalia | Aug/2001-Jul/2004 | Not applicable | Unclear/ Not reported | Community-based | Not applicable | Other animal species | Cattle, Camel, Goat, Sheep, Animal unspecified | Wild animal, Domesticated animal | Artiodactyla | Indirect ELISA | IgG | Serum |
| 178 | Sow | 2014 | Community outbreak | Non probabilistic | Consecutive sampling | Multicenter | Prospetively | Mauritania | Sep/2012-Nov/2012 | Unclear/ Not reported | Unclear/ Not reported | Community-based | Not applicable | Humans, Mosquitoes |  |  |  | Immunofluorescent assay, Classical RT-PCR, Indirect ELISA, Culture | IgG, IgM, Viral RNA, Live virus | Serum, Mosquitoes |
| 179 | Sow | 2014 | Cross-sectional | Non probabilistic | Consecutive sampling | Multicenter | Prospetively | Senegal | oct-12 | Unclear/ Not reported | Unclear/ Not reported | Community-based | Not applicable | Humans |  |  |  | Indirect ELISA, Classical RT-PCR | IgG, IgM, Viral RNA | Serum |
| 180 | Sow | 2016 | Cross-sectional | Non probabilistic | Consecutive sampling | Multicenter | Prospetively | Senegal | Sep/2013-Oct/2013 | Unclear/ Not reported | Unclear/ Not reported | Community-based | Not applicable | Humans, other animal species, Mosquitoes | Cattle, Sheep, Goat | Domesticated animal | Artiodactyla | Indirect ELISA, Classical RT-PCR, Culture | IgG, IgM, Viral RNA, Live virus | Serum, Mosquitoes |
| 181 | Sow | 2016 | Cross-sectional | Non probabilistic | Consecutive sampling | Multicenter | Prospetively | Senegal | Jul/2009-Mar/2013 | All ages | Unclear/ Not reported | Hospital-based | Unclear/ Not reported | Humans |  |  |  | Indirect ELISA, Real Time RT-PCR | IgM, Viral RNA | Serum |
| 182 | Spiropoulou | 2018 | Cross-sectional | Non probabilistic | Convenience sampling | Multicenter | Prospetively | Uganda | Apr/2016 | All ages | Unclear/ Not reported | Community-based | Not applicable | Humans, other animal species | Cattle, Sheep, Goat | Domesticated animal | Artiodactyla | Indirect ELISA | IgG, IgM, Antibodies | Serum |
| 183 | Sternberg Lewerin | 2018 | Cross-sectional | Probabilistic | Simple random sampling | Multicenter | Prospetively | Uganda | Jan/2015-Mar/2015 | Not applicable | Unclear/ Not reported | Community-based | Not applicable | Other animal species | Cattle | Domesticated animal | Artiodactyla | Indirect ELISA | Antibodies | Serum |
| 184 | Stoek | 2022 | Cross-sectional | Non probabilistic | Consecutive sampling | Multicenter | Prospetively | Mauritania | 2018 | Unclear/ Not reported | Urban/rural | Community-based | Not applicable | Other animal species, Mosquitoes | Cattle | Domesticated animal | Artiodactyla | Indirect ELISA, Real Time RT-PCR | IgM, Viral RNA | Serum, Mosquitoes |
| 185 | Sumaye | 2013 | Cross-sectional | Probabilistic | Simple random sampling | Multicenter | Prospetively | Tanzania | May/2011-Aug/2011 | Not applicable | Rural | Community-based | Not applicable | Other animal species | Cattle, Sheep, Goat | Domesticated animal | Artiodactyla | Indirect ELISA | IgM, Antibodies | Serum |
| 186 | Sumaye | 2015 | Cross-sectional | Probabilistic | Simple random sampling | Multicenter | Prospetively | Tanzania | Mar/2012-Aug/2012 | All ages | Rural | Community-based | Not applicable | Humans |  |  |  | Indirect ELISA | IgM, Antibodies | Serum |
| 187 | Swai | 2009 | Cross-sectional | Non probabilistic | Consecutive sampling | Multicenter | Prospetively | Tanzania | 2004 | Adults | Unclear/ Not reported | Community-based | Not applicable | Humans |  |  |  | Indirect ELISA | Antibodies | Serum |
| 188 | Swai | 2015 | Cross-sectional | Non probabilistic | Consecutive sampling | Multicenter | Prospetively | Tanzania | Jun/2010-Aug/2010 | Not applicable | Unclear/ Not reported | Community-based | Not applicable | Other animal species | Camel | Domesticated animal | Artiodactyla | Indirect ELISA | IgG | Serum |
| 189 | Tigoi | 2015 | Cross-sectional | Non probabilistic | Consecutive sampling | Multicenter | Prospetively | Kenya | Sep/2009-Dec/2012 | All ages | Unclear/ Not reported | Hospital-based | Unclear/ Not reported | Humans |  |  |  | Indirect ELISA | IgG, IgM | Serum |
| 190 | Tigoi | 2020 | Cross-sectional | Probabilistic | Simple random sampling | Multicenter | Prospetively | Kenya | Aug/2014-Nov/2015 | Adults | Unclear/ Not reported | Community-based | Not applicable | Humans |  |  |  | Indirect ELISA | IgG, IgM | Serum |
| 191 | Traoré-Lamizana | 2001 | Cross-sectional | Non probabilistic | Capture | Multicenter | Prospetively | Senegal | Oct/1990-Dec/1995 | Not applicable | Unclear/ Not reported | Community-based | Not applicable | Mosquitoes |  |  |  | Culture | Live virus | Mosquitoes |
| 192 | Troupin | 2022 | Cross-sectional | Probabilistic | Simple random sampling | Multicenter | Prospetively | Guinea | Oct/2017-Jun/2019 | Unclear/ Not reported | Unclear/ Not reported | Community-based | Not applicable | Other animal species | Cattle, Sheep, Goat | Domesticated animal | Artiodactyla | Indirect ELISA | IgG | Serum |
| 193 | Tshilenge | 2019 | Cross-sectional | Probabilistic | Simple random sampling | Multicenter | Prospetively | Democratic Republic of the Congo | 2013-2015 | Not applicable | Unclear/ Not reported | Community-based | Not applicable | Other animal species | Goat, sheep | Domesticated animal | Artiodactyla | Indirect ELISA | IgG | Serum |
| 194 | Umuhoza | 2017 | Cross-sectional | Non probabilistic | Consecutive sampling | Multicenter | Prospetively | Rwanda | Dec/2012-Mar/2013 | Not applicable | Unclear/ Not reported | Community-based | Not applicable | Other animal species | Cattle | Domesticated animal | Artiodactyla | Indirect ELISA | Antibodies | Serum |
| 195 | Ushijima | 2021 | Cross-sectional | Non probabilistic | Consecutive sampling | Monocenter | Prospetively | Gabon | Nov/2014-Mar/2020 | Unclear/ Not reported | Urban | Hospital-based | Ambulatory | Humans |  |  |  | Indirect ELISA, Real Time RT-PCR | IgG, Viral RNA | Serum |
| 196 | van den Bergh | 2022 | Cross-sectional | Non probabilistic | Consecutive sampling | Multicenter | Prospetively | South Africa | 2017–2018 | Unclear/ Not reported | Unclear/ Not reported | Community-based | Not applicable | Mosquitoes |  |  |  | Real Time RT-PCR | Viral RNA | Mosquitoes |
| 197 | Van den Bergh | 2020 | Cross-sectional | Non probabilistic | Consecutive sampling | Multicenter | Prospetively | South Africa | Jun/2016-May/2018. | Not applicable | Unclear/ Not reported | Community-based | Not applicable | Other animal species | Nyala, Impala | Wild animal | Artiodactyla | Neutralization test | Antibodies | Serum |
| 198 | Weber | 2018 | Cross-sectional | Probabilistic | Systematic sampling | Multicenter | Prospetively | Kenya | Sep/2013-Mar/2014 | Unclear/ Not reported | Unclear/ Not reported | Community-based | Not applicable | Humans, other animal species | Cattle, Sheep, Goat | Domesticated animal | Artiodactyla | Indirect ELISA | IgG | Serum |
| 199 | Wensman | 2015 | Cross-sectional | Non probabilistic | Consecutive sampling | Multicenter | Prospetively | Tanzania | Sep/2014–Nov/2014 | Not applicable | Unclear/ Not reported | Community-based | Not applicable | Other animal species | Small ruminants (Goat and Sheep) | Domesticated animal | Artiodactyla | Indirect ELISA | Antibodies | Serum |
| 200 | Wolff | 2018 | Cross-sectional | Non probabilistic | Consecutive sampling | Multicenter | Prospetively | South Africa | Oct/2012-Jun/2013 | Unclear/ Not reported | Unclear/ Not reported | Hospital-based | Unclear/ Not reported | Humans |  |  |  | Hemagglutination inhibition test | Antibodies | Serum |
| 201 | Woods | 2002 | Community outbreak | Probabilistic | Multistage sampling | Multicenter | Prospetively | Kenya | Oct/1997-Feb/1998 | All ages | Unclear/ Not reported | Community-based | Not applicable | Humans |  |  |  | Indirect ELISA | IgG, IgM | Serum |
| 202 | Youssef | 2001 | Cross-sectional | Non probabilistic | Capture | Monocenter | Prospetively | Egypt | Unclear/ Not reported | Not applicable | Unclear/ Not reported | Community-based | Not applicable | Other animal species, Mosquitoes | Rattus rattus | Wild animal | Rodentia | Indirect ELISA, Classical RT-PCR | Antibobies, Viral RNA | Mosquitoes |
| 203 | Youssef | 2002 | Cross-sectional | Non probabilistic | Consecutive sampling | Multicenter | Prospetively | Egypt | Unclear/ Not reported | Not applicable | Unclear/ Not reported | Community-based | Not applicable | Other animal species | Rattus rattus | Wild animal | Rodentia | Classical RT-PCR | Viral RNA | Serum |
| 204 | Youssef | 2009 | Cross-sectional | Non probabilistic | Consecutive sampling | Multicenter | Prospetively | Egypt | Unclear/ Not reported | Unclear/ Not reported | Unclear/ Not reported | Community-based | Not applicable | Humans, other animal species | Pig | Domesticated animal | Artiodactyla | Indirect ELISA | Antibodies | Serum |
| 205 | Zouaghi | 2021 | Cross-sectional | Probabilistic | Simple random sampling | Monocenter | Retrospectively | Tunisia | 2011-2014 | Unclear/ Not reported | Unclear/ Not reported | Community-based | Not applicable | Other animal species | Cattle, Sheep, Goat | Domesticated animal | Artiodactyla | Indirect ELISA | IgG | Serum |

# S4 Table. Risk of bias assessment

| N° | Author | Year of publication | Was the study’s target population a close representation of the national population in relation to RVFVprevalence? | Was the sampling frame a true or close representation of the target population? | Was some form of random selection used to select the sample, OR was a census undertaken? | Were data collected directly from the subjects (as opposed to a proxy)? | Was an acceptable inclusion criteria used in the study? | Was the length of the study period > or = 1 year? | Was the response rate ≥ 70 % or not significant different in relevant demographic characteristics between responders and nonresponders? | Was the same mode of data collection used for all subjects? | Was the RVFV detection assay shown to have reliability and validity? | Were the numerator(s) and denominator(s) for the RVFV prevalence or case fatality rate appropriate? | Risk of bias |
| --- | --- | --- | --- | --- | --- | --- | --- | --- | --- | --- | --- | --- | --- |
| 1 | Abakar | 2014 | No | Yes | Yes | Not applicable | Yes | Unclear | No | Yes | Yes | Yes | Moderate risk of bias |
| 2 | Abdallah | 2016 | No | Yes | Yes | Not applicable | Yes | No | No | Yes | Yes | Yes | Moderate risk of bias |
| 3 | Adamu | 2020 | No | Yes | Yes | Not applicable | Yes | No | No | Yes | Yes | Yes | Moderate risk of bias |
| 4 | Adamu | 2021 | No | Yes | Yes | Not applicable | Yes | No | Unclear | Yes | Yes | Yes | Moderate risk of bias |
| 5 | Adesiyun | 2020 | No | Yes | Yes | Not applicable | Yes | Unclear | No | Yes | Yes | Yes | Moderate risk of bias |
| 6 | Ahmed | 2018 | No | Yes | No | Yes | Yes | No | No | Yes | Yes | Yes | Moderate risk of bias |
| 7 | Ahmed | 2000 | No | Yes | Yes | Yes | Yes | No | Yes | Yes | Yes | Yes | Low risk of bias |
| 8 | Alhaji | 2020 | No | Yes | Yes | Not applicable | Yes | Yes | No | Yes | Yes | Yes | Low risk of bias |
| 9 | Andayi | 2014 | No | Yes | Yes | Yes | Yes | No | Yes | Yes | Yes | Yes | Low risk of bias |
| 10 | Andriamandimby | 2010 | No | Yes | Yes | Yes | Yes | Unclear | Yes | Yes | Yes | Yes | Low risk of bias |
| 11 | Andriamandimby | 2018 | No | Yes | No | Not applicable | Yes | Yes | No | Yes | Yes | Yes | Moderate risk of bias |
| 12 | Anyangu | 2010 | No | Yes | No | Yes | Yes | No | Unclear | Yes | Yes | Yes | Moderate risk of bias |
| 13 | Aradaib | 2013 | No | Yes | Yes | Yes | Yes | Yes | Yes | Yes | Yes | Yes | Low risk of bias |
| 14 | Archer | 2011 | No | Yes | Yes | Yes | Yes | Yes | Yes | Yes | Yes | Yes | Low risk of bias |
| 15 | Archer | 2013 | No | Yes | No | Yes | Yes | Yes | Unclear | Yes | Yes | Yes | Low risk of bias |
| 16 | Atuman | 2022 | No | Yes | Yes | Not applicable | Yes | Yes | Unclear | Yes | Yes | Yes | Low risk of bias |
| 17 | Ayari-Fakhfakh | 2011 | No | Yes | No | Not applicable | Yes | No | No | Yes | Yes | Yes | Moderate risk of bias |
| 18 | Ba | 2012 | No | Yes | No | Not applicable | Yes | No | No | Yes | Yes | Yes | Moderate risk of bias |
| 19 | Baudin | 2016 | No | Yes | Yes | Yes | Yes | Yes | Yes | Yes | Yes | Yes | Low risk of bias |
| 20 | Beechler | 2015 | No | Yes | Yes | Not applicable | Yes | Yes | No | Yes | Yes | Yes | Low risk of bias |
| 21 | Bett | 2019 | No | Yes | Yes | Yes | Yes | No | Yes | Yes | Yes | Yes | Low risk of bias |
| 22 | Bird | 2008 | No | Yes | No | Not applicable | Yes | No | No | No | Yes | Yes | Moderate risk of bias |
| 23 | Bisimwa | 2015 | No | Yes | No | Not applicable | Yes | No | No | Yes | Yes | Yes | Moderate risk of bias |
| 24 | Blomstrom | 2016 | No | Yes | Yes | Not applicable | Yes | No | No | Yes | Yes | Yes | Moderate risk of bias |
| 25 | Bob | 2017 | No | Yes | Yes | Yes | Yes | No | Yes | Yes | Yes | Yes | Low risk of bias |
| 26 | Bob | 2022 | No | Yes | No | Yes | Yes | Yes | No | Yes | Yes | Yes | Low risk of bias |
| 27 | Bonney | 2013 | No | Yes | Yes | Not applicable | Yes | Yes | Yes | Yes | Yes | Yes | Low risk of bias |
| 28 | Bosworth | 2016 | No | Yes | Yes | Yes | Yes | Unclear | Yes | Yes | Yes | Yes | Low risk of bias |
| 29 | Boushab | 2015 | No | Yes | No | Yes | Yes | No | Yes | Yes | Yes | Yes | Low risk of bias |
| 30 | Boushab | 2016 | No | Yes | Yes | Yes | Yes | No | Yes | Yes | Yes | Yes | Low risk of bias |
| 31 | Boussini | 2014 | No | Yes | No | Not applicable | Yes | Unclear | No | Yes | Yes | Yes | Moderate risk of bias |
| 32 | Boussini | 2014 | No | Yes | No | Not applicable | Yes | Unclear | No | Yes | Yes | Yes | Moderate risk of bias |
| 33 | Bronsvoort | 2022 | No | Yes | Yes | Not applicable | Yes | No | Yes | Yes | Yes | Yes | Low risk of bias |
| 34 | Budasha | 2018 | No | Yes | Yes | Not applicable | Yes | Unclear | No | Yes | Yes | Yes | Moderate risk of bias |
| 35 | Budodo | 2020 | No | Yes | No | Yes | Yes | No | No | Yes | Yes | Yes | Moderate risk of bias |
| 36 | Bukbuk | 2014 | No | Yes | Yes | Yes | Yes | No | Yes | Yes | Yes | Yes | Low risk of bias |
| 37 | Capobianco Dondona | 2016 | No | Yes | Yes | Not applicable | Yes | No | No | Yes | Yes | Yes | Moderate risk of bias |
| 38 | Centers for Disease Control and Prevention (CDC) | 2007 | No | Yes | No | Yes | Yes | No | Unclear | Yes | Yes | Yes | Moderate risk of bias |
| 39 | Chambaro | 2022 | No | Yes | Yes | Not applicable | Yes | No | Unclear | Yes | Yes | Yes | Moderate risk of bias |
| 40 | Chengula | 2014 | No | Yes | No | Not applicable | Yes | No | No | Yes | Yes | Yes | Moderate risk of bias |
| 41 | Chevalier | 2005 | No | Yes | No | Not applicable | Yes | Unclear | No | Yes | Yes | Yes | Moderate risk of bias |
| 42 | Chevalier | 2011 | No | Yes | Yes | Not applicable | Yes | No | No | Yes | Yes | Yes | Moderate risk of bias |
| 43 | Cichon | 2021 | No | Yes | No | Not applicable | Yes | No | Unclear | Yes | Yes | Yes | Moderate risk of bias |
| 44 | Clements | 2019 | No | Yes | No | Yes | Yes | Yes | Unclear | Yes | Yes | Yes | Low risk of bias |
| 45 | Cook | 2017 | No | Yes | Yes | Yes | Yes | Yes | Unclear | Yes | Yes | Yes | Low risk of bias |
| 46 | Cosseddu | 2021 | No | Yes | Yes | Not applicable | Yes | No | No | Yes | Yes | Yes | Moderate risk of bias |
| 47 | Di Nardo | 2014 | No | Yes | No | Not applicable | Yes | No | Unclear | Yes | Yes | Yes | Moderate risk of bias |
| 48 | Diallo | 2000 | No | Yes | No | Not applicable | Yes | Unclear | Unclear | Yes | Yes | Yes | Moderate risk of bias |
| 49 | Diallo | 2005 | No | Yes | No | Not applicable | Yes | No | Unclear | Yes | Yes | Yes | Moderate risk of bias |
| 50 | Dione | 2022 | No | Yes | Yes | Not applicable | Yes | No | Yes | Yes | Yes | Yes | Low risk of bias |
| 51 | Durand | 2003 | No | Yes | No | Yes | Yes | No | Unclear | Yes | Yes | Yes | Moderate risk of bias |
| 52 | Durand | 2020 | No | Yes | Yes | Not applicable | Yes | No | Unclear | Yes | Yes | Yes | Moderate risk of bias |
| 53 | Dutuze | 2020 | No | Yes | No | Not applicable | Yes | No | Unclear | Yes | Yes | Yes | Moderate risk of bias |
| 54 | Ebogo-Belobo | 2022 | No | Yes | No | Not applicable | Yes | No | No | Yes | Yes | Yes | Moderate risk of bias |
| 55 | Eckstein | 2022 | No | Yes | No | Not applicable | Yes | No | No | Yes | Yes | Yes | Moderate risk of bias |
| 56 | El Bahgy | 2018 | No | Yes | No | Not applicable | Yes | Unclear | No | Yes | Yes | Yes | Moderate risk of bias |
| 57 | El Mamy | 2011 | No | Yes | No | Not applicable | Yes | No | No | Yes | Yes | Yes | Moderate risk of bias |
| 58 | El Mamy | 2010 | No | Yes | No | Not applicable | Yes | No | No | Yes | Yes | Yes | Moderate risk of bias |
| 59 | El-Harrak | 2011 | No | Yes | No | Not applicable | Yes | Unclear | No | Yes | Yes | Yes | Moderate risk of bias |
| 60 | Endale | 2021 | No | Yes | No | Not applicable | Yes | No | No | Yes | Yes | Yes | Moderate risk of bias |
| 61 | Enem | 2020 | No | Yes | No | Yes | Yes | No | Yes | Yes | Yes | Yes | Low risk of bias |
| 62 | EVANS | 2008 | No | Yes | No | Not applicable | Yes | Yes | No | Yes | Yes | Yes | Moderate risk of bias |
| 63 | Fafetine | 2012 | No | Yes | No | Not applicable | Yes | Unclear | Yes | Yes | Yes | Yes | Moderate risk of bias |
| 64 | Fafetine | 2013 | No | Yes | Yes | Not applicable | Yes | No | Yes | Yes | Yes | Yes | Low risk of bias |
| 65 | Fafetine | 2014 | No | Yes | No | Not applicable | Yes | No | Yes | Yes | Yes | Yes | Moderate risk of bias |
| 66 | Fagbo | 2014 | No | Yes | No | Not applicable | Yes | Unclear | Yes | Yes | Yes | Yes | Moderate risk of bias |
| 67 | Faye | 2007 | No | Yes | No | Yes | Yes | No | Yes | Yes | Yes | Yes | Low risk of bias |
| 68 | Faye | 2007 | No | Yes | No | Yes | Yes | No | Yes | Yes | Yes | Yes | Low risk of bias |
| 69 | Fischer-Tenhagen | 2000 | No | Yes | No | Not applicable | Yes | Yes | Yes | Yes | Yes | Yes | Low risk of bias |
| 70 | Fokam | 2010 | No | Yes | No | Yes | Yes | Unclear | Yes | Yes | Yes | Yes | Low risk of bias |
| 71 | Georges | 2018 | No | Yes | Yes | Not applicable | Yes | No | No | Yes | Yes | Yes | Moderate risk of bias |
| 72 | Gora | 2000 | No | Yes | No | Not applicable | Yes | Yes | No | Yes | Yes | Yes | Moderate risk of bias |
| 73 | Gray | 2015 | No | Yes | No | Yes | Yes | Yes | Yes | Yes | Yes | Yes | Low risk of bias |
| 74 | Grolla | 2012 | No | Yes | No | Yes | Yes | No | No | Yes | Yes | Yes | Moderate risk of bias |
| 75 | Grossi-soyster | 2017 | No | Yes | No | Yes | Yes | Yes | Yes | Yes | Yes | Yes | Low risk of bias |
| 76 | Gudo | 2016 | No | Yes | No | Yes | Yes | No | Yes | Yes | Yes | Yes | Low risk of bias |
| 77 | Gudo | 2016 | No | Yes | No | Yes | Yes | No | No | Yes | Yes | Yes | Moderate risk of bias |
| 78 | Guillebaud | 2018 | No | Yes | Yes | Yes | Yes | No | Yes | Yes | Yes | Yes | Low risk of bias |
| 79 | Halawi | 2019 | No | Yes | Yes | Not applicable | Yes | Unclear | No | Yes | Yes | Yes | Moderate risk of bias |
| 80 | Hanafi | 2011 | No | Yes | No | Not applicable | Yes | No | Yes | Yes | Yes | Yes | Moderate risk of bias |
| 81 | Hassan | 2020 | No | Yes | Yes | Yes | Yes | No | Yes | Yes | Yes | Yes | Low risk of bias |
| 82 | Hassanain | 2010 | No | Yes | No | Yes | Yes | No | Yes | Yes | Yes | Yes | Low risk of bias |
| 83 | Hassine | 2017 | No | Yes | No | Not applicable | Yes | Unclear | No | Yes | Yes | Yes | Moderate risk of bias |
| 84 | Heinrich | 2012 | No | Yes | Yes | Yes | Yes | Yes | Yes | Yes | Yes | Yes | Low risk of bias |
| 85 | Horton | 2014 | No | Yes | No | Not applicable | Yes | No | No | Yes | Yes | Yes | Moderate risk of bias |
| 86 | Ibrahim | 2021 | No | Yes | Yes | Yes | Yes | No | Yes | Yes | Yes | Yes | Low risk of bias |
| 87 | Jäckel | 2013 | No | Yes | No | Not applicable | Yes | No | No | Yes | Yes | Yes | Moderate risk of bias |
| 88 | Jeanmaire | 2011 | No | Yes | No | Not applicable | Yes | No | No | Yes | Yes | Yes | Moderate risk of bias |
| 89 | Jori | 2015 | No | Yes | No | Not applicable | Yes | Unclear | No | Yes | Yes | Yes | Moderate risk of bias |
| 90 | Kading | 2018 | No | Yes | No | Not applicable | Yes | Yes | No | Yes | Yes | Yes | Moderate risk of bias |
| 91 | Kanoute | 2017 | No | Yes | Yes | Not applicable | Yes | No | Yes | Yes | Yes | Yes | Low risk of bias |
| 92 | Kifaro | 2014 | No | Yes | No | Not applicable | Yes | No | Yes | Yes | Yes | Yes | Moderate risk of bias |
| 93 | LaBeaud | 2008 | No | Yes | Yes | Yes | Yes | No | Yes | Yes | Yes | Yes | Low risk of bias |
| 94 | LaBeaud | 2011 | No | Yes | No | Not applicable | Yes | Yes | Yes | Yes | Yes | Yes | Low risk of bias |
| 95 | LaBeaud | 2011 | No | Yes | Yes | Yes | Yes | No | Yes | Yes | Yes | Yes | Low risk of bias |
| 96 | LaBeaud | 2011 | No | Yes | No | Not applicable | Yes | Yes | Yes | Yes | Yes | Yes | Low risk of bias |
| 97 | LaBeaud | 2015 | No | Yes | No | Yes | Yes | No | Yes | Yes | Yes | Yes | Low risk of bias |
| 98 | Lagare | 2019 | No | Yes | No | Yes | Yes | No | Yes | Yes | Yes | Yes | Low risk of bias |
| 99 | Lagerqvist | 2013 | No | Yes | No | Not applicable | Yes | Unclear | Yes | Yes | Yes | Yes | Moderate risk of bias |
| 100 | LeBreton | 2006 | No | Yes | No | Not applicable | Yes | Unclear | Yes | Yes | Yes | Yes | Moderate risk of bias |
| 101 | Lubisi | 2020 | No | Yes | No | Not applicable | Yes | Yes | Yes | Yes | Yes | Yes | Low risk of bias |
| 102 | Lutomiah | 2014 | No | Yes | No | Not applicable | Yes | No | Yes | Yes | Yes | Yes | Moderate risk of bias |
| 103 | Lwande | 2015 | No | Yes | No | Not applicable | Yes | Yes | Yes | Yes | Yes | Yes | Low risk of bias |
| 104 | Lysholm | 2022 | No | Yes | Yes | Not applicable | Yes | No | No | Yes | Yes | Yes | Moderate risk of bias |
| 105 | Maganga | 2017 | No | Yes | No | Not applicable | Yes | No | Yes | Yes | Yes | Yes | Moderate risk of bias |
| 106 | Magona | 2013 | No | Yes | Yes | Not applicable | Yes | Unclear | Yes | Yes | Yes | Yes | Low risk of bias |
| 107 | Mahmoud | 2018 | No | Yes | No | Not applicable | Yes | Unclear | Yes | Yes | Yes | Yes | Moderate risk of bias |
| 108 | Mahmoud | 2021 | No | Yes | No | Not applicable | Yes | Yes | No | Yes | Yes | Yes | Moderate risk of bias |
| 109 | Makiala-Mandanda | 2018 | No | Yes | No | Yes | Yes | Yes | Yes | Yes | Yes | Yes | Low risk of bias |
| 110 | Mapaco | 2012 | No | Yes | No | Not applicable | Yes | No | No | Yes | Yes | Yes | Moderate risk of bias |
| 111 | Marietou | 2019 | No | Yes | No | Not applicable | Yes | Unclear | Yes | Yes | Yes | Yes | Moderate risk of bias |
| 112 | Marrama | 2005 | No | Yes | Yes | Yes | Yes | Unclear | Yes | Yes | Yes | Yes | Low risk of bias |
| 113 | Matiko | 2018 | No | Yes | Yes | Not applicable | Yes | Yes | Yes | Yes | Yes | Yes | Low risk of bias |
| 114 | Mbotha | 2018 | No | Yes | Yes | Not applicable | Yes | No | Yes | Yes | Yes | Yes | Low risk of bias |
| 115 | Mease | 2011 | No | Yes | Yes | Yes | Yes | Unclear | Yes | Yes | Yes | Yes | Low risk of bias |
| 116 | Mhina | 2015 | No | Yes | No | Not applicable | Yes | No | Unclear | Yes | Yes | Yes | Moderate risk of bias |
| 117 | Miller | 2011 | No | Yes | Yes | Not applicable | Yes | No | Yes | Yes | Yes | Yes | Low risk of bias |
| 118 | Mohamed | 2010 | No | Yes | No | Yes | Yes | No | No | Yes | Yes | Yes | Moderate risk of bias |
| 119 | Mohamed | 2019 | No | Yes | No | Yes | Yes | No | Yes | Yes | Yes | Yes | Low risk of bias |
| 120 | Moiane | 2017 | No | Yes | No | Not applicable | Yes | No | Yes | Yes | Yes | Yes | Moderate risk of bias |
| 121 | Monaco | 2013 | No | Yes | No | Not applicable | Yes | No | No | Yes | Yes | Yes | Moderate risk of bias |
| 122 | Mordi | 2020 | No | Yes | No | Not applicable | Yes | No | No | Yes | Yes | Yes | Moderate risk of bias |
| 123 | Mroz | 2017 | No | Yes | Yes | Not applicable | Yes | Unclear | No | Yes | Yes | Yes | Moderate risk of bias |
| 124 | Mroz | 2017 | No | Yes | Yes | Not applicable | Yes | Yes | No | Yes | Yes | Yes | Low risk of bias |
| 125 | Msimang | 2019 | No | Yes | Yes | Not applicable | Yes | No | No | Yes | Yes | Yes | Moderate risk of bias |
| 126 | Muiruri | 2015 | No | Yes | Yes | Yes | Yes | No | Yes | Yes | Yes | Yes | Low risk of bias |
| 127 | Munyua | 2010 | Yes | Yes | Yes | Yes | Yes | No | No | Yes | Yes | Yes | Low risk of bias |
| 128 | Nabeth | 2001 | No | Yes | No | Yes | Yes | No | No | Yes | Yes | Yes | Moderate risk of bias |
| 129 | Nakouné | 2016 | No | Yes | No | Yes | Yes | Yes | No | Yes | Yes | Yes | Low risk of bias |
| 130 | Nakounne | 2001 | No | Yes | No | Yes | Yes | Yes | No | Yes | Yes | Yes | Low risk of bias |
| 131 | Nanyingi | 2017 | No | Yes | Yes | Not applicable | Yes | No | No | Yes | Yes | Yes | Moderate risk of bias |
| 132 | Ndengu | 2020 | No | Yes | Yes | Not applicable | Yes | Unclear | No | Yes | Yes | Yes | Moderate risk of bias |
| 133 | Ndiana | 2019 | No | Yes | Yes | Not applicable | Yes | No | No | Yes | Yes | Yes | Moderate risk of bias |
| 134 | Ndiaye | 2018 | No | Yes | No | Not applicable | Yes | No | Unclear | Yes | Yes | Yes | Moderate risk of bias |
| 135 | Ngoshe | 2020 | No | Yes | Yes | Not applicable | Yes | Unclear | Unclear | Yes | Yes | Yes | Moderate risk of bias |
| 136 | Nguku | 2010 | No | Yes | No | Yes | Yes | Unclear | Unclear | Yes | Yes | Yes | Moderate risk of bias |
| 137 | Njenga | 2009 | No | Yes | No | Yes | Yes | Unclear | Unclear | Yes | Yes | Yes | Moderate risk of bias |
| 138 | Ochieng | 2015 | Yes | Yes | Yes | Yes | Yes | Unclear | Yes | Yes | Yes | Yes | Low risk of bias |
| 139 | Odaibo | 2019 | No | Yes | Yes | Not applicable | Yes | No | No | Yes | Yes | Yes | Moderate risk of bias |
| 140 | O'Hearn | 2016 | No | Yes | No | Yes | Yes | Yes | Yes | Yes | Yes | Yes | Low risk of bias |
| 141 | Olive | 2013 | No | Yes | No | Not applicable | Yes | Yes | No | Yes | Yes | Yes | Moderate risk of bias |
| 142 | Opayele | 2018 | No | Yes | No | Yes | Yes | No | Yes | Yes | Yes | Yes | Low risk of bias |
| 143 | Opayele | 2019 | No | Yes | No | Not applicable | Yes | No | No | Yes | Yes | Yes | Moderate risk of bias |
| 144 | Oragwa | 2022 | No | Yes | No | Yes | Yes | No | No | Yes | Yes | Yes | Moderate risk of bias |
| 145 | Owange | 2014 | No | Yes | Yes | Not applicable | Yes | No | No | Yes | Yes | Yes | Moderate risk of bias |
| 146 | Oyas | 2018 | No | Yes | No | Not applicable | Yes | No | No | Yes | Yes | Yes | Moderate risk of bias |
| 147 | Paweska | 2003 | No | Yes | No | Not applicable | Yes | Unclear | No | Yes | Yes | Yes | Moderate risk of bias |
| 148 | Paweska | 2003 | No | Yes | No | Not applicable | Yes | Unclear | No | Yes | Yes | Yes | Moderate risk of bias |
| 149 | Paweska | 2005 | No | Yes | No | Yes | Yes | Yes | No | Yes | Yes | Yes | Low risk of bias |
| 150 | Paweska | 2005 | No | Yes | No | Yes | Yes | Yes | No | Yes | Yes | Yes | Low risk of bias |
| 151 | Paweska | 2007 | No | Yes | No | Yes | Yes | Yes | No | Yes | Yes | Yes | Low risk of bias |
| 152 | Paweska | 2008 | No | Yes | No | Not applicable | Yes | Yes | No | Yes | Yes | Yes | Moderate risk of bias |
| 153 | Pawęska | 2021 | No | Yes | No | Yes | Yes | Yes | No | Yes | Yes | Yes | Low risk of bias |
| 154 | Peterson | 2017 | No | Yes | Yes | Not applicable | Yes | No | No | Yes | Yes | Yes | Moderate risk of bias |
| 155 | Poueme | 2019 | No | Yes | Yes | Not applicable | Yes | Yes | No | Yes | Yes | Yes | Low risk of bias |
| 156 | Pourrut | 2010 | No | Yes | Yes | Yes | Yes | Yes | Yes | Yes | Yes | Yes | Low risk of bias |
| 157 | Rakotoarivelo | 2011 | No | Yes | No | Yes | Yes | No | No | Yes | Yes | Yes | Moderate risk of bias |
| 158 | Ratovonjato | 2011 | No | Yes | No | Not applicable | Yes | No | No | Yes | Yes | Yes | Moderate risk of bias |
| 159 | Ringot | 2004 | No | Yes | Yes | Not applicable | Yes | No | No | Yes | Yes | Yes | Moderate risk of bias |
| 160 | Rissmann | 2017 | No | Yes | No | Not applicable | Yes | No | No | Yes | Yes | Yes | Moderate risk of bias |
| 161 | Rissmann | 2017 | No | Yes | Yes | Not applicable | Yes | No | No | Yes | Yes | Yes | Moderate risk of bias |
| 162 | Roger | 2011 | No | Yes | Yes | Not applicable | Yes | No | No | Yes | Yes | Yes | Moderate risk of bias |
| 163 | Roger | 2014 | No | Yes | No | Not applicable | Yes | No | No | Yes | Yes | Yes | Moderate risk of bias |
| 164 | Rostal | 2010 | No | Yes | No | Not applicable | Yes | No | No | Yes | Yes | Yes | Moderate risk of bias |
| 165 | Roug | 2020 | No | Yes | No | Not applicable | Yes | Yes | No | Yes | Yes | Yes | Moderate risk of bias |
| 166 | Rugarabamu | 2022 | No | Yes | Yes | Yes | Yes | No | Yes | Yes | Yes | Yes | Low risk of bias |
| 167 | Sadeuh-Mba | 2018 | No | Yes | Yes | Yes | Yes | Yes | No | Yes | Yes | Yes | Low risk of bias |
| 168 | Salekwa | 2019 | No | Yes | No | Not applicable | Yes | No | No | Yes | Yes | Yes | Moderate risk of bias |
| 169 | Sanderson | 2020 | No | Yes | No | Yes | Yes | No | No | Yes | Yes | Yes | Moderate risk of bias |
| 170 | Sang | 2010 | No | Yes | No | Not applicable | Yes | No | No | Yes | Yes | Yes | Moderate risk of bias |
| 171 | Schoepp | 2014 | No | Yes | No | Yes | Yes | Yes | No | Yes | Yes | Yes | Low risk of bias |
| 172 | Schwarz | 2012 | No | Yes | No | Yes | Yes | No | Yes | Yes | Yes | Yes | Low risk of bias |
| 173 | Selmi | 2020 | No | Yes | Yes | Not applicable | Yes | Yes | No | Yes | Yes | Yes | Low risk of bias |
| 174 | Shieh | 2010 | No | Yes | No | Yes | Yes | Yes | No | Yes | Yes | Yes | Low risk of bias |
| 175 | Sindato | 2013 | No | Yes | Yes | Not applicable | Yes | Yes | No | Yes | Yes | Yes | Low risk of bias |
| 176 | Sindato | 2015 | No | Yes | Yes | Not applicable | Yes | No | Unclear | Yes | Yes | Yes | Moderate risk of bias |
| 177 | Soumare | 2007 | No | Yes | No | Not applicable | Yes | Yes | Unclear | Yes | Yes | Unclear | Moderate risk of bias |
| 178 | Sow | 2014 | No | Yes | No | Yes | Yes | No | Unclear | Yes | Yes | Yes | Moderate risk of bias |
| 179 | Sow | 2014 | No | Yes | No | Yes | Yes | No | Unclear | Yes | Yes | Yes | Moderate risk of bias |
| 180 | Sow | 2016 | No | Yes | No | Yes | Yes | No | Unclear | Yes | Yes | Yes | Moderate risk of bias |
| 181 | Sow | 2016 | No | Yes | No | Yes | Yes | Yes | Yes | Yes | Yes | Yes | Low risk of bias |
| 182 | Spiropoulou | 2018 | No | Yes | No | Yes | Yes | No | Unclear | Yes | Yes | Yes | Moderate risk of bias |
| 183 | Sternberg Lewerin | 2018 | No | Yes | Yes | Not applicable | Yes | No | Unclear | Yes | Yes | Yes | Moderate risk of bias |
| 184 | Stoek | 2022 | No | Yes | No | Not applicable | Yes | No | No | Yes | Yes | Yes | Moderate risk of bias |
| 185 | Sumaye | 2013 | No | Yes | Yes | Not applicable | Yes | No | Unclear | Yes | Yes | Yes | Moderate risk of bias |
| 186 | Sumaye | 2015 | No | Yes | Yes | Yes | Yes | No | Unclear | Yes | Yes | Yes | Low risk of bias |
| 187 | Swai | 2009 | No | Yes | No | Yes | Yes | Unclear | Unclear | Yes | Yes | Yes | Moderate risk of bias |
| 188 | Swai | 2015 | No | Yes | No | Not applicable | Yes | No | Unclear | Yes | Yes | Yes | Moderate risk of bias |
| 189 | Tigoi | 2015 | No | Yes | No | Yes | Yes | Yes | Unclear | Yes | Yes | Yes | Low risk of bias |
| 190 | Tigoi | 2020 | No | Yes | Yes | Yes | Yes | Yes | Unclear | Yes | Yes | Yes | Low risk of bias |
| 191 | Traoré-Lamizana | 2001 | No | Yes | No | Not applicable | Yes | Yes | Unclear | Yes | Yes | Yes | Moderate risk of bias |
| 192 | Troupin | 2022 | No | Yes | Yes | Not applicable | Yes | Yes | No | Yes | Yes | Yes | Low risk of bias |
| 193 | Tshilenge | 2019 | No | Yes | Yes | Not applicable | Yes | Yes | Unclear | Yes | Yes | Yes | Low risk of bias |
| 194 | Umuhoza | 2017 | No | Yes | No | Not applicable | Yes | No | Unclear | Yes | Yes | Yes | Moderate risk of bias |
| 195 | Ushijima | 2021 | No | Yes | No | Yes | Yes | Yes | No | Yes | Yes | Yes | Low risk of bias |
| 196 | van den Bergh | 2022 | No | Yes | No | Not applicable | Yes | Yes | No | Yes | Yes | Yes | Moderate risk of bias |
| 197 | Van den Bergh | 2020 | No | Yes | No | Not applicable | Yes | Yes | Unclear | Yes | Yes | Yes | Moderate risk of bias |
| 198 | Wensman | 2015 | No | Yes | No | Not applicable | Yes | No | Yes | Yes | Yes | Yes | Moderate risk of bias |
| 199 | Wolff | 2018 | No | Yes | No | Yes | Yes | No | Unclear | Yes | Yes | Yes | Moderate risk of bias |
| 200 | Woods | 2002 | No | Yes | No | Yes | Yes | No | Unclear | Yes | Yes | Yes | Moderate risk of bias |
| 201 | Youssef | 2001 | No | Yes | No | Not applicable | Yes | Unclear | Unclear | Yes | Yes | Unclear | Moderate risk of bias |
| 202 | Youssef | 2001 | No | Yes | No | Not applicable | Yes | Unclear | Unclear | Yes | Yes | Yes | Moderate risk of bias |
| 203 | Youssef | 2002 | No | Yes | No | Not applicable | Yes | Unclear | Unclear | Unclear | Yes | Yes | Moderate risk of bias |
| 204 | Youssef | 2009 | No | Yes | No | Not applicable | Yes | Unclear | Unclear | Yes | Yes | Yes | Moderate risk of bias |
| 205 | Zouaghi | 2021 | No | Yes | Yes | Not applicable | Yes | Yes | No | Yes | Yes | Yes | Low risk of bias |

# S5 Table. Subgroup analyses of case fatality rate of Rift Valley fever in humans in Africa.

|  | **Prevalence. % (95%CI)** | **95% Prediction interval** | **N Studies** | **N Participants** | **H (95%CI)** | **I² (95%CI)** | **P heterogeneity** | **P difference subtypes** |
| --- | --- | --- | --- | --- | --- | --- | --- | --- |
| **RVF case fatality rate in humans** |  |  |  |  |  |  |  |  |
| **Study Design** |  |  |  |  |  |  |  | 0.997 |
| Community outbreak | 27.9 [0-85.4] | [0-100] | 3 | 503 | 11.9 [9.6-14.9] | 99.3 [98.9-99.5] | <0.001 |  |
| Cross-sectional | 27.8 [20.1-36.1] | [10.8-48.5] | 6 | 195 | 1.2 [1-1.9] | 32 [0-72.5] | 0.196 |  |
| **UNSD Region** |  |  |  |  |  |  |  | 0.375 |
| Eastern Africa | 43.2 [8.9-81.6] | [0-100] | 3 | 263 | 5.5 [3.8-7.8] | 96.6 [93.1-98.4] | <0.001 |  |
| West Africa | 24.4 [14-36.4] | [0.6-62.9] | 5 | 133 | 1.4 [1-2.4] | 51.8 [0-82.3] | 0.081 |  |
| **Setting** |  |  |  |  |  |  |  | 0.806 |
| Community-based | 27.4 [16.6-39.7] | [0-100] | 3 | 94 | 1.2 [1-3.7] | 30.2 [0-92.7] | 0.239 |  |
| Hospital-based | 32.6 [4.1-71] | [0-100] | 5 | 589 | 8.4 [6.9-10.3] | 98.6 [97.9-99.1] | <0.001 |  |
| **Infection Status** |  |  |  |  |  |  |  | 0.441 |
| Current infection | 25.6 [18.5-33.2] | [12.2-41.5] | 5 | 164 | 1.1 [1-2.3] | 10.5 [0-81.4] | 0.346 |  |
| Recent infection | 42.2 [6.5-83.2] | [0-100] | 3 | 232 | 4.7 [3.2-7] | 95.5 [90-97.9] | <0.001 |  |

# S6 Table. Subgroup analyses of prevalence of Rift Valley fever in humans in Africa.

|  | **Prevalence. % (95%CI)** | **95% Prediction interval** | **N Studies** | **N Participants** | **H (95%CI)** | **I² (95%CI)** | **P heterogeneity** | **P difference subtypes** |
| --- | --- | --- | --- | --- | --- | --- | --- | --- |
| **RVF prevalence in humans** |  |  |  |  |  |  |  |  |
| **Study Design** |  |  |  |  |  |  |  | <0.001 |
| Community outbreak | 22.2 [15.5-29.7] | [0.3-61.6] | 21 | 7168 | 6.7 [6.1-7.5] | 97.8 [97.3-98.2] | <0.001 |  |
| Cross-sectional | 5.9 [4.5-7.6] | [0-30.7] | 111 | 95719 | 9.8 [9.4-10.1] | 99 [98.9-99] | <0.001 |  |
| **Sampling** |  |  |  |  |  |  |  | 0.029 |
| Probabilistic | 11.9 [8-16.4] | [0-43.8] | 30 | 21368 | 9.4 [8.7-10] | 98.9 [98.7-99] | <0.001 |  |
| Non probabilistic | 6.9 [5.1-8.9] | [0-35.2] | 103 | 81611 | 10 [9.7-10.4] | 99 [98.9-99.1] | <0.001 |  |
| **Timing of data collection** |  |  |  |  |  |  |  | 0.989 |
| Prospetively | 8 [6.2-9.9] | [0-37.6] | 124 | 94068 | 10.1 [9.7-10.4] | 99 [98.9-99.1] | <0.001 |  |
| Retrospectively | 7.7 [2.6-14.7] | [0-40.6] | 9 | 8911 | 9.7 [8.5-11] | 98.9 [98.6-99.2] | <0.001 |  |
| **Countries** |  |  |  |  |  |  |  | <0.001 |
| Sudan | 48.9 [10.1-88.5] | [0-100] | 4 | 448 | 9.4 [7.6-11.6] | 98.9 [98.3-99.3] | <0.001 |  |
| Kenya | 15.9 [10.8-21.8] | [0-57.1] | 33 | 19262 | 10.2 [9.6-10.8] | 99 [98.9-99.1] | <0.001 |  |
| Mauritania | 14.9 [10.5-19.9] | [1.6-37.6] | 14 | 2111 | 3 [2.4-3.7] | 88.6 [82.6-92.5] | <0.001 |  |
| Nigeria | 12.4 [7-18.9] | [0-49.7] | 4 | 1029 | 2.9 [1.9-4.5] | 88.4 [72.8-95] | <0.001 |  |
| Tanzania | 7.5 [3.2-13.4] | [0-38.5] | 12 | 5813 | 7.3 [6.4-8.4] | 98.1 [97.6-98.6] | <0.001 |  |
| South Africa | 4.2 [1.1-9.1] | [0-29.8] | 9 | 8001 | 8.4 [7.3-9.6] | 98.6 [98.1-98.9] | <0.001 |  |
| Uganda | 3.7 [0.6-9.2] | [0-45.4] | 4 | 3163 | 5.8 [4.3-7.7] | 97 [94.6-98.3] | <0.001 |  |
| Gabon | 3.4 [0.4-8.8] | [0-46.6] | 4 | 5899 | 8 [6.4-10.2] | 98.5 [97.5-99] | <0.001 |  |
| Madagascar | 3.3 [0.1-9.8] | [0-43.4] | 5 | 6043 | 10.2 [8.6-12.1] | 99 [98.6-99.3] | <0.001 |  |
| Central African Republic | 3.2 [0-12.3] | [0-75.3] | 4 | 4184 | 10.8 [8.9-13.1] | 99.1 [98.7-99.4] | <0.001 |  |
| Tunisia | 2.4 [0-10.3] | [0-62.4] | 4 | 438 | 3.1 [2-4.7] | 89.4 [75.7-95.4] | <0.001 |  |
| Cameroon | 1.6 [0-11] | [0-73] | 4 | 258 | 2.7 [1.7-4.2] | 86.4 [67-94.4] | <0.001 |  |
| Mozambique | 1.4 [0-6.4] | [0-100] | 3 | 478 | 2.8 [1.6-4.7] | 86.9 [62.6-95.4] | <0.001 |  |
| Chad | 1.1 [0-6.3] | [0-95.8] | 3 | 93 | 1.3 [1-2.2] | 36.1 [0-79.6] | 0.209 |  |
| Senegal | 0.6 [0.1-1.5] | [0-5.8] | 11 | 34115 | 6.5 [5.6-7.6] | 97.7 [96.9-98.3] | <0.001 |  |
| **UNSD Region** |  |  |  |  |  |  |  | <0.001 |
| Northern Africa | 20 [3-46] | [0-100] | 9 | 929 | 8.4 [7.3-9.7] | 98.6 [98.1-98.9] | <0.001 |  |
| Eastern Africa | 10.3 [7.7-13.2] | [0-40.3] | 63 | 41640 | 9.1 [8.7-9.6] | 98.8 [98.7-98.9] | <0.001 |  |
| West Africa | 5.8 [3.8-8] | [0-23.8] | 35 | 39360 | 7.6 [7.1-8.2] | 98.3 [98-98.5] | <0.001 |  |
| Southern Africa | 4.5 [1.3-9.3] | [0-29.1] | 9 | 7763 | 7.9 [6.9-9.2] | 98.4 [97.9-98.8] | <0.001 |  |
| Central Africa | 2.1 [0.4-4.7] | [0-18.5] | 16 | 10887 | 6.4 [5.6-7.2] | 97.5 [96.8-98.1] | <0.001 |  |
| **Country income level** |  |  |  |  |  |  |  | 0.014 |
| Lower-middle-income economies | 9.3 [6.8-12] | [0-43.3] | 87 | 65817 | 10.7 [10.3-11.1] | 99.1 [99.1-99.2] | <0.001 |  |
| Low-income economies | 5.9 [3.2-9.2] | [0-32.3] | 30 | 16899 | 8 [7.4-8.6] | 98.4 [98.2-98.6] | <0.001 |  |
| Upper-middle-income economies | 3.7 [1.5-6.8] | [0-21.2] | 13 | 14876 | 8.2 [7.3-9.2] | 98.5 [98.1-98.8] | <0.001 |  |
| **Recrutment setting** |  |  |  |  |  |  |  | 0.406 |
| Rural | 7.3 [4.5-10.6] | [0-31.3] | 29 | 22104 | 8.2 [7.6-8.9] | 98.5 [98.3-98.7] | <0.001 |  |
| Urban | 5.5 [2.8-9] | [0-25.9] | 16 | 9999 | 6.6 [5.8-7.4] | 97.7 [97-98.2] | <0.001 |  |
| **Setting** |  |  |  |  |  |  |  | 0.073 |
| Community-based | 8.9 [6.8-11.4] | [0-37.2] | 77 | 43931 | 8.3 [7.9-8.7] | 98.5 [98.4-98.7] | <0.001 |  |
| Hospital-based | 5.8 [3.5-8.6] | [0-32] | 42 | 50618 | 11.2 [10.6-11.8] | 99.2 [99.1-99.3] | <0.001 |  |
| **Hospitalization** |  |  |  |  |  |  |  | 0.186 |
| Ambulatory | 6.7 [1.6-14.7] | [0-49] | 11 | 5413 | 9 [8-10.2] | 98.8 [98.4-99] | <0.001 |  |
| Hospitalized | 12.3 [8.9-16.2] | [1.9-29.4] | 13 | 7347 | 4.1 [3.4-4.9] | 94.1 [91.6-95.9] | <0.001 |  |
| **Study population: Humans** |  |  |  |  |  |  |  | 0.003 |
| RVFV suspected cases | 12.8 [8.9-17.3] | [0-46] | 35 | 17790 | 8.1 [7.5-8.7] | 98.5 [98.2-98.7] | <0.001 |  |
| High risk individuals | 7.4 [5.1-10.1] | [0-23.8] | 24 | 11934 | 4.7 [4.2-5.3] | 95.5 [94.3-96.5] | <0.001 |  |
| Apparently healthy individuals | 7.3 [4.8-10.3] | [0-33.4] | 41 | 33319 | 9.4 [8.9-10] | 98.9 [98.7-99] | <0.001 |  |
| Febrile patients | 4 [2-6.6] | [0-22.3] | 25 | 33802 | 8.3 [7.6-9] | 98.5 [98.3-98.8] | <0.001 |  |
| **Detection assay** |  |  |  |  |  |  |  | <0.001 |
| Classical RT-PCR | 12.4 [3.9-24.5] | [0-69.1] | 11 | 2990 | 8.5 [7.5-9.6] | 98.6 [98.2-98.9] | <0.001 |  |
| Neutralization test | 9.8 [6.2-14.1] | [0.6-27.7] | 7 | 7005 | 4.9 [3.9-6.2] | 95.9 [93.5-97.4] | <0.001 |  |
| Indirect ELISA | 7.8 [5.8-10] | [0-35.2] | 81 | 65647 | 9.7 [9.4-10.1] | 98.9 [98.9-99] | <0.001 |  |
| Indirect immunofluorescence assay | 2.1 [0.1-5.7] | [0-20.2] | 7 | 2988 | 4.1 [3.2-5.3] | 94.1 [90.2-96.4] | <0.001 |  |
| Real Time RT-PCR | 2 [0.5-4.2] | [0-13] | 9 | 17579 | 5.1 [4.2-6.2] | 96.1 [94.3-97.4] | <0.001 |  |
| Hemagglutination inhibition test | 0 [0-0.9] | [0-21.6] | 3 | 217 | 1 [1-3.1] | 0 [0-89.6] | 0.997 |  |
| **Infection Status** |  |  |  |  |  |  |  | 0.013 |
| Current infection | 12.3 [7-18.9] | [0-60.1] | 32 | 24931 | 11.8 [11.1-12.5] | 99.3 [99.2-99.4] | <0.001 |  |
| Past infection | 8.2 [6.3-10.2] | [0-30.1] | 70 | 48294 | 7.5 [7.1-7.9] | 98.2 [98-98.4] | <0.001 |  |
| Recent infection | 4.3 [2.1-7.1] | [0-27.2] | 31 | 29754 | 8.9 [8.3-9.5] | 98.7 [98.5-98.9] | <0.001 |  |

# S7 Table. Subgroup analyses of prevalence of Rift Valley fever in other animal species in Africa.

|  | **Prevalence. % (95%CI)** | **95% Prediction interval** | **N Studies** | **N Participants** | **H (95%CI)** | **I² (95%CI)** | **P heterogeneity** | **P difference subtypes** |
| --- | --- | --- | --- | --- | --- | --- | --- | --- |
| **RVF prevalence in animals** |  |  |  |  |  |  |  |  |
| **Study Design** |  |  |  |  |  |  |  | <0.001 |
| Cross-sectional | 9.9 [8.6-11.3] | [0-41.9] | 318 | 128335 | 7.7 [7.5-7.9] | 98.3 [98.2-98.4] | <0.001 |  |
| Community outbreak | 5 [3.2-7.2] | [0-19.9] | 31 | 7871 | 3.5 [3.1-3.9] | 91.7 [89.3-93.6] | <0.001 |  |
| **Outbreak** |  |  |  |  |  |  |  | <0.001 |
| **Sampling** |  |  |  |  |  |  |  | 0.749 |
| Non probabilistic | 9.2 [7.5-11] | [0-43.5] | 207 | 66385 | 7.2 [7-7.5] | 98.1 [98-98.2] | <0.001 |  |
| Probabilistic | 9.8 [8.1-11.6] | [0-37.4] | 142 | 69821 | 7.5 [7.2-7.7] | 98.2 [98.1-98.3] | <0.001 |  |
| **Timing of data collection** |  |  |  |  |  |  |  | 0.823 |
| Prospetively | 9.5 [8.2-10.8] | [0-40.8] | 323 | 132715 | 7.7 [7.5-7.8] | 98.3 [98.2-98.4] | <0.001 |  |
| Retrospectively | 8.6 [5.2-12.7] | [0-33.7] | 26 | 3491 | 3.4 [3-3.9] | 91.4 [88.7-93.5] | <0.001 |  |
| **Countries** |  |  |  |  |  |  |  | <0.001 |
| Comoros | 30.9 [27.3-34.6] | [21.6-41] | 4 | 763 | 1.1 [1-2.7] | 12.6 [0-86.6] | 0.33 |  |
| Rwanda | 26.4 [14.8-39.8] | [0-100] | 3 | 780 | 2.9 [1.7-4.9] | 88.2 [67.1-95.8] | <0.001 |  |
| Mozambique | 22.6 [15.3-31] | [1-59.9] | 13 | 5902 | 7 [6.2-8] | 98 [97.4-98.4] | <0.001 |  |
| Niger | 21.1 [8.7-36.8] | [0-83.2] | 5 | 638 | 4.1 [3-5.6] | 94.1 [89.1-96.8] | <0.001 |  |
| Zambia | 19.6 [5.6-38.5] | [0-87.9] | 6 | 557 | 4.4 [3.4-5.7] | 94.8 [91.1-96.9] | <0.001 |  |
| Chad | 14 [4.6-27.4] | [0-70.4] | 6 | 1477 | 5.9 [4.7-7.3] | 97.1 [95.5-98.1] | <0.001 |  |
| Mauritania | 13.5 [8.4-19.5] | [0-60.4] | 39 | 5639 | 5.9 [5.5-6.4] | 97.2 [96.7-97.6] | <0.001 |  |
| Tanzania | 12.4 [8.2-17.3] | [0-47.4] | 35 | 8262 | 5.7 [5.2-6.2] | 96.9 [96.3-97.4] | <0.001 |  |
| South Africa | 11.3 [5.7-18.5] | [0-56] | 21 | 11009 | 10 [9.2-10.8] | 99 [98.8-99.1] | <0.001 |  |
| Namibia | 10.5 [2.4-23] | [0-65.3] | 6 | 750 | 4.4 [3.4-5.8] | 94.9 [91.3-97] | <0.001 |  |
| Kenya | 9.7 [6.9-12.9] | [0-38.2] | 54 | 21870 | 6.6 [6.2-7.1] | 97.7 [97.4-98] | <0.001 |  |
| Ethiopia | 9.2 [4-16.1] | [0-39.5] | 9 | 1601 | 3.9 [3.1-4.9] | 93.3 [89.4-95.8] | <0.001 |  |
| Burkina Faso | 9 [4.7-14.3] | [0-39.9] | 4 | 732 | 2.3 [1.4-3.7] | 80.8 [49.8-92.7] | 0.001 |  |
| Egypt | 7.5 [3.8-12.2] | [0-36.8] | 21 | 6974 | 5.2 [4.6-5.9] | 96.3 [95.3-97.1] | <0.001 |  |
| Uganda | 7.4 [3.9-11.8] | [0-28.5] | 10 | 4478 | 4.9 [4.1-5.9] | 95.9 [94-97.2] | <0.001 |  |
| Senegal | 6.8 [0.7-17.4] | [0-58.2] | 9 | 1316 | 5.3 [4.4-6.4] | 96.4 [94.8-97.5] | <0.001 |  |
| Somalia | 6.3 [2-12.3] | [0-33.4] | 9 | 19212 | 10.9 [9.7-12.2] | 99.2 [98.9-99.3] | <0.001 |  |
| Cameroon | 6.1 [3.5-9.4] | [0-19.3] | 8 | 4314 | 3.4 [2.6-4.5] | 91.6 [85.8-95] | <0.001 |  |
| Mali | 5.5 [2.5-9.7] | [0-32.6] | 4 | 1853 | 3.2 [2.1-4.8] | 90.4 [78.3-95.7] | <0.001 |  |
| Nigeria | 5.3 [1.2-11.4] | [0-34.9] | 10 | 1815 | 4.1 [3.3-5] | 94 [90.9-96.1] | <0.001 |  |
| Central African Republic | 4.7 [1.8-8.9] | [0-25] | 6 | 2542 | 4.1 [3.1-5.5] | 94.2 [89.8-96.6] | <0.001 |  |
| Tunisia | 4.6 [0.2-13.5] | [0-52] | 8 | 2169 | 7.3 [6.2-8.6] | 98.1 [97.4-98.7] | <0.001 |  |
| Democratic Republic of the Congo | 4.5 [2.7-6.7] | [0.1-13.9] | 6 | 2920 | 2.6 [1.8-3.7] | 85.3 [69.9-92.8] | <0.001 |  |
| Guinea | 4.2 [0-16.2] | [0-100] | 3 | 1357 | 7.7 [5.8-10.3] | 98.3 [97-99.1] | <0.001 |  |
| Madagascar | 2.3 [0-7.9] | [0-42.7] | 18 | 12720 | 12.1 [11.2-13] | 99.3 [99.2-99.4] | <0.001 |  |
| Zimbabwe | 2.3 [0-9] | [0-50.7] | 4 | 1172 | 2.6 [1.7-4.1] | 85.4 [64-94.1] | <0.001 |  |
| Gabon | 2 [0-7.3] | [0-44.3] | 4 | 402 | 2.6 [1.6-4] | 84.6 [61.6-93.8] | <0.001 |  |
| Ivory Coast | 1.6 [0.1-4.5] | [0-83] | 3 | 686 | 2.2 [1.2-3.9] | 79.4 [34.3-93.5] | 0.008 |  |
| **UNSD Region** |  |  |  |  |  |  |  | 0.013 |
| Southern Africa | 10.9 [6.3-16.6] | [0-51.2] | 29 | 12772 | 8.8 [8.1-9.4] | 98.7 [98.5-98.9] | <0.001 |  |
| Eastern Africa | 10.6 [8.6-12.6] | [0-44.2] | 167 | 78294 | 8.4 [8.1-8.6] | 98.6 [98.5-98.7] | <0.001 |  |
| West Africa | 10.1 [7.3-13.2] | [0-45.4] | 77 | 14036 | 5.6 [5.2-5.9] | 96.8 [96.4-97.1] | <0.001 |  |
| Central Africa | 6.2 [4-8.8] | [0-25.3] | 30 | 11655 | 5.2 [4.7-5.7] | 96.2 [95.4-96.9] | <0.001 |  |
| Northern Africa | 5.9 [3.1-9.4] | [0-35.3] | 34 | 11322 | 6.4 [5.9-7] | 97.6 [97.1-97.9] | <0.001 |  |
| **Country income level** |  |  |  |  |  |  |  | 0.602 |
| Low-income economies | 9 [6.8-11.5] | [0-41.9] | 97 | 56452 | 9.3 [9-9.7] | 98.8 [98.8-98.9] | <0.001 |  |
| Lower-middle-income economies | 9.9 [8.4-11.6] | [0-39.4] | 205 | 57596 | 5.9 [5.7-6.2] | 97.2 [97-97.4] | <0.001 |  |
| Upper-middle-income economies | 8.6 [4.8-13.2] | [0-46.7] | 35 | 14031 | 8.4 [7.9-9] | 98.6 [98.4-98.8] | <0.001 |  |
| **Recrutment setting** |  |  |  |  |  |  |  | 0.142 |
| Rural | 8.3 [6.5-10.2] | [0-37] | 136 | 40937 | 6.2 [5.9-6.5] | 97.4 [97.2-97.6] | <0.001 |  |
| Urban | 4.5 [1.1-9.6] | [0-36.5] | 16 | 2901 | 5.1 [4.5-5.9] | 96.2 [95-97.1] | <0.001 |  |
| **Study population: Other animal species** |  |  |  |  |  |  |  | <0.001 |
| Impala | 25.3 [1-64.3] | [0-100] | 3 | 167 | 4.8 [3.3-7.1] | 95.7 [90.6-98] | <0.001 |  |
| Livestock (goat, sheep, cattle) | 19.2 [9.5-31.3] | [0-71.4] | 5 | 7489 | 11.4 [9.7-13.4] | 99.2 [98.9-99.4] | <0.001 |  |
| Springbok | 18.6 [6.3-35.3] | [0-100] | 3 | 600 | 4.8 [3.2-7] | 95.6 [90.3-98] | <0.001 |  |
| Small ruminants (Goat and Sheep) | 17.7 [9.5-27.8] | [0-67.3] | 15 | 6922 | 10 [9.1-11] | 99 [98.8-99.2] | <0.001 |  |
| Camel | 13.4 [8-20] | [0-56.1] | 28 | 4530 | 5.6 [5-6.2] | 96.8 [96.1-97.4] | <0.001 |  |
| Buffalo | 10.3 [6.9-14.2] | [0.1-31.1] | 20 | 4557 | 3.6 [3.1-4.2] | 92.4 [89.6-94.4] | <0.001 |  |
| Goat | 10.2 [7.2-13.5] | [0-45.4] | 65 | 25927 | 8 [7.6-8.4] | 98.4 [98.3-98.6] | <0.001 |  |
| Cattle | 10 [7.6-12.7] | [0-41.2] | 80 | 45443 | 8.8 [8.4-9.2] | 98.7 [98.6-98.8] | <0.001 |  |
| Sheep | 9 [6.4-11.9] | [0-39.2] | 65 | 20820 | 6.4 [6-6.8] | 97.6 [97.3-97.8] | <0.001 |  |
| Rattus rattus | 8.9 [0-34.5] | [0-100] | 3 | 1071 | 10.7 [8.4-13.5] | 99.1 [98.6-99.5] | <0.001 |  |
| Kudu | 6.7 [0-38.8] | [0-100] | 3 | 78 | 3 [1.8-5] | 89 [69.8-96] | <0.001 |  |
| Wildebeest | 2.4 [0-10.5] | [0-41.4] | 7 | 261 | 2.4 [1.7-3.3] | 82.1 [64.3-91] | <0.001 |  |
| Warthog (Phaecochoerus africanus) | 2.2 [0.2-5.7] | [0-15.3] | 6 | 351 | 1.5 [1-2.3] | 54.4 [0-81.7] | 0.052 |  |
| Rodents | 0.6 [0-3.2] | [0-20.5] | 4 | 359 | 1.7 [1-2.9] | 65 [0-88.1] | 0.036 |  |
| Zebra | 0.2 [0-2] | [0-29.1] | 3 | 179 | 1 [1-3.1] | 0 [0-89.6] | 0.833 |  |
| **Type of animal** |  |  |  |  |  |  |  | 0.012 |
| Domesticated animal | 10.4 [9-12] | [0-42.9] | 263 | 116552 | 8.1 [7.9-8.3] | 98.5 [98.4-98.6] | <0.001 |  |
| Wild animal | 6.4 [4.4-8.6] | [0-33.6] | 86 | 19654 | 4.7 [4.4-5] | 95.5 [94.8-96] | <0.001 |  |
| **Other animal species orders** |  |  |  |  |  |  |  | <0.001 |
| Artiodactyla | 10.2 [8.9-11.6] | [0-42.4] | 313 | 123065 | 7.6 [7.4-7.8] | 98.3 [98.2-98.4] | <0.001 |  |
| Perissodactyla | 5.5 [0-21.3] | [0-80.2] | 7 | 686 | 5.9 [4.8-7.2] | 97.1 [95.7-98.1] | <0.001 |  |
| Rodentia | 1.2 [0-6.8] | [0-38.2] | 13 | 1556 | 4.7 [4-5.6] | 95.5 [93.7-96.8] | <0.001 |  |
| Afrosoricida | 0 [0-0.2] | [0-1.8] | 4 | 343 | 1 [1-2.6] | 0 [0-84.7] | 0.882 |  |
| **Detection assay** |  |  |  |  |  |  |  | <0.001 |
| Classical RT-PCR | 20.5 [11.3-31.3] | [0-58.9] | 7 | 607 | 2.5 [1.8-3.5] | 84.3 [69.3-91.9] | <0.001 |  |
| Neutralization test | 11.4 [7.8-15.6] | [0-49] | 52 | 18148 | 7.5 [7.1-8] | 98.2 [98-98.4] | <0.001 |  |
| Indirect ELISA | 9.8 [8.4-11.3] | [0-41.2] | 265 | 111400 | 7.7 [7.5-7.9] | 98.3 [98.2-98.4] | <0.001 |  |
| Culture | 1.3 [0-4.4] | [0-17] | 5 | 411 | 1.7 [1.1-2.8] | 66.9 [14.2-87.3] | 0.017 |  |
| Real Time RT-PCR | 0.1 [0-1.7] | [0-10.1] | 10 | 1101 | 2.3 [1.7-3] | 80.7 [65.4-89.2] | <0.001 |  |
| **Infection Status** |  |  |  |  |  |  |  | <0.001 |
| Past infection | 10.7 [9.4-12.1] | [0-40.1] | 275 | 104565 | 6.8 [6.7-7] | 97.9 [97.7-98] | <0.001 |  |
| Current infection | 3.6 [1.7-6] | [0-20.3] | 28 | 5347 | 3.4 [3-3.9] | 91.5 [88.9-93.5] | <0.001 |  |
| Recent infection | 6 [4-8.3] | [0-26.7] | 46 | 26294 | 6.5 [6.1-7] | 97.7 [97.3-98] | <0.001 |  |
